# Supplementary material for: Transcriptomic response of maize primary roots to low temperatures at seedling emergence
Source: PeerJ. 2017 Jan 5;5:e2839. doi: 10.7717/peerj.2839 (PMC5289442; doi:10.7717/peerj.2839)
Supplement: Table S1 — Maize varieties differed in the type of kernel and maturity group. Some varieties were included in the Irish Recommended List 2008 for showing high performance under Irish climate conditions. (*varieties included in the Irish Recommended List 2008 suitable for growing in the open/without plastic; **varieties included in the Irish Recommended List 2008 suitable for growing covered/with plastic; varieties marked in bold were included in the microarray study). Germination rates under control and cold conditions are listed, including standard deviations (in brackets). [file peerj-05-2839-s001.docx]

Supplemental Materials Table 1. Maize varieties included in this study. Maize varieties differed in the type of kernel and maturity group. Some varieties were included in the Irish Recommended List 2008 for showing high performance under Irish climate conditions. (*varieties included in the Irish Recommended List 2008 suitable for growing in the open/without plastic; **varieties included in the Irish Recommended List 2008 suitable for growing covered/with plastic; varieties marked in bold were included in the microarray study). Germination rates under control and cold conditions are listed, including standard deviations (in brackets).

| **Variety** | **Maturity group** | **Kernel type** | **Breeding Company** | **germination rate control conditions***** | **germination rate cold conditions***** |
| --- | --- | --- | --- | --- | --- |
| **Picker** | Very early | Flint-Dent | Caussade | 0.99 (0.025) | 0.92 (0.071) |
| **PR39B29** | Very early | Flint | Pioneer | 0.78 (0.136) | 0.56 (0.257) |
| Huski | Very early | Flint-Dent | Caussade | 0.92 (0.107) | 0.81 (0.105) |
| Codifar | Early | Flint-Dent | Codisem | 0.98 (0.029) | 0.88 (0.103) |
| Justina** | Early | Flint-Dent | Pioneer | 0.85 (0.109) | 0.21 (0.051) |
| PR39D60** | Early | Flint | Pioneer | 0.78 (0.136) | 0.58 (0.077) |
| Lakti | Early | Flint-Dent | Caussade | 0.98 (0.025) | 0.67 (0.244) |
| **Fergus*** | Early | Dent | Caussade | 0.99 (0.013) | 0.43 (0.278) |
| **Codisco** | Mid-early | Flint-Dent | Codisem | 0.94 (0.034) | 0.56 (0.155) |
| Crazi | Mid-early | Flint-Dent | Caussade | 0.86 (0.131) | 0.83 (0.111) |
| Clariti | Mid-early | Flint-Dent | Caussade | 0.96 (0.027) | 0.76 (0.034) |
| Algans* | Mid-early | Flint-Dent | Caussade | 0.87 (0.002) | 0.38 (0.038) |

*** standard deviation in brackets
