# Supplementary material for: Transcriptomic response of maize primary roots to low temperatures at seedling emergence
Source: PeerJ. 2017 Jan 5;5:e2839. doi: 10.7717/peerj.2839 (PMC5289442; doi:10.7717/peerj.2839)

Supplemental Tables 2: Original data and tables of data generated by the Statistical Analysis Software (SAS).

Original data used for calculations:

| **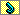NAME** | **TREAT** | **REP** | **ROOT** | **SHOOT** | **TIME** | **EXP** |
| --- | --- | --- | --- | --- | --- | --- |
| CRAZI | control | 1 | 0 | 0 | 1 | 1 |
| CRAZI | control | 2 | 0.4 | 0.41 | 1 | 1 |
| HUSKI | control | 1 | 0.08 | 0 | 1 | 1 |
| HUSKI | control | 2 | 0.43 | 0.2 | 1 | 1 |
| LAKTI | control | 1 | 0.23 | 0.16 | 1 | 1 |
| LAKTI | control | 2 | 0.18 | 0 | 1 | 1 |
| ALGANS | control | 1 | 1.81 | 0.51 | 1 | 1 |
| ALGANS | control | 2 | 0.31 | 0.26 | 1 | 1 |
| PICKER | control | 1 | 1.18 | 0 | 1 | 1 |
| PICKER | control | 2 | 0.54 | 0 | 1 | 1 |
| FERGUS | control | 1 | 1.03 | 0 | 1 | 1 |
| FERGUS | control | 2 | 0.84 | 0 | 1 | 1 |
| CODISCO | control | 1 | 1.29 | 0.16 | 1 | 1 |
| CODISCO | control | 2 | 1.61 | 0 | 1 | 1 |
| CODIFAR | control | 1 | 0.13 | 0 | 1 | 1 |
| CODIFAR | control | 2 | 0.98 | 0 | 1 | 1 |
| CLARITI | control | 1 | 0.84 | 0 | 1 | 1 |
| CLARITI | control | 2 | 1.09 | 0 | 1 | 1 |
| JUSTINA | control | 1 | 0.59 | 0.48 | 1 | 1 |
| JUSTINA | control | 2 | 0.5 | 0.29 | 1 | 1 |
| PR29B29 | control | 1 | 0.08 | 0 | 1 | 1 |
| PR29B29 | control | 2 | 0.98 | 0 | 1 | 1 |
| P329D60 | control | 1 | 0.18 | 0 | 1 | 1 |
| P329D60 | control | 2 | 0.25 | 0 | 1 | 1 |
| CRAZI | cold | 1 | 1.16 | 0.92 | 1 | 1 |
| CRAZI | cold | 2 | 1.01 | 0.46 | 1 | 1 |
| HUSKI | cold | 1 | 0.22 | 0 | 1 | 1 |
| HUSKI | cold | 2 | 0.44 | 0 | 1 | 1 |
| LAKTI | cold | 1 | 0.71 | 0 | 1 | 1 |
| LAKTI | cold | 2 | 0.7 | 0 | 1 | 1 |
| ALGANS | cold | 1 | 0 | 0 | 1 | 1 |
| ALGANS | cold | 2 | 0 | 0.11 | 1 | 1 |
| PICKER | cold | 1 | 1.25 | 0 | 1 | 1 |
| PICKER | cold | 2 | 4.03 | 0.43 | 1 | 1 |
| FERGUS | cold | 1 | 0 | 0 | 1 | 1 |
| FERGUS | cold | 2 | 0.56 | 0 | 1 | 1 |
| CODISCO | cold | 1 | 0.27 | 0 | 1 | 1 |
| CODISCO | cold | 2 | 0.22 | 0 | 1 | 1 |
| CODIFAR | cold | 1 | 1.24 | 0 | 1 | 1 |
| CODIFAR | cold | 2 | 2.08 | 0 | 1 | 1 |
| CLARITI | cold | 1 | 1.02 | 0.22 | 1 | 1 |
| CLARITI | cold | 2 | 1.23 | 0.22 | 1 | 1 |
| JUSTINA | cold | 1 | 0.41 | 0.35 | 1 | 1 |
| JUSTINA | cold | 2 | 0 | 0 | 1 | 1 |
| PR29B29 | cold | 1 | 0.31 | 0.1 | 1 | 1 |
| PR29B29 | cold | 2 | 0.39 | 0.23 | 1 | 1 |
| P329D60 | cold | 1 | 0.48 | 0 | 1 | 1 |
| P329D60 | cold | 2 | 0.07 | 0 | 1 | 1 |
| CRAZI | control | 1 | 0.28 | 0 | 2 | 1 |
| CRAZI | control | 2 | 3.61 | 0.97 | 2 | 1 |
| HUSKI | control | 1 | 0.35 | 0.12 | 2 | 1 |
| HUSKI | control | 2 | 4.17 | 1.94 | 2 | 1 |
| LAKTI | control | 1 | 3.25 | 0.77 | 2 | 1 |
| LAKTI | control | 2 | 3.75 | 0.36 | 2 | 1 |
| ALGANS | control | 1 | 3.74 | 0.91 | 2 | 1 |
| ALGANS | control | 2 | 1.69 | 0.3 | 2 | 1 |
| PICKER | control | 1 | 6.54 | 1.24 | 2 | 1 |
| PICKER | control | 2 | 4.17 | 0.37 | 2 | 1 |
| FERGUS | control | 1 | 5.32 | 1.09 | 2 | 1 |
| FERGUS | control | 2 | 6.71 | 2.8 | 2 | 1 |
| CODISCO | control | 1 | 5.28 | 0.85 | 2 | 1 |
| CODISCO | control | 2 | 5.82 | 2.07 | 2 | 1 |
| CODIFAR | control | 1 | 1.55 | 0 | 2 | 1 |
| CODIFAR | control | 2 | 5.4 | 0.65 | 2 | 1 |
| CLARITI | control | 1 | 4.68 | 1.42 | 2 | 1 |
| CLARITI | control | 2 | 5.48 | 1.64 | 2 | 1 |
| JUSTINA | control | 1 | 2.26 | 0 | 2 | 1 |
| JUSTINA | control | 2 | 1.98 | 0.19 | 2 | 1 |
| PR29B29 | control | 1 | 0.24 | 0 | 2 | 1 |
| PR29B29 | control | 2 | 1.6 | 0 | 2 | 1 |
| P329D60 | control | 1 | 2.89 | 0 | 2 | 1 |
| P329D60 | control | 2 | 3.64 | 0.25 | 2 | 1 |
| CRAZI | cold | 1 | 2.02 | 1.78 | 2 | 1 |
| CRAZI | cold | 2 | 2.06 | 1.75 | 2 | 1 |
| HUSKI | cold | 1 | 0.34 | 0 | 2 | 1 |
| HUSKI | cold | 2 | 0.69 | 0 | 2 | 1 |
| LAKTI | cold | 1 | 2.1 | 0.64 | 2 | 1 |
| LAKTI | cold | 2 | 1.92 | 0.25 | 2 | 1 |
| ALGANS | cold | 1 | 0.05 | 0.28 | 2 | 1 |
| ALGANS | cold | 2 | 0.23 | 0.43 | 2 | 1 |
| PICKER | cold | 1 | 3.06 | 0 | 2 | 1 |
| PICKER | cold | 2 | 6.1 | 0 | 2 | 1 |
| FERGUS | cold | 1 | 0.41 | 0 | 2 | 1 |
| FERGUS | cold | 2 | 1.63 | 0.39 | 2 | 1 |
| CODISCO | cold | 1 | 0.93 | 0.32 | 2 | 1 |
| CODISCO | cold | 2 | 0.9 | 0.52 | 2 | 1 |
| CODIFAR | cold | 1 | 2.53 | 0.27 | 2 | 1 |
| CODIFAR | cold | 2 | 4.4 | 1.09 | 2 | 1 |
| CLARITI | cold | 1 | 1.78 | 0.45 | 2 | 1 |
| CLARITI | cold | 2 | 2.04 | 0.78 | 2 | 1 |
| JUSTINA | cold | 1 | 0.14 | 0.12 | 2 | 1 |
| JUSTINA | cold | 2 | 0 | 0 | 2 | 1 |
| PR29B29 | cold | 1 | 0.99 | 0.41 | 2 | 1 |
| PR29B29 | cold | 2 | 1.12 | 0.57 | 2 | 1 |
| P329D60 | cold | 1 | 1.51 | 0 | 2 | 1 |
| P329D60 | cold | 2 | 0.47 | 0 | 2 | 1 |
| CRAZI | control | 1 | 1.49 | 0 | 3 | 1 |
| CRAZI | control | 2 | 8.74 | 1.4 | 3 | 1 |
| HUSKI | control | 1 | 1.41 | 0.13 | 3 | 1 |
| HUSKI | control | 2 | 8.61 | 2.75 | 3 | 1 |
| LAKTI | control | 1 | 5.1 | 1.03 | 3 | 1 |
| LAKTI | control | 2 | 6.64 | 0.42 | 3 | 1 |
| ALGANS | control | 1 | 7.05 | 3.01 | 3 | 1 |
| ALGANS | control | 2 | 4.62 | 1.93 | 3 | 1 |
| PICKER | control | 1 | 13.71 | 1.98 | 3 | 1 |
| PICKER | control | 2 | 12.29 | 0.97 | 3 | 1 |
| FERGUS | control | 1 | 9.92 | 1.44 | 3 | 1 |
| FERGUS | control | 2 | 13.39 | 4.23 | 3 | 1 |
| CODISCO | control | 1 | 7.34 | 0.94 | 3 | 1 |
| CODISCO | control | 2 | 9.68 | 2.4 | 3 | 1 |
| CODIFAR | control | 1 | 2.71 | 0 | 3 | 1 |
| CODIFAR | control | 2 | 10.19 | 0.69 | 3 | 1 |
| CLARITI | control | 1 | 10.12 | 1.73 | 3 | 1 |
| CLARITI | control | 2 | 10.42 | 2.45 | 3 | 1 |
| JUSTINA | control | 1 | 4.11 | 0.97 | 3 | 1 |
| JUSTINA | control | 2 | 4.46 | 1.12 | 3 | 1 |
| PR29B29 | control | 1 | 2.07 | 0.16 | 3 | 1 |
| PR29B29 | control | 2 | 3.71 | 0.52 | 3 | 1 |
| P329D60 | control | 1 | 4.81 | 0 | 3 | 1 |
| P329D60 | control | 2 | 7.88 | 0.7 | 3 | 1 |
| CRAZI | cold | 1 | 4.24 | 2.46 | 3 | 1 |
| CRAZI | cold | 2 | 4.1 | 2.29 | 3 | 1 |
| HUSKI | cold | 1 | 3.42 | 0.14 | 3 | 1 |
| HUSKI | cold | 2 | 3.48 | 0 | 3 | 1 |
| LAKTI | cold | 1 | 3.27 | 0.69 | 3 | 1 |
| LAKTI | cold | 2 | 3.01 | 0.25 | 3 | 1 |
| ALGANS | cold | 1 | 0.12 | 0.29 | 3 | 1 |
| ALGANS | cold | 2 | 0.32 | 0.47 | 3 | 1 |
| PICKER | cold | 1 | 6.11 | 1.1 | 3 | 1 |
| PICKER | cold | 2 | 8.95 | 1.65 | 3 | 1 |
| FERGUS | cold | 1 | 0.73 | 0 | 3 | 1 |
| FERGUS | cold | 2 | 2.54 | 0.4 | 3 | 1 |
| CODISCO | cold | 1 | 1.3 | 0.34 | 3 | 1 |
| CODISCO | cold | 2 | 1.14 | 0.58 | 3 | 1 |
| CODIFAR | cold | 1 | 5.32 | 0.28 | 3 | 1 |
| CODIFAR | cold | 2 | 6.19 | 1.15 | 3 | 1 |
| CLARITI | cold | 1 | 2.49 | 0.59 | 3 | 1 |
| CLARITI | cold | 2 | 3.37 | 0.94 | 3 | 1 |
| JUSTINA | cold | 1 | 1.09 | 0.57 | 3 | 1 |
| JUSTINA | cold | 2 | 0.26 | 0 | 3 | 1 |
| PR29B29 | cold | 1 | 1.75 | 0.44 | 3 | 1 |
| PR29B29 | cold | 2 | 1.75 | 0.6 | 3 | 1 |
| P329D60 | cold | 1 | 3.16 | 1.19 | 3 | 1 |
| P329D60 | cold | 2 | 1.35 | 0.89 | 3 | 1 |
| CRAZI | control | 1 | 4.18 | 0.98 | 4 | 1 |
| CRAZI | control | 2 | 13.99 | 4.68 | 4 | 1 |
| HUSKI | control | 1 | 4.9 | 1.16 | 4 | 1 |
| HUSKI | control | 2 | 13.02 | 4.05 | 4 | 1 |
| LAKTI | control | 1 | 9.35 | 2.24 | 4 | 1 |
| LAKTI | control | 2 | 9.69 | 1.57 | 4 | 1 |
| ALGANS | control | 1 | 9.36 | 3.84 | 4 | 1 |
| ALGANS | control | 2 | 7.67 | 3.07 | 4 | 1 |
| PICKER | control | 1 | 20.56 | 5.32 | 4 | 1 |
| PICKER | control | 2 | 20.56 | 5.44 | 4 | 1 |
| FERGUS | control | 1 | 14.7 | 4.43 | 4 | 1 |
| FERGUS | control | 2 | 21.2 | 7.18 | 4 | 1 |
| CODISCO | control | 1 | 9.61 | 1.62 | 4 | 1 |
| CODISCO | control | 2 | 14.8 | 4.01 | 4 | 1 |
| CODIFAR | control | 1 | 5.48 | 0.26 | 4 | 1 |
| CODIFAR | control | 2 | 14.33 | 1.72 | 4 | 1 |
| CLARITI | control | 1 | 15.78 | 3.7 | 4 | 1 |
| CLARITI | control | 2 | 15.6 | 4.83 | 4 | 1 |
| JUSTINA | control | 1 | 7.08 | 2.03 | 4 | 1 |
| JUSTINA | control | 2 | 7.54 | 1.53 | 4 | 1 |
| PR29B29 | control | 1 | 4.57 | 0.84 | 4 | 1 |
| PR29B29 | control | 2 | 5.3 | 1.31 | 4 | 1 |
| P329D60 | control | 1 | 7.83 | 0.15 | 4 | 1 |
| P329D60 | control | 2 | 12.41 | 2.84 | 4 | 1 |
| CRAZI | cold | 1 | 5.93 | 3.03 | 4 | 1 |
| CRAZI | cold | 2 | 5.45 | 2.71 | 4 | 1 |
| HUSKI | cold | 1 | 4.15 | 0.15 | 4 | 1 |
| HUSKI | cold | 2 | 4.32 | 0 | 4 | 1 |
| LAKTI | cold | 1 | 5.99 | 1.32 | 4 | 1 |
| LAKTI | cold | 2 | 5.59 | 0.31 | 4 | 1 |
| ALGANS | cold | 1 | 0.88 | 0.64 | 4 | 1 |
| ALGANS | cold | 2 | 1.14 | 0.55 | 4 | 1 |
| PICKER | cold | 1 | 8.08 | 1.14 | 4 | 1 |
| PICKER | cold | 2 | 10.86 | 1.76 | 4 | 1 |
| FERGUS | cold | 1 | 1.66 | 0.53 | 4 | 1 |
| FERGUS | cold | 2 | 5.05 | 0.92 | 4 | 1 |
| CODISCO | cold | 1 | 2.15 | 0.38 | 4 | 1 |
| CODISCO | cold | 2 | 1.97 | 1.05 | 4 | 1 |
| CODIFAR | cold | 1 | 8.75 | 3.4 | 4 | 1 |
| CODIFAR | cold | 2 | 8.57 | 2.57 | 4 | 1 |
| CLARITI | cold | 1 | 3.59 | 1.44 | 4 | 1 |
| CLARITI | cold | 2 | 5.44 | 1.78 | 4 | 1 |
| JUSTINA | cold | 1 | 1.34 | 0.6 | 4 | 1 |
| JUSTINA | cold | 2 | 0.28 | 0 | 4 | 1 |
| PR29B29 | cold | 1 | 3.08 | 1.11 | 4 | 1 |
| PR29B29 | cold | 2 | 3.44 | 1.11 | 4 | 1 |
| P329D60 | cold | 1 | 4.85 | 1.27 | 4 | 1 |
| P329D60 | cold | 2 | 2.01 | 0.93 | 4 | 1 |
| CRAZI | control | 1 | 9.45 | 3.4 | 5 | 1 |
| CRAZI | control | 2 | 19.74 | 6.84 | 5 | 1 |
| HUSKI | control | 1 | 10.18 | 3.25 | 5 | 1 |
| HUSKI | control | 2 | 17.29 | 5.47 | 5 | 1 |
| LAKTI | control | 1 | 13.58 | 4.96 | 5 | 1 |
| LAKTI | control | 2 | 14.7 | 4.47 | 5 | 1 |
| ALGANS | control | 1 | 12.49 | 5.86 | 5 | 1 |
| ALGANS | control | 2 | 8.74 | 3.31 | 5 | 1 |
| PICKER | control | 1 | 27.16 | 7.63 | 5 | 1 |
| PICKER | control | 2 | 26.43 | 7.63 | 5 | 1 |
| FERGUS | control | 1 | 19.24 | 6.4 | 5 | 1 |
| FERGUS | control | 2 | 29.58 | 10.83 | 5 | 1 |
| CODISCO | control | 1 | 12.13 | 2.33 | 5 | 1 |
| CODISCO | control | 2 | 19.65 | 6.07 | 5 | 1 |
| CODIFAR | control | 1 | 9.75 | 1.59 | 5 | 1 |
| CODIFAR | control | 2 | 18.4 | 4.26 | 5 | 1 |
| CLARITI | control | 1 | 21.42 | 5.41 | 5 | 1 |
| CLARITI | control | 2 | 20.43 | 7.04 | 5 | 1 |
| JUSTINA | control | 1 | 8.72 | 2.17 | 5 | 1 |
| JUSTINA | control | 2 | 10.66 | 2.73 | 5 | 1 |
| PR29B29 | control | 1 | 9.97 | 1.8 | 5 | 1 |
| PR29B29 | control | 2 | 7.28 | 1.48 | 5 | 1 |
| P329D60 | control | 1 | 12.8 | 2.27 | 5 | 1 |
| P329D60 | control | 2 | 18.58 | 5.15 | 5 | 1 |
| CRAZI | cold | 1 | 7.86 | 3.28 | 5 | 1 |
| CRAZI | cold | 2 | 7.24 | 2.81 | 5 | 1 |
| HUSKI | cold | 1 | 5.78 | 0.57 | 5 | 1 |
| HUSKI | cold | 2 | 5.92 | 0 | 5 | 1 |
| LAKTI | cold | 1 | 7.84 | 1.35 | 5 | 1 |
| LAKTI | cold | 2 | 7.21 | 0.33 | 5 | 1 |
| ALGANS | cold | 1 | 1.04 | 0.7 | 5 | 1 |
| ALGANS | cold | 2 | 1.45 | 0.59 | 5 | 1 |
| PICKER | cold | 1 | 10.65 | 1.97 | 5 | 1 |
| PICKER | cold | 2 | 13.35 | 2.4 | 5 | 1 |
| FERGUS | cold | 1 | 2.91 | 0.58 | 5 | 1 |
| FERGUS | cold | 2 | 6.3 | 0.98 | 5 | 1 |
| CODISCO | cold | 1 | 2.87 | 0.39 | 5 | 1 |
| CODISCO | cold | 2 | 2.67 | 1.12 | 5 | 1 |
| CODIFAR | cold | 1 | 11.98 | 3.62 | 5 | 1 |
| CODIFAR | cold | 2 | 10.2 | 2.6 | 5 | 1 |
| CLARITI | cold | 1 | 4.62 | 1.65 | 5 | 1 |
| CLARITI | cold | 2 | 6.14 | 1.89 | 5 | 1 |
| JUSTINA | cold | 1 | 1.75 | 0.75 | 5 | 1 |
| JUSTINA | cold | 2 | 0.56 | 0.3 | 5 | 1 |
| PR29B29 | cold | 1 | 4.37 | 1.18 | 5 | 1 |
| PR29B29 | cold | 2 | 4.94 | 1.16 | 5 | 1 |
| P329D60 | cold | 1 | 6.96 | 1.36 | 5 | 1 |
| P329D60 | cold | 2 | 3.11 | 0.98 | 5 | 1 |
| CRAZI | control | 1 | 13.43 | 4.33 | 6 | 1 |
| CRAZI | control | 2 | 24.63 | 8.36 | 6 | 1 |
| HUSKI | control | 1 | 14.11 | 3.94 | 6 | 1 |
| HUSKI | control | 2 | 18.51 | 6.08 | 6 | 1 |
| LAKTI | control | 1 | 18.87 | 8.62 | 6 | 1 |
| LAKTI | control | 2 | 18.54 | 5.84 | 6 | 1 |
| ALGANS | control | 1 | 17.76 | 8.27 | 6 | 1 |
| ALGANS | control | 2 | 10.87 | 4.22 | 6 | 1 |
| PICKER | control | 1 | 28.62 | 8.03 | 6 | 1 |
| PICKER | control | 2 | 30.05 | 8.76 | 6 | 1 |
| FERGUS | control | 1 | 21.86 | 7.16 | 6 | 1 |
| FERGUS | control | 2 | 34.51 | 12.94 | 6 | 1 |
| CODISCO | control | 1 | 13.09 | 3.05 | 6 | 1 |
| CODISCO | control | 2 | 22.01 | 6.35 | 6 | 1 |
| CODIFAR | control | 1 | 16.61 | 2.69 | 6 | 1 |
| CODIFAR | control | 2 | 22.83 | 5.43 | 6 | 1 |
| CLARITI | control | 1 | 23.83 | 6.08 | 6 | 1 |
| CLARITI | control | 2 | 23.41 | 8.96 | 6 | 1 |
| JUSTINA | control | 1 | 9.85 | 2.51 | 6 | 1 |
| JUSTINA | control | 2 | 13.11 | 4.17 | 6 | 1 |
| PR29B29 | control | 1 | 16.73 | 4.63 | 6 | 1 |
| PR29B29 | control | 2 | 10.26 | 2.9 | 6 | 1 |
| P329D60 | control | 1 | 19.34 | 3.78 | 6 | 1 |
| P329D60 | control | 2 | 23.63 | 6.1 | 6 | 1 |
| CRAZI | cold | 1 | 9.21 | 3.6 | 6 | 1 |
| CRAZI | cold | 2 | 8.46 | 2.99 | 6 | 1 |
| HUSKI | cold | 1 | 6.28 | 0.62 | 6 | 1 |
| HUSKI | cold | 2 | 6.59 | 0 | 6 | 1 |
| LAKTI | cold | 1 | 10.32 | 2.18 | 6 | 1 |
| LAKTI | cold | 2 | 9.54 | 0.74 | 6 | 1 |
| ALGANS | cold | 1 | 1.96 | 1.2 | 6 | 1 |
| ALGANS | cold | 2 | 2.1 | 1.76 | 6 | 1 |
| PICKER | cold | 1 | 13.01 | 2.11 | 6 | 1 |
| PICKER | cold | 2 | 15.19 | 2.63 | 6 | 1 |
| FERGUS | cold | 1 | 4.83 | 1.1 | 6 | 1 |
| FERGUS | cold | 2 | 8.09 | 1.5 | 6 | 1 |
| CODISCO | cold | 1 | 4.25 | 0.55 | 6 | 1 |
| CODISCO | cold | 2 | 3.7 | 1.48 | 6 | 1 |
| CODIFAR | cold | 1 | 15.81 | 4.03 | 6 | 1 |
| CODIFAR | cold | 2 | 12.42 | 3.05 | 6 | 1 |
| CLARITI | cold | 1 | 6.6 | 2.82 | 6 | 1 |
| CLARITI | cold | 2 | 7.37 | 1.96 | 6 | 1 |
| JUSTINA | cold | 1 | 2.42 | 0.8 | 6 | 1 |
| JUSTINA | cold | 2 | 1.05 | 0.33 | 6 | 1 |
| PR29B29 | cold | 1 | 6.35 | 1.99 | 6 | 1 |
| PR29B29 | cold | 2 | 7.03 | 2.6 | 6 | 1 |
| P329D60 | cold | 1 | 8.9 | 1.53 | 6 | 1 |
| P329D60 | cold | 2 | 5.83 | 1.04 | 6 | 1 |
| CRAZI | control | 1 | 16.2 | 4.77 | 7 | 1 |
| CRAZI | control | 2 | 28.32 | 9.34 | 7 | 1 |
| HUSKI | control | 1 | 18.17 | 5.09 | 7 | 1 |
| HUSKI | control | 2 | 21.5 | 7 | 7 | 1 |
| LAKTI | control | 1 | 22.88 | 11.57 | 7 | 1 |
| LAKTI | control | 2 | 22.72 | 8.18 | 7 | 1 |
| ALGANS | control | 1 | 21.69 | 10.98 | 7 | 1 |
| ALGANS | control | 2 | 14.15 | 5.26 | 7 | 1 |
| PICKER | control | 1 | 30.66 | 8.62 | 7 | 1 |
| PICKER | control | 2 | 33.27 | 9.43 | 7 | 1 |
| FERGUS | control | 1 | 23.9 | 7.53 | 7 | 1 |
| FERGUS | control | 2 | 38.67 | 14.45 | 7 | 1 |
| CODISCO | control | 1 | 15.24 | 3.21 | 7 | 1 |
| CODISCO | control | 2 | 24.4 | 8.07 | 7 | 1 |
| CODIFAR | control | 1 | 25.52 | 5.96 | 7 | 1 |
| CODIFAR | control | 2 | 27.71 | 7.14 | 7 | 1 |
| CLARITI | control | 1 | 26.95 | 7.35 | 7 | 1 |
| CLARITI | control | 2 | 27.95 | 11.27 | 7 | 1 |
| JUSTINA | control | 1 | 12.59 | 3.78 | 7 | 1 |
| JUSTINA | control | 2 | 19.76 | 6.57 | 7 | 1 |
| PR29B29 | control | 1 | 22.8 | 6.32 | 7 | 1 |
| PR29B29 | control | 2 | 14.11 | 3.91 | 7 | 1 |
| P329D60 | control | 1 | 27.29 | 7.04 | 7 | 1 |
| P329D60 | control | 2 | 29.86 | 8.1 | 7 | 1 |
| CRAZI | cold | 1 | 11.4 | 4.31 | 7 | 1 |
| CRAZI | cold | 2 | 10.39 | 3.31 | 7 | 1 |
| HUSKI | cold | 1 | 6.92 | 0.66 | 7 | 1 |
| HUSKI | cold | 2 | 7.55 | 0 | 7 | 1 |
| LAKTI | cold | 1 | 12.39 | 2.4 | 7 | 1 |
| LAKTI | cold | 2 | 10.92 | 0.78 | 7 | 1 |
| ALGANS | cold | 1 | 2.72 | 1.3 | 7 | 1 |
| ALGANS | cold | 2 | 2.72 | 1.93 | 7 | 1 |
| PICKER | cold | 1 | 15.97 | 3.26 | 7 | 1 |
| PICKER | cold | 2 | 17.75 | 3.99 | 7 | 1 |
| FERGUS | cold | 1 | 6.23 | 1.14 | 7 | 1 |
| FERGUS | cold | 2 | 9.7 | 1.73 | 7 | 1 |
| CODISCO | cold | 1 | 5.26 | 0.57 | 7 | 1 |
| CODISCO | cold | 2 | 4.54 | 1.56 | 7 | 1 |
| CODIFAR | cold | 1 | 18.87 | 4.29 | 7 | 1 |
| CODIFAR | cold | 2 | 14.51 | 3.35 | 7 | 1 |
| CLARITI | cold | 1 | 8.36 | 3.26 | 7 | 1 |
| CLARITI | cold | 2 | 9.23 | 2.23 | 7 | 1 |
| JUSTINA | cold | 1 | 3.26 | 1.06 | 7 | 1 |
| JUSTINA | cold | 2 | 2.16 | 0.8 | 7 | 1 |
| PR29B29 | cold | 1 | 8.2 | 2.1 | 7 | 1 |
| PR29B29 | cold | 2 | 9.67 | 2.84 | 7 | 1 |
| P329D60 | cold | 1 | 10.98 | 2.52 | 7 | 1 |
| P329D60 | cold | 2 | 8.82 | 2.22 | 7 | 1 |
| CRAZI | control | 1 | 19.91 | 6.26 | 8 | 1 |
| CRAZI | control | 2 | 34.21 | 12.65 | 8 | 1 |
| HUSKI | control | 1 | 24.9 | 7.54 | 8 | 1 |
| HUSKI | control | 2 | 25.91 | 9.65 | 8 | 1 |
| LAKTI | control | 1 | 27.37 | 14.49 | 8 | 1 |
| LAKTI | control | 2 | 27.93 | 10.54 | 8 | 1 |
| ALGANS | control | 1 | 25.91 | 14.11 | 8 | 1 |
| ALGANS | control | 2 | 18.57 | 7.21 | 8 | 1 |
| PICKER | control | 1 | 35.06 | 10.59 | 8 | 1 |
| PICKER | control | 2 | 40.57 | 11.48 | 8 | 1 |
| FERGUS | control | 1 | 29.05 | 9.63 | 8 | 1 |
| FERGUS | control | 2 | 45.98 | 18.21 | 8 | 1 |
| CODISCO | control | 1 | 18.5 | 4.49 | 8 | 1 |
| CODISCO | control | 2 | 30.17 | 9.41 | 8 | 1 |
| CODIFAR | control | 1 | 32.92 | 7.48 | 8 | 1 |
| CODIFAR | control | 2 | 36.25 | 10.51 | 8 | 1 |
| CLARITI | control | 1 | 32.67 | 10.74 | 8 | 1 |
| CLARITI | control | 2 | 30.57 | 11.64 | 8 | 1 |
| JUSTINA | control | 1 | 15.73 | 5.94 | 8 | 1 |
| JUSTINA | control | 2 | 24.26 | 9.99 | 8 | 1 |
| PR29B29 | control | 1 | 28.16 | 9.3 | 8 | 1 |
| PR29B29 | control | 2 | 19.46 | 5.88 | 8 | 1 |
| P329D60 | control | 1 | 35.65 | 10.45 | 8 | 1 |
| P329D60 | control | 2 | 36.13 | 10.83 | 8 | 1 |
| CRAZI | cold | 1 | 13.23 | 4.77 | 8 | 1 |
| CRAZI | cold | 2 | 11.75 | 3.91 | 8 | 1 |
| HUSKI | cold | 1 | 7.59 | 1.76 | 8 | 1 |
| HUSKI | cold | 2 | 8.63 | 2 | 8 | 1 |
| LAKTI | cold | 1 | 15.06 | 4.1 | 8 | 1 |
| LAKTI | cold | 2 | 12.54 | 2.43 | 8 | 1 |
| ALGANS | cold | 1 | 4 | 2.22 | 8 | 1 |
| ALGANS | cold | 2 | 3.78 | 2.87 | 8 | 1 |
| PICKER | cold | 1 | 17.39 | 3.69 | 8 | 1 |
| PICKER | cold | 2 | 19.46 | 4.32 | 8 | 1 |
| FERGUS | cold | 1 | 8.29 | 2.73 | 8 | 1 |
| FERGUS | cold | 2 | 12.22 | 3.77 | 8 | 1 |
| CODISCO | cold | 1 | 6.59 | 1.72 | 8 | 1 |
| CODISCO | cold | 2 | 5.73 | 2.3 | 8 | 1 |
| CODIFAR | cold | 1 | 22.54 | 4.98 | 8 | 1 |
| CODIFAR | cold | 2 | 17.2 | 4.3 | 8 | 1 |
| CLARITI | cold | 1 | 9.77 | 3.63 | 8 | 1 |
| CLARITI | cold | 2 | 10.87 | 3.16 | 8 | 1 |
| JUSTINA | cold | 1 | 4.11 | 0.88 | 8 | 1 |
| JUSTINA | cold | 2 | 3.18 | 1.15 | 8 | 1 |
| PR29B29 | cold | 1 | 10.54 | 3.45 | 8 | 1 |
| PR29B29 | cold | 2 | 12.89 | 4.14 | 8 | 1 |
| P329D60 | cold | 1 | 14.26 | 3.54 | 8 | 1 |
| P329D60 | cold | 2 | 10.41 | 2.14 | 8 | 1 |
| CRAZI | control | 1 | 0.67 | 0.58 | 1 | 2 |
| CRAZI | control | 2 | 0.77 | 0.67 | 1 | 2 |
| HUSKI | control | 1 | 1.14 | 0.11 | 1 | 2 |
| HUSKI | control | 2 | 1.66 | 0.24 | 1 | 2 |
| LAKTI | control | 1 | 0.07 | 0 | 1 | 2 |
| LAKTI | control | 2 | 1.2 | 0.13 | 1 | 2 |
| ALGANS | control | 1 | 0.07 | 0 | 1 | 2 |
| ALGANS | control | 2 | 0.13 | 0.15 | 1 | 2 |
| PICKER | control | 1 | 0.78 | 0.18 | 1 | 2 |
| PICKER | control | 2 | 1.42 | 0 | 1 | 2 |
| FERGUS | control | 1 | 2.27 | 0.14 | 1 | 2 |
| FERGUS | control | 2 | 1.43 | 0.06 | 1 | 2 |
| CODISCO | control | 1 | 0.59 | 0 | 1 | 2 |
| CODISCO | control | 2 | 0.71 | 0.14 | 1 | 2 |
| CODIFAR | control | 1 | 0.68 | 0 | 1 | 2 |
| CODIFAR | control | 2 | 0.79 | 0 | 1 | 2 |
| CLARITI | control | 1 | 0.66 | 0.09 | 1 | 2 |
| CLARITI | control | 2 | 0.48 | 0 | 1 | 2 |
| JUSTINA | control | 1 | 1.37 | 0.09 | 1 | 2 |
| JUSTINA | control | 2 | 0.25 | 0 | 1 | 2 |
| PR29B29 | control | 1 | 0.41 | 0 | 1 | 2 |
| PR29B29 | control | 2 | 0.21 | 0 | 1 | 2 |
| P329D60 | control | 1 | 2.99 | 0 | 1 | 2 |
| P329D60 | control | 2 | 1.61 | 0 | 1 | 2 |
| CRAZI | cold | 1 | 1.56 | 1.21 | 1 | 2 |
| CRAZI | cold | 2 | 1.06 | 1.27 | 1 | 2 |
| HUSKI | cold | 1 | 0.5 | 0.13 | 1 | 2 |
| HUSKI | cold | 2 | 0.85 | 0 | 1 | 2 |
| LAKTI | cold | 1 | 0.13 | 0 | 1 | 2 |
| LAKTI | cold | 2 | 0.41 | 0 | 1 | 2 |
| ALGANS | cold | 1 | 0.05 | 0 | 1 | 2 |
| ALGANS | cold | 2 | 0.17 | 0.08 | 1 | 2 |
| PICKER | cold | 1 | 2.18 | 0.23 | 1 | 2 |
| PICKER | cold | 2 | 0.65 | 0 | 1 | 2 |
| FERGUS | cold | 1 | 0.27 | 0 | 1 | 2 |
| FERGUS | cold | 2 | 0.18 | 0 | 1 | 2 |
| CODISCO | cold | 1 | 0.11 | 0 | 1 | 2 |
| CODISCO | cold | 2 | 0.33 | 0.11 | 1 | 2 |
| CODIFAR | cold | 1 | 1.71 | 0.14 | 1 | 2 |
| CODIFAR | cold | 2 | 0.6 | 0.08 | 1 | 2 |
| CLARITI | cold | 1 | 0.56 | 0.13 | 1 | 2 |
| CLARITI | cold | 2 | 0.54 | 0.14 | 1 | 2 |
| JUSTINA | cold | 1 | 0.13 | 0 | 1 | 2 |
| JUSTINA | cold | 2 | 0.21 | 0 | 1 | 2 |
| PR29B29 | cold | 1 | 0.54 | 0.3 | 1 | 2 |
| PR29B29 | cold | 2 | 0.36 | 0.13 | 1 | 2 |
| P329D60 | cold | 1 | 1.28 | 0 | 1 | 2 |
| P329D60 | cold | 2 | 0.33 | 0 | 1 | 2 |
| CRAZI | control | 1 | 4.35 | 1.88 | 2 | 2 |
| CRAZI | control | 2 | 4.42 | 3.03 | 2 | 2 |
| HUSKI | control | 1 | 3.67 | 1.17 | 2 | 2 |
| HUSKI | control | 2 | 3.62 | 1.09 | 2 | 2 |
| LAKTI | control | 1 | 3.66 | 0.44 | 2 | 2 |
| LAKTI | control | 2 | 3.26 | 0.88 | 2 | 2 |
| ALGANS | control | 1 | 1.34 | 0.85 | 2 | 2 |
| ALGANS | control | 2 | 1.49 | 0.68 | 2 | 2 |
| PICKER | control | 1 | 6.02 | 1.31 | 2 | 2 |
| PICKER | control | 2 | 6.88 | 1.32 | 2 | 2 |
| FERGUS | control | 1 | 8.57 | 0.88 | 2 | 2 |
| FERGUS | control | 2 | 5.73 | 1.21 | 2 | 2 |
| CODISCO | control | 1 | 3.82 | 0.76 | 2 | 2 |
| CODISCO | control | 2 | 3.72 | 1.07 | 2 | 2 |
| CODIFAR | control | 1 | 2.93 | 0 | 2 | 2 |
| CODIFAR | control | 2 | 3.36 | 0.22 | 2 | 2 |
| CLARITI | control | 1 | 3.57 | 1.09 | 2 | 2 |
| CLARITI | control | 2 | 4.12 | 1.62 | 2 | 2 |
| JUSTINA | control | 1 | 3.58 | 0 | 2 | 2 |
| JUSTINA | control | 2 | 0.8 | 0 | 2 | 2 |
| PR29B29 | control | 1 | 1.04 | 0.75 | 2 | 2 |
| PR29B29 | control | 2 | 1.18 | 1.18 | 2 | 2 |
| P329D60 | control | 1 | 6.47 | 0.12 | 2 | 2 |
| P329D60 | control | 2 | 4.35 | 0 | 2 | 2 |
| CRAZI | cold | 1 | 2.06 | 1.28 | 2 | 2 |
| CRAZI | cold | 2 | 1.33 | 1.34 | 2 | 2 |
| HUSKI | cold | 1 | 0.67 | 0.13 | 2 | 2 |
| HUSKI | cold | 2 | 1.06 | 0 | 2 | 2 |
| LAKTI | cold | 1 | 0.17 | 0 | 2 | 2 |
| LAKTI | cold | 2 | 0.64 | 0 | 2 | 2 |
| ALGANS | cold | 1 | 0.36 | 0 | 2 | 2 |
| ALGANS | cold | 2 | 0.27 | 0 | 2 | 2 |
| PICKER | cold | 1 | 3.34 | 0.25 | 2 | 2 |
| PICKER | cold | 2 | 1.11 | 0 | 2 | 2 |
| FERGUS | cold | 1 | 0.35 | 0 | 2 | 2 |
| FERGUS | cold | 2 | 0.24 | 0 | 2 | 2 |
| CODISCO | cold | 1 | 0.07 | 0 | 2 | 2 |
| CODISCO | cold | 2 | 0.41 | 0.13 | 2 | 2 |
| CODIFAR | cold | 1 | 2.14 | 0.14 | 2 | 2 |
| CODIFAR | cold | 2 | 0.94 | 0.12 | 2 | 2 |
| CLARITI | cold | 1 | 0.73 | 0 | 2 | 2 |
| CLARITI | cold | 2 | 0.7 | 0.15 | 2 | 2 |
| JUSTINA | cold | 1 | 0.2 | 0 | 2 | 2 |
| JUSTINA | cold | 2 | 0.31 | 0 | 2 | 2 |
| PR29B29 | cold | 1 | 1.18 | 0.57 | 2 | 2 |
| PR29B29 | cold | 2 | 0.63 | 0.27 | 2 | 2 |
| P329D60 | cold | 1 | 2.47 | 0 | 2 | 2 |
| P329D60 | cold | 2 | 1.24 | 0 | 2 | 2 |
| CRAZI | control | 1 | 8.91 | 3.26 | 3 | 2 |
| CRAZI | control | 2 | 8.64 | 3.58 | 3 | 2 |
| HUSKI | control | 1 | 6.2 | 2.23 | 3 | 2 |
| HUSKI | control | 2 | 5.58 | 1.95 | 3 | 2 |
| LAKTI | control | 1 | 4.36 | 1.44 | 3 | 2 |
| LAKTI | control | 2 | 5.55 | 2.66 | 3 | 2 |
| ALGANS | control | 1 | 5.3 | 2.45 | 3 | 2 |
| ALGANS | control | 2 | 4.29 | 1.99 | 3 | 2 |
| PICKER | control | 1 | 10.99 | 2.02 | 3 | 2 |
| PICKER | control | 2 | 13.02 | 2.79 | 3 | 2 |
| FERGUS | control | 1 | 11.72 | 2.44 | 3 | 2 |
| FERGUS | control | 2 | 10.53 | 1.98 | 3 | 2 |
| CODISCO | control | 1 | 9.27 | 2.51 | 3 | 2 |
| CODISCO | control | 2 | 9.68 | 2.86 | 3 | 2 |
| CODIFAR | control | 1 | 5.17 | 0 | 3 | 2 |
| CODIFAR | control | 2 | 5.94 | 0.47 | 3 | 2 |
| CLARITI | control | 1 | 6.42 | 1.62 | 3 | 2 |
| CLARITI | control | 2 | 11.1 | 3.18 | 3 | 2 |
| JUSTINA | control | 1 | 5.79 | 2.68 | 3 | 2 |
| JUSTINA | control | 2 | 1.35 | 0.13 | 3 | 2 |
| PR29B29 | control | 1 | 1.66 | 2.09 | 3 | 2 |
| PR29B29 | control | 2 | 2.16 | 2.47 | 3 | 2 |
| P329D60 | control | 1 | 9.94 | 2.06 | 3 | 2 |
| P329D60 | control | 2 | 7.08 | 1.22 | 3 | 2 |
| CRAZI | cold | 1 | 2.75 | 1.96 | 3 | 2 |
| CRAZI | cold | 2 | 2 | 1.94 | 3 | 2 |
| HUSKI | cold | 1 | 1.66 | 0.94 | 3 | 2 |
| HUSKI | cold | 2 | 1.57 | 0.67 | 3 | 2 |
| LAKTI | cold | 1 | 0.43 | 0.11 | 3 | 2 |
| LAKTI | cold | 2 | 1.6 | 0.12 | 3 | 2 |
| ALGANS | cold | 1 | 0.66 | 0.34 | 3 | 2 |
| ALGANS | cold | 2 | 0.37 | 0.33 | 3 | 2 |
| PICKER | cold | 1 | 4.78 | 1.26 | 3 | 2 |
| PICKER | cold | 2 | 2.53 | 0.59 | 3 | 2 |
| FERGUS | cold | 1 | 0.52 | 0 | 3 | 2 |
| FERGUS | cold | 2 | 0.39 | 0 | 3 | 2 |
| CODISCO | cold | 1 | 0.67 | 0.2 | 3 | 2 |
| CODISCO | cold | 2 | 0.83 | 0.26 | 3 | 2 |
| CODIFAR | cold | 1 | 2.87 | 0.3 | 3 | 2 |
| CODIFAR | cold | 2 | 1.71 | 0.51 | 3 | 2 |
| CLARITI | cold | 1 | 1.43 | 0.13 | 3 | 2 |
| CLARITI | cold | 2 | 1.15 | 0.47 | 3 | 2 |
| JUSTINA | cold | 1 | 0.23 | 0 | 3 | 2 |
| JUSTINA | cold | 2 | 0.83 | 0.5 | 3 | 2 |
| PR29B29 | cold | 1 | 1.81 | 0.84 | 3 | 2 |
| PR29B29 | cold | 2 | 0.89 | 0.4 | 3 | 2 |
| P329D60 | cold | 1 | 3.67 | 0.14 | 3 | 2 |
| P329D60 | cold | 2 | 2.15 | 0.16 | 3 | 2 |
| CRAZI | control | 1 | 13.47 | 4.63 | 4 | 2 |
| CRAZI | control | 2 | 12.87 | 4.13 | 4 | 2 |
| HUSKI | control | 1 | 9.95 | 3.12 | 4 | 2 |
| HUSKI | control | 2 | 9.65 | 3.04 | 4 | 2 |
| LAKTI | control | 1 | 8.64 | 2.44 | 4 | 2 |
| LAKTI | control | 2 | 9.89 | 4.44 | 4 | 2 |
| ALGANS | control | 1 | 9.25 | 4.05 | 4 | 2 |
| ALGANS | control | 2 | 7.1 | 3.31 | 4 | 2 |
| PICKER | control | 1 | 15.95 | 2.74 | 4 | 2 |
| PICKER | control | 2 | 19.16 | 4.27 | 4 | 2 |
| FERGUS | control | 1 | 14.87 | 4.74 | 4 | 2 |
| FERGUS | control | 2 | 14.37 | 6.11 | 4 | 2 |
| CODISCO | control | 1 | 12 | 3.42 | 4 | 2 |
| CODISCO | control | 2 | 12.65 | 3.76 | 4 | 2 |
| CODIFAR | control | 1 | 9 | 0.96 | 4 | 2 |
| CODIFAR | control | 2 | 11.19 | 1.53 | 4 | 2 |
| CLARITI | control | 1 | 9.27 | 2.16 | 4 | 2 |
| CLARITI | control | 2 | 18.08 | 4.74 | 4 | 2 |
| JUSTINA | control | 1 | 11.57 | 4.07 | 4 | 2 |
| JUSTINA | control | 2 | 2.71 | 0.76 | 4 | 2 |
| PR29B29 | control | 1 | 4.46 | 3.43 | 4 | 2 |
| PR29B29 | control | 2 | 5.08 | 3.76 | 4 | 2 |
| P329D60 | control | 1 | 16.89 | 3.99 | 4 | 2 |
| P329D60 | control | 2 | 12.54 | 2.44 | 4 | 2 |
| CRAZI | cold | 1 | 3.32 | 2.02 | 4 | 2 |
| CRAZI | cold | 2 | 2.28 | 1.85 | 4 | 2 |
| HUSKI | cold | 1 | 2.39 | 1.03 | 4 | 2 |
| HUSKI | cold | 2 | 1.94 | 0.67 | 4 | 2 |
| LAKTI | cold | 1 | 0.9 | 0.17 | 4 | 2 |
| LAKTI | cold | 2 | 3.24 | 0.31 | 4 | 2 |
| ALGANS | cold | 1 | 0.94 | 0.42 | 4 | 2 |
| ALGANS | cold | 2 | 0.41 | 0.4 | 4 | 2 |
| PICKER | cold | 1 | 5.5 | 1.33 | 4 | 2 |
| PICKER | cold | 2 | 3.16 | 0.65 | 4 | 2 |
| FERGUS | cold | 1 | 0.62 | 0 | 4 | 2 |
| FERGUS | cold | 2 | 0.51 | 0 | 4 | 2 |
| CODISCO | cold | 1 | 0.99 | 0.21 | 4 | 2 |
| CODISCO | cold | 2 | 0.67 | 0.29 | 4 | 2 |
| CODIFAR | cold | 1 | 4.23 | 0.3 | 4 | 2 |
| CODIFAR | cold | 2 | 3.33 | 0.58 | 4 | 2 |
| CLARITI | cold | 1 | 2.07 | 0.5 | 4 | 2 |
| CLARITI | cold | 2 | 2.06 | 0.95 | 4 | 2 |
| JUSTINA | cold | 1 | 0.33 | 0 | 4 | 2 |
| JUSTINA | cold | 2 | 0.96 | 0.52 | 4 | 2 |
| PR29B29 | cold | 1 | 2.7 | 1.3 | 4 | 2 |
| PR29B29 | cold | 2 | 1.34 | 0.63 | 4 | 2 |
| P329D60 | cold | 1 | 4.12 | 0.23 | 4 | 2 |
| P329D60 | cold | 2 | 2.56 | 0.19 | 4 | 2 |
| CRAZI | control | 1 | 18.8 | 6.71 | 5 | 2 |
| CRAZI | control | 2 | 18.03 | 5.63 | 5 | 2 |
| HUSKI | control | 1 | 13.7 | 4.01 | 5 | 2 |
| HUSKI | control | 2 | 13.72 | 4.13 | 5 | 2 |
| LAKTI | control | 1 | 13.63 | 4.18 | 5 | 2 |
| LAKTI | control | 2 | 16.52 | 8.04 | 5 | 2 |
| ALGANS | control | 1 | 12.89 | 5.74 | 5 | 2 |
| ALGANS | control | 2 | 9.82 | 3.95 | 5 | 2 |
| PICKER | control | 1 | 21.63 | 4.06 | 5 | 2 |
| PICKER | control | 2 | 24.61 | 5.33 | 5 | 2 |
| FERGUS | control | 1 | 21.88 | 6.88 | 5 | 2 |
| FERGUS | control | 2 | 19.86 | 8.38 | 5 | 2 |
| CODISCO | control | 1 | 14.72 | 4.32 | 5 | 2 |
| CODISCO | control | 2 | 15.63 | 4.65 | 5 | 2 |
| CODIFAR | control | 1 | 12.82 | 2.46 | 5 | 2 |
| CODIFAR | control | 2 | 16.44 | 3.55 | 5 | 2 |
| CLARITI | control | 1 | 12.59 | 2.87 | 5 | 2 |
| CLARITI | control | 2 | 23.84 | 6.35 | 5 | 2 |
| JUSTINA | control | 1 | 14.09 | 5.45 | 5 | 2 |
| JUSTINA | control | 2 | 4.92 | 1.39 | 5 | 2 |
| PR29B29 | control | 1 | 7.26 | 5.36 | 5 | 2 |
| PR29B29 | control | 2 | 8.04 | 4.04 | 5 | 2 |
| P329D60 | control | 1 | 24.82 | 6.18 | 5 | 2 |
| P329D60 | control | 2 | 17.74 | 4 | 5 | 2 |
| CRAZI | cold | 1 | 4.04 | 2.31 | 5 | 2 |
| CRAZI | cold | 2 | 2.83 | 1.88 | 5 | 2 |
| HUSKI | cold | 1 | 3.2 | 1.51 | 5 | 2 |
| HUSKI | cold | 2 | 2.76 | 0.79 | 5 | 2 |
| LAKTI | cold | 1 | 1.37 | 0.23 | 5 | 2 |
| LAKTI | cold | 2 | 4.89 | 0.5 | 5 | 2 |
| ALGANS | cold | 1 | 1.22 | 0.49 | 5 | 2 |
| ALGANS | cold | 2 | 0.45 | 0.48 | 5 | 2 |
| PICKER | cold | 1 | 6.21 | 1.59 | 5 | 2 |
| PICKER | cold | 2 | 3.86 | 1.33 | 5 | 2 |
| FERGUS | cold | 1 | 0.97 | 0 | 5 | 2 |
| FERGUS | cold | 2 | 0.79 | 0 | 5 | 2 |
| CODISCO | cold | 1 | 1.72 | 0.67 | 5 | 2 |
| CODISCO | cold | 2 | 1.44 | 0.42 | 5 | 2 |
| CODIFAR | cold | 1 | 5.59 | 1.25 | 5 | 2 |
| CODIFAR | cold | 2 | 5.35 | 2.2 | 5 | 2 |
| CLARITI | cold | 1 | 2.72 | 0.87 | 5 | 2 |
| CLARITI | cold | 2 | 2.97 | 1.42 | 5 | 2 |
| JUSTINA | cold | 1 | 0.52 | 0 | 5 | 2 |
| JUSTINA | cold | 2 | 1.1 | 0.54 | 5 | 2 |
| PR29B29 | cold | 1 | 3.59 | 1.76 | 5 | 2 |
| PR29B29 | cold | 2 | 1.78 | 0.87 | 5 | 2 |
| P329D60 | cold | 1 | 4.57 | 0.37 | 5 | 2 |
| P329D60 | cold | 2 | 2.96 | 0.29 | 5 | 2 |
| CRAZI | control | 1 | 24.13 | 8.78 | 6 | 2 |
| CRAZI | control | 2 | 23.19 | 7.12 | 6 | 2 |
| HUSKI | control | 1 | 16.48 | 5.13 | 6 | 2 |
| HUSKI | control | 2 | 16.3 | 5.47 | 6 | 2 |
| LAKTI | control | 1 | 15.91 | 5.91 | 6 | 2 |
| LAKTI | control | 2 | 19.88 | 11.64 | 6 | 2 |
| ALGANS | control | 1 | 16.53 | 7.43 | 6 | 2 |
| ALGANS | control | 2 | 12.55 | 4.69 | 6 | 2 |
| PICKER | control | 1 | 27.31 | 5.38 | 6 | 2 |
| PICKER | control | 2 | 30.07 | 6.39 | 6 | 2 |
| FERGUS | control | 1 | 25.38 | 9.03 | 6 | 2 |
| FERGUS | control | 2 | 24.36 | 10.7 | 6 | 2 |
| CODISCO | control | 1 | 18.32 | 5.41 | 6 | 2 |
| CODISCO | control | 2 | 19.23 | 5.71 | 6 | 2 |
| CODIFAR | control | 1 | 16.64 | 3.95 | 6 | 2 |
| CODIFAR | control | 2 | 21.69 | 5.57 | 6 | 2 |
| CLARITI | control | 1 | 15.9 | 3.59 | 6 | 2 |
| CLARITI | control | 2 | 29.61 | 7.96 | 6 | 2 |
| JUSTINA | control | 1 | 16.61 | 7.69 | 6 | 2 |
| JUSTINA | control | 2 | 7.14 | 1.91 | 6 | 2 |
| PR29B29 | control | 1 | 12.85 | 7.29 | 6 | 2 |
| PR29B29 | control | 2 | 13.93 | 4.32 | 6 | 2 |
| P329D60 | control | 1 | 32.74 | 8.36 | 6 | 2 |
| P329D60 | control | 2 | 22.94 | 5.55 | 6 | 2 |
| CRAZI | cold | 1 | 4.59 | 2.5 | 6 | 2 |
| CRAZI | cold | 2 | 3.31 | 2.16 | 6 | 2 |
| HUSKI | cold | 1 | 3.54 | 1.72 | 6 | 2 |
| HUSKI | cold | 2 | 3.59 | 1.2 | 6 | 2 |
| LAKTI | cold | 1 | 2.53 | 0.38 | 6 | 2 |
| LAKTI | cold | 2 | 5.68 | 0.87 | 6 | 2 |
| ALGANS | cold | 1 | 1.79 | 0.65 | 6 | 2 |
| ALGANS | cold | 2 | 0.53 | 0.62 | 6 | 2 |
| PICKER | cold | 1 | 7.06 | 1.94 | 6 | 2 |
| PICKER | cold | 2 | 4.45 | 1.53 | 6 | 2 |
| FERGUS | cold | 1 | 1.03 | 0.06 | 6 | 2 |
| FERGUS | cold | 2 | 0.85 | 0.04 | 6 | 2 |
| CODISCO | cold | 1 | 2.1 | 0.75 | 6 | 2 |
| CODISCO | cold | 2 | 1.73 | 0.48 | 6 | 2 |
| CODIFAR | cold | 1 | 6.11 | 1.51 | 6 | 2 |
| CODIFAR | cold | 2 | 5.96 | 2.33 | 6 | 2 |
| CLARITI | cold | 1 | 3.22 | 1.12 | 6 | 2 |
| CLARITI | cold | 2 | 3.7 | 1.7 | 6 | 2 |
| JUSTINA | cold | 1 | 0.81 | 0 | 6 | 2 |
| JUSTINA | cold | 2 | 1.37 | 0.58 | 6 | 2 |
| PR29B29 | cold | 1 | 5.38 | 2.68 | 6 | 2 |
| PR29B29 | cold | 2 | 2.67 | 1.34 | 6 | 2 |
| P329D60 | cold | 1 | 5.47 | 0.6 | 6 | 2 |
| P329D60 | cold | 2 | 3.77 | 0.42 | 6 | 2 |
| CRAZI | control | 1 | 28.05 | 11.18 | 7 | 2 |
| CRAZI | control | 2 | 27.49 | 9.25 | 7 | 2 |
| HUSKI | control | 1 | 19.25 | 6.24 | 7 | 2 |
| HUSKI | control | 2 | 18.89 | 6.8 | 7 | 2 |
| LAKTI | control | 1 | 18.2 | 8.61 | 7 | 2 |
| LAKTI | control | 2 | 23.25 | 16.3 | 7 | 2 |
| ALGANS | control | 1 | 20.17 | 9.12 | 7 | 2 |
| ALGANS | control | 2 | 15.28 | 5.37 | 7 | 2 |
| PICKER | control | 1 | 31.39 | 7.52 | 7 | 2 |
| PICKER | control | 2 | 32.81 | 7.25 | 7 | 2 |
| FERGUS | control | 1 | 28.88 | 14.13 | 7 | 2 |
| FERGUS | control | 2 | 29.12 | 12.88 | 7 | 2 |
| CODISCO | control | 1 | 21.92 | 6.48 | 7 | 2 |
| CODISCO | control | 2 | 22.82 | 6.76 | 7 | 2 |
| CODIFAR | control | 1 | 20.47 | 6.51 | 7 | 2 |
| CODIFAR | control | 2 | 26.94 | 7.49 | 7 | 2 |
| CLARITI | control | 1 | 20.81 | 5.28 | 7 | 2 |
| CLARITI | control | 2 | 36.54 | 10.41 | 7 | 2 |
| JUSTINA | control | 1 | 21.64 | 9.92 | 7 | 2 |
| JUSTINA | control | 2 | 11.57 | 2.44 | 7 | 2 |
| PR29B29 | control | 1 | 21.87 | 9.73 | 7 | 2 |
| PR29B29 | control | 2 | 19.6 | 6.42 | 7 | 2 |
| P329D60 | control | 1 | 41.1 | 11.96 | 7 | 2 |
| P329D60 | control | 2 | 25.69 | 7.04 | 7 | 2 |
| CRAZI | cold | 1 | 5.14 | 2.7 | 7 | 2 |
| CRAZI | cold | 2 | 3.78 | 2.44 | 7 | 2 |
| HUSKI | cold | 1 | 3.88 | 1.93 | 7 | 2 |
| HUSKI | cold | 2 | 4.43 | 1.61 | 7 | 2 |
| LAKTI | cold | 1 | 3.7 | 0.54 | 7 | 2 |
| LAKTI | cold | 2 | 6.47 | 1.25 | 7 | 2 |
| ALGANS | cold | 1 | 1.89 | 0.91 | 7 | 2 |
| ALGANS | cold | 2 | 0.62 | 0.66 | 7 | 2 |
| PICKER | cold | 1 | 7.9 | 2.3 | 7 | 2 |
| PICKER | cold | 2 | 5.04 | 1.74 | 7 | 2 |
| FERGUS | cold | 1 | 1.44 | 0.12 | 7 | 2 |
| FERGUS | cold | 2 | 1.19 | 0.08 | 7 | 2 |
| CODISCO | cold | 1 | 2.49 | 0.82 | 7 | 2 |
| CODISCO | cold | 2 | 2.02 | 0.54 | 7 | 2 |
| CODIFAR | cold | 1 | 6.64 | 1.78 | 7 | 2 |
| CODIFAR | cold | 2 | 6.57 | 2.47 | 7 | 2 |
| CLARITI | cold | 1 | 3.72 | 1.37 | 7 | 2 |
| CLARITI | cold | 2 | 4.43 | 1.98 | 7 | 2 |
| JUSTINA | cold | 1 | 0.85 | 0.12 | 7 | 2 |
| JUSTINA | cold | 2 | 1.41 | 0.58 | 7 | 2 |
| PR29B29 | cold | 1 | 6.11 | 2.74 | 7 | 2 |
| PR29B29 | cold | 2 | 3.48 | 1.75 | 7 | 2 |
| P329D60 | cold | 1 | 5.67 | 0.67 | 7 | 2 |
| P329D60 | cold | 2 | 4.09 | 0.42 | 7 | 2 |
| CRAZI | control | 1 | 31.98 | 13.57 | 8 | 2 |
| CRAZI | control | 2 | 31.78 | 11.38 | 8 | 2 |
| HUSKI | control | 1 | 24.8 | 8.48 | 8 | 2 |
| HUSKI | control | 2 | 24.06 | 9.48 | 8 | 2 |
| LAKTI | control | 1 | 22.77 | 11.3 | 8 | 2 |
| LAKTI | control | 2 | 29.97 | 20.96 | 8 | 2 |
| ALGANS | control | 1 | 23.81 | 10.81 | 8 | 2 |
| ALGANS | control | 2 | 18.01 | 6.06 | 8 | 2 |
| PICKER | control | 1 | 35.48 | 9.66 | 8 | 2 |
| PICKER | control | 2 | 35.56 | 8.11 | 8 | 2 |
| FERGUS | control | 1 | 38.33 | 19.22 | 8 | 2 |
| FERGUS | control | 2 | 38.32 | 15.65 | 8 | 2 |
| CODISCO | control | 1 | 29.12 | 8.64 | 8 | 2 |
| CODISCO | control | 2 | 30.02 | 8.87 | 8 | 2 |
| CODIFAR | control | 1 | 32.51 | 9.06 | 8 | 2 |
| CODIFAR | control | 2 | 37.54 | 9.4 | 8 | 2 |
| CLARITI | control | 1 | 25.72 | 6.98 | 8 | 2 |
| CLARITI | control | 2 | 43.48 | 12.87 | 8 | 2 |
| JUSTINA | control | 1 | 31.63 | 13.13 | 8 | 2 |
| JUSTINA | control | 2 | 18.71 | 4.7 | 8 | 2 |
| PR29B29 | control | 1 | 30.88 | 12.16 | 8 | 2 |
| PR29B29 | control | 2 | 25.27 | 8.52 | 8 | 2 |
| P329D60 | control | 1 | 49.46 | 15.56 | 8 | 2 |
| P329D60 | control | 2 | 28.45 | 8.52 | 8 | 2 |
| CRAZI | cold | 1 | 6.25 | 3.1 | 8 | 2 |
| CRAZI | cold | 2 | 4.74 | 2.99 | 8 | 2 |
| HUSKI | cold | 1 | 4.56 | 2.35 | 8 | 2 |
| HUSKI | cold | 2 | 6.1 | 2.43 | 8 | 2 |
| LAKTI | cold | 1 | 6.03 | 0.85 | 8 | 2 |
| LAKTI | cold | 2 | 8.05 | 2 | 8 | 2 |
| ALGANS | cold | 1 | 2 | 1.16 | 8 | 2 |
| ALGANS | cold | 2 | 0.72 | 0.7 | 8 | 2 |
| PICKER | cold | 1 | 9.58 | 3.01 | 8 | 2 |
| PICKER | cold | 2 | 6.23 | 2.15 | 8 | 2 |
| FERGUS | cold | 1 | 1.91 | 0.25 | 8 | 2 |
| FERGUS | cold | 2 | 1.59 | 0.16 | 8 | 2 |
| CODISCO | cold | 1 | 3.25 | 0.98 | 8 | 2 |
| CODISCO | cold | 2 | 2.6 | 0.66 | 8 | 2 |
| CODIFAR | cold | 1 | 7.68 | 2.31 | 8 | 2 |
| CODIFAR | cold | 2 | 7.79 | 2.74 | 8 | 2 |
| CLARITI | cold | 1 | 4.72 | 1.87 | 8 | 2 |
| CLARITI | cold | 2 | 5.89 | 2.54 | 8 | 2 |
| JUSTINA | cold | 1 | 0.89 | 0.24 | 8 | 2 |
| JUSTINA | cold | 2 | 1.45 | 0.57 | 8 | 2 |
| PR29B29 | cold | 1 | 6.83 | 2.81 | 8 | 2 |
| PR29B29 | cold | 2 | 4.29 | 2.16 | 8 | 2 |
| P329D60 | cold | 1 | 5.87 | 0.73 | 8 | 2 |
| P329D60 | cold | 2 | 4.41 | 0.43 | 8 | 2 |
| CRAZI | control | 1 | 1.02 | 0.9 | 1 | 3 |
| CRAZI | control | 2 | 0.44 | 0.57 | 1 | 3 |
| HUSKI | control | 1 | 0.76 | 0 | 1 | 3 |
| HUSKI | control | 2 | 0.25 | 0.07 | 1 | 3 |
| LAKTI | control | 1 | 0.06 | 0 | 1 | 3 |
| LAKTI | control | 2 | 0.19 | 0 | 1 | 3 |
| ALGANS | control | 1 | 0.45 | 0.31 | 1 | 3 |
| ALGANS | control | 2 | 1.65 | 0.42 | 1 | 3 |
| PICKER | control | 1 | 0.18 | 0 | 1 | 3 |
| PICKER | control | 2 | 2.87 | 0.94 | 1 | 3 |
| FERGUS | control | 1 | 0.25 | 0 | 1 | 3 |
| FERGUS | control | 2 | 0.23 | 0 | 1 | 3 |
| CODISCO | control | 1 | 1.75 | 0.63 | 1 | 3 |
| CODISCO | control | 2 | 1.93 | 1.11 | 1 | 3 |
| CODIFAR | control | 1 | 0.42 | 0 | 1 | 3 |
| CODIFAR | control | 2 | 0.24 | 0 | 1 | 3 |
| CLARITI | control | 1 | 0 | 0 | 1 | 3 |
| CLARITI | control | 2 | 1.01 | 0.37 | 1 | 3 |
| JUSTINA | control | 1 | 0 | 0 | 1 | 3 |
| JUSTINA | control | 2 | 0.44 | 0.52 | 1 | 3 |
| PR29B29 | control | 1 | 0.16 | 0.07 | 1 | 3 |
| PR29B29 | control | 2 | 0 | 0 | 1 | 3 |
| P329D60 | control | 1 | 1.41 | 0 | 1 | 3 |
| P329D60 | control | 2 | 0.73 | 0 | 1 | 3 |
| CRAZI | cold | 1 | 0.14 | 0 | 1 | 3 |
| CRAZI | cold | 2 | 0 | 0 | 1 | 3 |
| HUSKI | cold | 1 | 0.06 | 0.07 | 1 | 3 |
| HUSKI | cold | 2 | 0 | 0 | 1 | 3 |
| LAKTI | cold | 1 | 0 | 0 | 1 | 3 |
| LAKTI | cold | 2 | 0.27 | 0.12 | 1 | 3 |
| ALGANS | cold | 1 | 0.1 | 0.09 | 1 | 3 |
| ALGANS | cold | 2 | 0.16 | 0 | 1 | 3 |
| PICKER | cold | 1 | 0.14 | 0 | 1 | 3 |
| PICKER | cold | 2 | 0 | 0 | 1 | 3 |
| FERGUS | cold | 1 | 0 | 0 | 1 | 3 |
| FERGUS | cold | 2 | 0.49 | 0.23 | 1 | 3 |
| CODISCO | cold | 1 | 0.14 | 0 | 1 | 3 |
| CODISCO | cold | 2 | 0.75 | 0.34 | 1 | 3 |
| CODIFAR | cold | 1 | 0.16 | 0 | 1 | 3 |
| CODIFAR | cold | 2 | 1.33 | 0 | 1 | 3 |
| CLARITI | cold | 1 | 0.23 | 0 | 1 | 3 |
| CLARITI | cold | 2 | 0.14 | 0.11 | 1 | 3 |
| JUSTINA | cold | 1 | 0.28 | 0 | 1 | 3 |
| JUSTINA | cold | 2 | 0.11 | 0 | 1 | 3 |
| PR29B29 | cold | 1 | 0.1 | 0 | 1 | 3 |
| PR29B29 | cold | 2 | 0.24 | 0 | 1 | 3 |
| P329D60 | cold | 1 | 0.44 | 0 | 1 | 3 |
| P329D60 | cold | 2 | 1.51 | 0 | 1 | 3 |
| CRAZI | control | 1 | 3.31 | 1.23 | 2 | 3 |
| CRAZI | control | 2 | 2.83 | 1.14 | 2 | 3 |
| HUSKI | control | 1 | 2.66 | 0.55 | 2 | 3 |
| HUSKI | control | 2 | 2.43 | 0.68 | 2 | 3 |
| LAKTI | control | 1 | 0.82 | 0 | 2 | 3 |
| LAKTI | control | 2 | 1.22 | 0 | 2 | 3 |
| ALGANS | control | 1 | 1.81 | 0.6 | 2 | 3 |
| ALGANS | control | 2 | 3.36 | 0.54 | 2 | 3 |
| PICKER | control | 1 | 2.15 | 0.07 | 2 | 3 |
| PICKER | control | 2 | 7.86 | 1.88 | 2 | 3 |
| FERGUS | control | 1 | 1.43 | 0.37 | 2 | 3 |
| FERGUS | control | 2 | 1.67 | 0.41 | 2 | 3 |
| CODISCO | control | 1 | 3.75 | 1.09 | 2 | 3 |
| CODISCO | control | 2 | 4.13 | 1.69 | 2 | 3 |
| CODIFAR | control | 1 | 2.95 | 0.93 | 2 | 3 |
| CODIFAR | control | 2 | 2.5 | 0.8 | 2 | 3 |
| CLARITI | control | 1 | 3.19 | 0.78 | 2 | 3 |
| CLARITI | control | 2 | 3.61 | 0.83 | 2 | 3 |
| JUSTINA | control | 1 | 2.59 | 0.18 | 2 | 3 |
| JUSTINA | control | 2 | 2.99 | 0.58 | 2 | 3 |
| PR29B29 | control | 1 | 1.71 | 0.92 | 2 | 3 |
| PR29B29 | control | 2 | 0.62 | 0.16 | 2 | 3 |
| P329D60 | control | 1 | 4.46 | 0.56 | 2 | 3 |
| P329D60 | control | 2 | 4.27 | 0.44 | 2 | 3 |
| CRAZI | cold | 1 | 0.77 | 0.65 | 2 | 3 |
| CRAZI | cold | 2 | 0.3 | 0.38 | 2 | 3 |
| HUSKI | cold | 1 | 1.67 | 0.43 | 2 | 3 |
| HUSKI | cold | 2 | 0.23 | 0.15 | 2 | 3 |
| LAKTI | cold | 1 | 0.08 | 0.02 | 2 | 3 |
| LAKTI | cold | 2 | 1.27 | 0.19 | 2 | 3 |
| ALGANS | cold | 1 | 0.22 | 0 | 2 | 3 |
| ALGANS | cold | 2 | 0.57 | 0 | 2 | 3 |
| PICKER | cold | 1 | 1.36 | 0.09 | 2 | 3 |
| PICKER | cold | 2 | 1.07 | 0.32 | 2 | 3 |
| FERGUS | cold | 1 | 0.12 | 0 | 2 | 3 |
| FERGUS | cold | 2 | 1.12 | 0.12 | 2 | 3 |
| CODISCO | cold | 1 | 0.56 | 0 | 2 | 3 |
| CODISCO | cold | 2 | 0.91 | 0.23 | 2 | 3 |
| CODIFAR | cold | 1 | 0.99 | 0 | 2 | 3 |
| CODIFAR | cold | 2 | 1.55 | 0 | 2 | 3 |
| CLARITI | cold | 1 | 0.81 | 0.02 | 2 | 3 |
| CLARITI | cold | 2 | 0.75 | 0.12 | 2 | 3 |
| JUSTINA | cold | 1 | 0.4 | 0 | 2 | 3 |
| JUSTINA | cold | 2 | 0.19 | 0 | 2 | 3 |
| PR29B29 | cold | 1 | 0.44 | 0.16 | 2 | 3 |
| PR29B29 | cold | 2 | 0.35 | 0.14 | 2 | 3 |
| P329D60 | cold | 1 | 1.41 | 0 | 2 | 3 |
| P329D60 | cold | 2 | 2.95 | 0 | 2 | 3 |
| CRAZI | control | 1 | 5.63 | 1.79 | 3 | 3 |
| CRAZI | control | 2 | 5.21 | 1.79 | 3 | 3 |
| HUSKI | control | 1 | 4.56 | 1.1 | 3 | 3 |
| HUSKI | control | 2 | 4.61 | 1.29 | 3 | 3 |
| LAKTI | control | 1 | 1.57 | 0 | 3 | 3 |
| LAKTI | control | 2 | 2.24 | 0.41 | 3 | 3 |
| ALGANS | control | 1 | 4.54 | 2.22 | 3 | 3 |
| ALGANS | control | 2 | 6.34 | 2.35 | 3 | 3 |
| PICKER | control | 1 | 4.12 | 0.35 | 3 | 3 |
| PICKER | control | 2 | 12.84 | 2.81 | 3 | 3 |
| FERGUS | control | 1 | 2.6 | 0.74 | 3 | 3 |
| FERGUS | control | 2 | 3.1 | 0.81 | 3 | 3 |
| CODISCO | control | 1 | 5.74 | 1.56 | 3 | 3 |
| CODISCO | control | 2 | 6.33 | 2.27 | 3 | 3 |
| CODIFAR | control | 1 | 5.25 | 1.01 | 3 | 3 |
| CODIFAR | control | 2 | 4.67 | 0.78 | 3 | 3 |
| CLARITI | control | 1 | 6.37 | 1.56 | 3 | 3 |
| CLARITI | control | 2 | 6.21 | 1.29 | 3 | 3 |
| JUSTINA | control | 1 | 5.18 | 0.64 | 3 | 3 |
| JUSTINA | control | 2 | 5.54 | 1.03 | 3 | 3 |
| PR29B29 | control | 1 | 7.98 | 2.21 | 3 | 3 |
| PR29B29 | control | 2 | 3.5 | 1.13 | 3 | 3 |
| P329D60 | control | 1 | 7.5 | 1.13 | 3 | 3 |
| P329D60 | control | 2 | 7.8 | 0.87 | 3 | 3 |
| CRAZI | cold | 1 | 1.83 | 1.12 | 3 | 3 |
| CRAZI | cold | 2 | 1.45 | 1.13 | 3 | 3 |
| HUSKI | cold | 1 | 2.52 | 0.95 | 3 | 3 |
| HUSKI | cold | 2 | 1.12 | 0.47 | 3 | 3 |
| LAKTI | cold | 1 | 0.16 | 0.03 | 3 | 3 |
| LAKTI | cold | 2 | 2.27 | 0.27 | 3 | 3 |
| ALGANS | cold | 1 | 0.34 | 0.49 | 3 | 3 |
| ALGANS | cold | 2 | 0.97 | 0.29 | 3 | 3 |
| PICKER | cold | 1 | 2.69 | 0.71 | 3 | 3 |
| PICKER | cold | 2 | 1.92 | 0.58 | 3 | 3 |
| FERGUS | cold | 1 | 0.24 | 0 | 3 | 3 |
| FERGUS | cold | 2 | 1.75 | 0 | 3 | 3 |
| CODISCO | cold | 1 | 1.23 | 0.04 | 3 | 3 |
| CODISCO | cold | 2 | 1.11 | 0.11 | 3 | 3 |
| CODIFAR | cold | 1 | 1.87 | 0.47 | 3 | 3 |
| CODIFAR | cold | 2 | 1.77 | 0.19 | 3 | 3 |
| CLARITI | cold | 1 | 1.39 | 0.27 | 3 | 3 |
| CLARITI | cold | 2 | 1.36 | 0.18 | 3 | 3 |
| JUSTINA | cold | 1 | 0.95 | 0.51 | 3 | 3 |
| JUSTINA | cold | 2 | 0.22 | 0 | 3 | 3 |
| PR29B29 | cold | 1 | 0.78 | 0.32 | 3 | 3 |
| PR29B29 | cold | 2 | 0.46 | 0.29 | 3 | 3 |
| P329D60 | cold | 1 | 2.38 | 0 | 3 | 3 |
| P329D60 | cold | 2 | 4.39 | 0.36 | 3 | 3 |
| CRAZI | control | 1 | 10.29 | 2.68 | 4 | 3 |
| CRAZI | control | 2 | 9.99 | 3.01 | 4 | 3 |
| HUSKI | control | 1 | 10.22 | 2.31 | 4 | 3 |
| HUSKI | control | 2 | 13.56 | 3.42 | 4 | 3 |
| LAKTI | control | 1 | 8.45 | 2.04 | 4 | 3 |
| LAKTI | control | 2 | 8.28 | 2.29 | 4 | 3 |
| ALGANS | control | 1 | 7.23 | 3.23 | 4 | 3 |
| ALGANS | control | 2 | 8.46 | 3.1 | 4 | 3 |
| PICKER | control | 1 | 8.06 | 0.7 | 4 | 3 |
| PICKER | control | 2 | 22.82 | 4.69 | 4 | 3 |
| FERGUS | control | 1 | 10.05 | 2.73 | 4 | 3 |
| FERGUS | control | 2 | 13.82 | 4.42 | 4 | 3 |
| CODISCO | control | 1 | 9.74 | 2.5 | 4 | 3 |
| CODISCO | control | 2 | 10.73 | 3.43 | 4 | 3 |
| CODIFAR | control | 1 | 7.55 | 1.56 | 4 | 3 |
| CODIFAR | control | 2 | 6.85 | 1.41 | 4 | 3 |
| CLARITI | control | 1 | 12.75 | 3.12 | 4 | 3 |
| CLARITI | control | 2 | 11.4 | 2.2 | 4 | 3 |
| JUSTINA | control | 1 | 7 | 0.92 | 4 | 3 |
| JUSTINA | control | 2 | 8.93 | 2.05 | 4 | 3 |
| PR29B29 | control | 1 | 14.25 | 3.5 | 4 | 3 |
| PR29B29 | control | 2 | 6.39 | 2.1 | 4 | 3 |
| P329D60 | control | 1 | 13.6 | 2.25 | 4 | 3 |
| P329D60 | control | 2 | 14.87 | 1.75 | 4 | 3 |
| CRAZI | cold | 1 | 2.88 | 1.59 | 4 | 3 |
| CRAZI | cold | 2 | 2.59 | 1.89 | 4 | 3 |
| HUSKI | cold | 1 | 3.37 | 1.46 | 4 | 3 |
| HUSKI | cold | 2 | 2.08 | 0.79 | 4 | 3 |
| LAKTI | cold | 1 | 0.26 | 0.07 | 4 | 3 |
| LAKTI | cold | 2 | 2.65 | 0.47 | 4 | 3 |
| ALGANS | cold | 1 | 0.42 | 0.56 | 4 | 3 |
| ALGANS | cold | 2 | 1.31 | 0.41 | 4 | 3 |
| PICKER | cold | 1 | 4.03 | 1.32 | 4 | 3 |
| PICKER | cold | 2 | 2.78 | 0.84 | 4 | 3 |
| FERGUS | cold | 1 | 0.3 | 0 | 4 | 3 |
| FERGUS | cold | 2 | 2.19 | 0 | 4 | 3 |
| CODISCO | cold | 1 | 1.4 | 0.08 | 4 | 3 |
| CODISCO | cold | 2 | 1.35 | 0.33 | 4 | 3 |
| CODIFAR | cold | 1 | 3.06 | 0.95 | 4 | 3 |
| CODIFAR | cold | 2 | 2.9 | 0.39 | 4 | 3 |
| CLARITI | cold | 1 | 2.25 | 0.67 | 4 | 3 |
| CLARITI | cold | 2 | 2.19 | 0.55 | 4 | 3 |
| JUSTINA | cold | 1 | 1.16 | 0.53 | 4 | 3 |
| JUSTINA | cold | 2 | 0.29 | 0 | 4 | 3 |
| PR29B29 | cold | 1 | 1.14 | 0.49 | 4 | 3 |
| PR29B29 | cold | 2 | 0.7 | 0.45 | 4 | 3 |
| P329D60 | cold | 1 | 2.89 | 0.03 | 4 | 3 |
| P329D60 | cold | 2 | 4.98 | 0.47 | 4 | 3 |
| CRAZI | control | 1 | 14.18 | 3.81 | 5 | 3 |
| CRAZI | control | 2 | 12.64 | 3.74 | 5 | 3 |
| HUSKI | control | 1 | 15.88 | 3.53 | 5 | 3 |
| HUSKI | control | 2 | 22.52 | 5.54 | 5 | 3 |
| LAKTI | control | 1 | 15.33 | 4.08 | 5 | 3 |
| LAKTI | control | 2 | 14.31 | 4.18 | 5 | 3 |
| ALGANS | control | 1 | 8.1 | 3.57 | 5 | 3 |
| ALGANS | control | 2 | 11.68 | 5.03 | 5 | 3 |
| PICKER | control | 1 | 10.94 | 1.48 | 5 | 3 |
| PICKER | control | 2 | 26.36 | 5.27 | 5 | 3 |
| FERGUS | control | 1 | 17.5 | 4.72 | 5 | 3 |
| FERGUS | control | 2 | 24.54 | 8.04 | 5 | 3 |
| CODISCO | control | 1 | 11.12 | 2.75 | 5 | 3 |
| CODISCO | control | 2 | 13.38 | 4.22 | 5 | 3 |
| CODIFAR | control | 1 | 12.15 | 2.18 | 5 | 3 |
| CODIFAR | control | 2 | 11.19 | 2.02 | 5 | 3 |
| CLARITI | control | 1 | 16.76 | 4.08 | 5 | 3 |
| CLARITI | control | 2 | 13.27 | 2.7 | 5 | 3 |
| JUSTINA | control | 1 | 8.82 | 1.2 | 5 | 3 |
| JUSTINA | control | 2 | 12.33 | 3.06 | 5 | 3 |
| PR29B29 | control | 1 | 16.54 | 3.98 | 5 | 3 |
| PR29B29 | control | 2 | 8.68 | 2.57 | 5 | 3 |
| P329D60 | control | 1 | 16.93 | 3.13 | 5 | 3 |
| P329D60 | control | 2 | 18.91 | 2.99 | 5 | 3 |
| CRAZI | cold | 1 | 3.61 | 1.76 | 5 | 3 |
| CRAZI | cold | 2 | 3.24 | 2.1 | 5 | 3 |
| HUSKI | cold | 1 | 4.62 | 1.89 | 5 | 3 |
| HUSKI | cold | 2 | 2.89 | 1.02 | 5 | 3 |
| LAKTI | cold | 1 | 0.35 | 0.21 | 5 | 3 |
| LAKTI | cold | 2 | 3.04 | 0.53 | 5 | 3 |
| ALGANS | cold | 1 | 0.5 | 0.63 | 5 | 3 |
| ALGANS | cold | 2 | 1.65 | 0.53 | 5 | 3 |
| PICKER | cold | 1 | 4.39 | 1.9 | 5 | 3 |
| PICKER | cold | 2 | 3.11 | 1.21 | 5 | 3 |
| FERGUS | cold | 1 | 0.47 | 0.13 | 5 | 3 |
| FERGUS | cold | 2 | 3.42 | 1.49 | 5 | 3 |
| CODISCO | cold | 1 | 1.76 | 0.18 | 5 | 3 |
| CODISCO | cold | 2 | 1.66 | 0.73 | 5 | 3 |
| CODIFAR | cold | 1 | 3.73 | 1.23 | 5 | 3 |
| CODIFAR | cold | 2 | 3.08 | 0.4 | 5 | 3 |
| CLARITI | cold | 1 | 2.56 | 0.96 | 5 | 3 |
| CLARITI | cold | 2 | 2.44 | 0.72 | 5 | 3 |
| JUSTINA | cold | 1 | 1.37 | 0.55 | 5 | 3 |
| JUSTINA | cold | 2 | 0.46 | 0 | 5 | 3 |
| PR29B29 | cold | 1 | 1.92 | 1.12 | 5 | 3 |
| PR29B29 | cold | 2 | 2.62 | 1.08 | 5 | 3 |
| P329D60 | cold | 1 | 3.39 | 0.19 | 5 | 3 |
| P329D60 | cold | 2 | 5.57 | 0.59 | 5 | 3 |
| CRAZI | control | 1 | 18.08 | 4.93 | 6 | 3 |
| CRAZI | control | 2 | 15.3 | 4.48 | 6 | 3 |
| HUSKI | control | 1 | 16.94 | 5.43 | 6 | 3 |
| HUSKI | control | 2 | 23.92 | 8.63 | 6 | 3 |
| LAKTI | control | 1 | 18.52 | 6.99 | 6 | 3 |
| LAKTI | control | 2 | 18.14 | 7.46 | 6 | 3 |
| ALGANS | control | 1 | 10.16 | 4.51 | 6 | 3 |
| ALGANS | control | 2 | 16.76 | 7.29 | 6 | 3 |
| PICKER | control | 1 | 13.83 | 2.26 | 6 | 3 |
| PICKER | control | 2 | 29.9 | 5.86 | 6 | 3 |
| FERGUS | control | 1 | 22.41 | 7.84 | 6 | 3 |
| FERGUS | control | 2 | 27.46 | 11.99 | 6 | 3 |
| CODISCO | control | 1 | 12.51 | 3 | 6 | 3 |
| CODISCO | control | 2 | 16.03 | 5.06 | 6 | 3 |
| CODIFAR | control | 1 | 17.74 | 5.01 | 6 | 3 |
| CODIFAR | control | 2 | 18.28 | 4.81 | 6 | 3 |
| CLARITI | control | 1 | 20.77 | 5.05 | 6 | 3 |
| CLARITI | control | 2 | 15.13 | 3.19 | 6 | 3 |
| JUSTINA | control | 1 | 12.5 | 2.02 | 6 | 3 |
| JUSTINA | control | 2 | 15.75 | 3.67 | 6 | 3 |
| PR29B29 | control | 1 | 18.83 | 4.46 | 6 | 3 |
| PR29B29 | control | 2 | 10.97 | 3.04 | 6 | 3 |
| P329D60 | control | 1 | 20.26 | 4.02 | 6 | 3 |
| P329D60 | control | 2 | 22.96 | 4.24 | 6 | 3 |
| CRAZI | cold | 1 | 3.85 | 1.81 | 6 | 3 |
| CRAZI | cold | 2 | 3.83 | 2.31 | 6 | 3 |
| HUSKI | cold | 1 | 4.79 | 1.96 | 6 | 3 |
| HUSKI | cold | 2 | 3.56 | 1.3 | 6 | 3 |
| LAKTI | cold | 1 | 0.47 | 0.34 | 6 | 3 |
| LAKTI | cold | 2 | 3.99 | 0.6 | 6 | 3 |
| ALGANS | cold | 1 | 0.67 | 0.77 | 6 | 3 |
| ALGANS | cold | 2 | 2.32 | 0.76 | 6 | 3 |
| PICKER | cold | 1 | 4.82 | 1.86 | 6 | 3 |
| PICKER | cold | 2 | 3.45 | 1.23 | 6 | 3 |
| FERGUS | cold | 1 | 0.7 | 0.1 | 6 | 3 |
| FERGUS | cold | 2 | 3.54 | 1.7 | 6 | 3 |
| CODISCO | cold | 1 | 1.9 | 0.25 | 6 | 3 |
| CODISCO | cold | 2 | 1.69 | 1.11 | 6 | 3 |
| CODIFAR | cold | 1 | 4.4 | 1.51 | 6 | 3 |
| CODIFAR | cold | 2 | 3.26 | 0.4 | 6 | 3 |
| CLARITI | cold | 1 | 2.88 | 1.25 | 6 | 3 |
| CLARITI | cold | 2 | 2.7 | 0.89 | 6 | 3 |
| JUSTINA | cold | 1 | 1.78 | 0.59 | 6 | 3 |
| JUSTINA | cold | 2 | 0.71 | 0 | 6 | 3 |
| PR29B29 | cold | 1 | 2.91 | 1.72 | 6 | 3 |
| PR29B29 | cold | 2 | 3.97 | 1.66 | 6 | 3 |
| P329D60 | cold | 1 | 4.24 | 0.37 | 6 | 3 |
| P329D60 | cold | 2 | 6.33 | 0.82 | 6 | 3 |
| CRAZI | control | 1 | 25.87 | 7.17 | 7 | 3 |
| CRAZI | control | 2 | 20.62 | 5.96 | 7 | 3 |
| HUSKI | control | 1 | 18 | 7.33 | 7 | 3 |
| HUSKI | control | 2 | 25.33 | 11.73 | 7 | 3 |
| LAKTI | control | 1 | 21.7 | 9.9 | 7 | 3 |
| LAKTI | control | 2 | 21.96 | 10.74 | 7 | 3 |
| ALGANS | control | 1 | 13 | 5.55 | 7 | 3 |
| ALGANS | control | 2 | 20.59 | 9.86 | 7 | 3 |
| PICKER | control | 1 | 19.6 | 3.82 | 7 | 3 |
| PICKER | control | 2 | 36.98 | 7.03 | 7 | 3 |
| FERGUS | control | 1 | 27.32 | 10.97 | 7 | 3 |
| FERGUS | control | 2 | 30.38 | 15.94 | 7 | 3 |
| CODISCO | control | 1 | 15.28 | 3.51 | 7 | 3 |
| CODISCO | control | 2 | 21.32 | 6.69 | 7 | 3 |
| CODIFAR | control | 1 | 23.32 | 7.84 | 7 | 3 |
| CODIFAR | control | 2 | 25.36 | 7.6 | 7 | 3 |
| CLARITI | control | 1 | 28.8 | 6.98 | 7 | 3 |
| CLARITI | control | 2 | 18.86 | 4.18 | 7 | 3 |
| JUSTINA | control | 1 | 16.18 | 2.85 | 7 | 3 |
| JUSTINA | control | 2 | 19.18 | 4.29 | 7 | 3 |
| PR29B29 | control | 1 | 23.41 | 5.42 | 7 | 3 |
| PR29B29 | control | 2 | 15.55 | 3.97 | 7 | 3 |
| P329D60 | control | 1 | 26.93 | 5.78 | 7 | 3 |
| P329D60 | control | 2 | 31.05 | 6.73 | 7 | 3 |
| CRAZI | cold | 1 | 4.1 | 1.85 | 7 | 3 |
| CRAZI | cold | 2 | 4.42 | 2.53 | 7 | 3 |
| HUSKI | cold | 1 | 4.96 | 2.02 | 7 | 3 |
| HUSKI | cold | 2 | 4.24 | 1.57 | 7 | 3 |
| LAKTI | cold | 1 | 0.65 | 0.46 | 7 | 3 |
| LAKTI | cold | 2 | 5.5 | 0.66 | 7 | 3 |
| ALGANS | cold | 1 | 0.82 | 0.84 | 7 | 3 |
| ALGANS | cold | 2 | 2.41 | 1.08 | 7 | 3 |
| PICKER | cold | 1 | 5.74 | 1.82 | 7 | 3 |
| PICKER | cold | 2 | 4.11 | 1.26 | 7 | 3 |
| FERGUS | cold | 1 | 0.93 | 0.18 | 7 | 3 |
| FERGUS | cold | 2 | 3.7 | 1.91 | 7 | 3 |
| CODISCO | cold | 1 | 2.04 | 0.32 | 7 | 3 |
| CODISCO | cold | 2 | 1.72 | 1.47 | 7 | 3 |
| CODIFAR | cold | 1 | 5.08 | 1.65 | 7 | 3 |
| CODIFAR | cold | 2 | 3.6 | 0.44 | 7 | 3 |
| CLARITI | cold | 1 | 3.32 | 1.49 | 7 | 3 |
| CLARITI | cold | 2 | 3.09 | 1.04 | 7 | 3 |
| JUSTINA | cold | 1 | 1.86 | 0.74 | 7 | 3 |
| JUSTINA | cold | 2 | 0.73 | 0.07 | 7 | 3 |
| PR29B29 | cold | 1 | 3.45 | 1.91 | 7 | 3 |
| PR29B29 | cold | 2 | 4.71 | 1.85 | 7 | 3 |
| P329D60 | cold | 1 | 4.53 | 0.39 | 7 | 3 |
| P329D60 | cold | 2 | 6.56 | 0.89 | 7 | 3 |
| CRAZI | control | 1 | 31.55 | 8.74 | 8 | 3 |
| CRAZI | control | 2 | 25.15 | 7.26 | 8 | 3 |
| HUSKI | control | 1 | 23.07 | 9.4 | 8 | 3 |
| HUSKI | control | 2 | 32.47 | 15.03 | 8 | 3 |
| LAKTI | control | 1 | 26.15 | 11.93 | 8 | 3 |
| LAKTI | control | 2 | 26.46 | 12.93 | 8 | 3 |
| ALGANS | control | 1 | 17.18 | 7.54 | 8 | 3 |
| ALGANS | control | 2 | 24.21 | 12.62 | 8 | 3 |
| PICKER | control | 1 | 23.06 | 4.5 | 8 | 3 |
| PICKER | control | 2 | 43.5 | 8.27 | 8 | 3 |
| FERGUS | control | 1 | 32.83 | 13.22 | 8 | 3 |
| FERGUS | control | 2 | 36.58 | 19.21 | 8 | 3 |
| CODISCO | control | 1 | 18.87 | 4.33 | 8 | 3 |
| CODISCO | control | 2 | 26.33 | 8.26 | 8 | 3 |
| CODIFAR | control | 1 | 30.29 | 10.74 | 8 | 3 |
| CODIFAR | control | 2 | 32.94 | 10.42 | 8 | 3 |
| CLARITI | control | 1 | 33.1 | 8.02 | 8 | 3 |
| CLARITI | control | 2 | 21.68 | 4.8 | 8 | 3 |
| JUSTINA | control | 1 | 23.54 | 4.5 | 8 | 3 |
| JUSTINA | control | 2 | 26.03 | 5.52 | 8 | 3 |
| PR29B29 | control | 1 | 30.01 | 6.95 | 8 | 3 |
| PR29B29 | control | 2 | 19.93 | 5.09 | 8 | 3 |
| P329D60 | control | 1 | 34.08 | 7.31 | 8 | 3 |
| P329D60 | control | 2 | 39.3 | 8.52 | 8 | 3 |
| CRAZI | cold | 1 | 4.79 | 2.05 | 8 | 3 |
| CRAZI | cold | 2 | 5.48 | 2.8 | 8 | 3 |
| HUSKI | cold | 1 | 5.44 | 2.24 | 8 | 3 |
| HUSKI | cold | 2 | 5.12 | 1.96 | 8 | 3 |
| LAKTI | cold | 1 | 0.83 | 0.73 | 8 | 3 |
| LAKTI | cold | 2 | 7 | 1.07 | 8 | 3 |
| ALGANS | cold | 1 | 0.98 | 0.91 | 8 | 3 |
| ALGANS | cold | 2 | 2.49 | 1.4 | 8 | 3 |
| PICKER | cold | 1 | 7.09 | 2.34 | 8 | 3 |
| PICKER | cold | 2 | 5.07 | 1.61 | 8 | 3 |
| FERGUS | cold | 1 | 1.19 | 0.37 | 8 | 3 |
| FERGUS | cold | 2 | 4.2 | 2.36 | 8 | 3 |
| CODISCO | cold | 1 | 2.65 | 0.38 | 8 | 3 |
| CODISCO | cold | 2 | 2.23 | 1.67 | 8 | 3 |
| CODIFAR | cold | 1 | 5.97 | 1.83 | 8 | 3 |
| CODIFAR | cold | 2 | 4.24 | 0.49 | 8 | 3 |
| CLARITI | cold | 1 | 4.37 | 1.98 | 8 | 3 |
| CLARITI | cold | 2 | 4.06 | 1.39 | 8 | 3 |
| JUSTINA | cold | 1 | 1.95 | 0.88 | 8 | 3 |
| JUSTINA | cold | 2 | 0.78 | 0.15 | 8 | 3 |
| PR29B29 | cold | 1 | 4 | 2.11 | 8 | 3 |
| PR29B29 | cold | 2 | 5.46 | 2.04 | 8 | 3 |
| P329D60 | cold | 1 | 4.94 | 0.41 | 8 | 3 |
| P329D60 | cold | 2 | 6.8 | 0.94 | 8 | 3 |

| *Model Information* | |
| --- | --- |
| *Data Set* | WORK.COLD |
| *Dependent Variable* | logroot |
| *Covariance Structure* | Unstructured |
| *Subject Effect* | REP*NAME*TREAT*EXP |
| *Estimation Method* | REML |
| *Residual Variance Method* | None |
| *Fixed Effects SE Method* | Model-Based |
| *Degrees of Freedom Method* | Between-Within |

| *Class Level Information* | | |
| --- | --- | --- |
| *Class* | *Levels* | *Values* |
| *REP* | 2 | 1 2 |
| *NAME* | 12 | ALGANS CLARITI CODIFAR CODISCO CRAZI FERGUS HUSKI JUSTINA LAKTI P329D60 PICKER PR29B29 |
| *TREAT* | 2 | cold control |
| *EXP* | 3 | 1 2 3 |
| *TIME* | 8 | 1 2 3 4 5 6 7 8 |

| *Dimensions* | |
| --- | --- |
| *Covariance Parameters* | 36 |
| *Columns in X* | 354 |
| *Columns in Z* | 0 |
| *Subjects* | 144 |
| *Max Obs Per Subject* | 8 |

| *Number of Observations* | |
| --- | --- |
| *Number of Observations Read* | 1152 |
| *Number of Observations Used* | 1152 |
| *Number of Observations Not Used* | 0 |

| *Iteration History* | | | |
| --- | --- | --- | --- |
| *Iteration* | *Evaluations* | *-2 Res Log Like* | *Criterion* |
| *0* | 1 | 907.44444779 |  |
| *1* | 2 | -1019.48058925 | 0.00000366 |
| *2* | 1 | -1019.48573183 | 0.00000000 |

| Convergence criteria met. |
| --- |

| *Estimated R Correlation Matrix for REP*NAME*TREAT*EXP 1 ALGANS cold 1* | | | | | | | | |
| --- | --- | --- | --- | --- | --- | --- | --- | --- |
| *Row* | *Col1* | *Col2* | *Col3* | *Col4* | *Col5* | *Col6* | *Col7* | *Col8* |
| *1* | 1.0000 | 0.7398 | 0.5485 | 0.4579 | 0.3828 | 0.3041 | 0.2389 | 0.1973 |
| *2* | 0.7398 | 1.0000 | 0.8807 | 0.7326 | 0.6022 | 0.5117 | 0.4194 | 0.3593 |
| *3* | 0.5485 | 0.8807 | 1.0000 | 0.8680 | 0.7586 | 0.6555 | 0.5533 | 0.4871 |
| *4* | 0.4579 | 0.7326 | 0.8680 | 1.0000 | 0.9472 | 0.8660 | 0.7813 | 0.7214 |
| *5* | 0.3828 | 0.6022 | 0.7586 | 0.9472 | 1.0000 | 0.9593 | 0.8854 | 0.8248 |
| *6* | 0.3041 | 0.5117 | 0.6555 | 0.8660 | 0.9593 | 1.0000 | 0.9687 | 0.9243 |
| *7* | 0.2389 | 0.4194 | 0.5533 | 0.7813 | 0.8854 | 0.9687 | 1.0000 | 0.9807 |
| *8* | 0.1973 | 0.3593 | 0.4871 | 0.7214 | 0.8248 | 0.9243 | 0.9807 | 1.0000 |

| *Covariance Parameter Estimates* | | |
| --- | --- | --- |
| *Cov Parm* | *Subject* | *Estimate* |
| *UN(1,1)* | REP*NAME*TREAT*EXP | 0.09942 |
| *UN(2,1)* | REP*NAME*TREAT*EXP | 0.08012 |
| *UN(2,2)* | REP*NAME*TREAT*EXP | 0.1180 |
| *UN(3,1)* | REP*NAME*TREAT*EXP | 0.06093 |
| *UN(3,2)* | REP*NAME*TREAT*EXP | 0.1066 |
| *UN(3,3)* | REP*NAME*TREAT*EXP | 0.1241 |
| *UN(4,1)* | REP*NAME*TREAT*EXP | 0.04748 |
| *UN(4,2)* | REP*NAME*TREAT*EXP | 0.08274 |
| *UN(4,3)* | REP*NAME*TREAT*EXP | 0.1006 |
| *UN(4,4)* | REP*NAME*TREAT*EXP | 0.1081 |
| *UN(5,1)* | REP*NAME*TREAT*EXP | 0.03750 |
| *UN(5,2)* | REP*NAME*TREAT*EXP | 0.06428 |
| *UN(5,3)* | REP*NAME*TREAT*EXP | 0.08305 |
| *UN(5,4)* | REP*NAME*TREAT*EXP | 0.09679 |
| *UN(5,5)* | REP*NAME*TREAT*EXP | 0.09656 |
| *UN(6,1)* | REP*NAME*TREAT*EXP | 0.02993 |
| *UN(6,2)* | REP*NAME*TREAT*EXP | 0.05487 |
| *UN(6,3)* | REP*NAME*TREAT*EXP | 0.07210 |
| *UN(6,4)* | REP*NAME*TREAT*EXP | 0.08890 |
| *UN(6,5)* | REP*NAME*TREAT*EXP | 0.09306 |
| *UN(6,6)* | REP*NAME*TREAT*EXP | 0.09747 |
| *UN(7,1)* | REP*NAME*TREAT*EXP | 0.02379 |
| *UN(7,2)* | REP*NAME*TREAT*EXP | 0.04549 |
| *UN(7,3)* | REP*NAME*TREAT*EXP | 0.06156 |
| *UN(7,4)* | REP*NAME*TREAT*EXP | 0.08112 |
| *UN(7,5)* | REP*NAME*TREAT*EXP | 0.08688 |
| *UN(7,6)* | REP*NAME*TREAT*EXP | 0.09550 |
| *UN(7,7)* | REP*NAME*TREAT*EXP | 0.09973 |
| *UN(8,1)* | REP*NAME*TREAT*EXP | 0.02013 |
| *UN(8,2)* | REP*NAME*TREAT*EXP | 0.03993 |
| *UN(8,3)* | REP*NAME*TREAT*EXP | 0.05552 |
| *UN(8,4)* | REP*NAME*TREAT*EXP | 0.07674 |
| *UN(8,5)* | REP*NAME*TREAT*EXP | 0.08293 |
| *UN(8,6)* | REP*NAME*TREAT*EXP | 0.09336 |
| *UN(8,7)* | REP*NAME*TREAT*EXP | 0.1002 |
| *UN(8,8)* | REP*NAME*TREAT*EXP | 0.1047 |

| *Fit Statistics* | |
| --- | --- |
| *-2 Res Log Likelihood* | -1019.5 |
| *AIC (smaller is better)* | -947.5 |
| *AICC (smaller is better)* | -944.6 |
| *BIC (smaller is better)* | -840.6 |

| *Null Model Likelihood Ratio Test* | | |
| --- | --- | --- |
| *DF* | *Chi-Square* | *Pr > ChiSq* |
| 35 | 1926.93 | <.0001 |

| *Type 3 Tests of Fixed Effects* | | | | |
| --- | --- | --- | --- | --- |
| *Effect* | *Num DF* | *Den DF* | *F Value* | *Pr > F* |
| *EXP* | 2 | 118 | 17.38 | <.0001 |
| *NAME* | 11 | 118 | 10.91 | <.0001 |
| *TREAT* | 1 | 118 | 585.40 | <.0001 |
| *NAME*TREAT* | 11 | 118 | 4.44 | <.0001 |
| *TIME* | 7 | 118 | 688.76 | <.0001 |
| *NAME*TIME* | 77 | 118 | 3.62 | <.0001 |
| *TREAT*TIME* | 7 | 118 | 87.55 | <.0001 |
| *NAME*TREAT*TIME* | 77 | 118 | 2.60 | <.0001 |

| *Least Squares Means* | | | | | | | | |
| --- | --- | --- | --- | --- | --- | --- | --- | --- |
| *Effect* | *NAME* | *TREAT* | *TIME* | *Estimate* | *Standard Error* | *DF* | *t Value* | *Pr > \|t\|* |
| *NAME*TREAT*TIME* | ALGANS | cold | 1 | 0.07492 | 0.1287 | 118 | 0.58 | 0.5617 |
| *NAME*TREAT*TIME* | ALGANS | cold | 2 | 0.2420 | 0.1402 | 118 | 1.73 | 0.0870 |
| *NAME*TREAT*TIME* | ALGANS | cold | 3 | 0.3639 | 0.1438 | 118 | 2.53 | 0.0127 |
| *NAME*TREAT*TIME* | ALGANS | cold | 4 | 0.5977 | 0.1342 | 118 | 4.45 | <.0001 |
| *NAME*TREAT*TIME* | ALGANS | cold | 5 | 0.6930 | 0.1269 | 118 | 5.46 | <.0001 |
| *NAME*TREAT*TIME* | ALGANS | cold | 6 | 0.8968 | 0.1275 | 118 | 7.04 | <.0001 |
| *NAME*TREAT*TIME* | ALGANS | cold | 7 | 0.9994 | 0.1289 | 118 | 7.75 | <.0001 |
| *NAME*TREAT*TIME* | ALGANS | cold | 8 | 1.1246 | 0.1321 | 118 | 8.51 | <.0001 |
| *NAME*TREAT*TIME* | ALGANS | control | 1 | 0.4732 | 0.1287 | 118 | 3.68 | 0.0004 |
| *NAME*TREAT*TIME* | ALGANS | control | 2 | 1.1356 | 0.1402 | 118 | 8.10 | <.0001 |
| *NAME*TREAT*TIME* | ALGANS | control | 3 | 1.8373 | 0.1438 | 118 | 12.77 | <.0001 |
| *NAME*TREAT*TIME* | ALGANS | control | 4 | 2.2120 | 0.1342 | 118 | 16.48 | <.0001 |
| *NAME*TREAT*TIME* | ALGANS | control | 5 | 2.4398 | 0.1269 | 118 | 19.23 | <.0001 |
| *NAME*TREAT*TIME* | ALGANS | control | 6 | 2.6942 | 0.1275 | 118 | 21.14 | <.0001 |
| *NAME*TREAT*TIME* | ALGANS | control | 7 | 2.8990 | 0.1289 | 118 | 22.49 | <.0001 |
| *NAME*TREAT*TIME* | ALGANS | control | 8 | 3.0917 | 0.1321 | 118 | 23.41 | <.0001 |
| *NAME*TREAT*TIME* | CLARITI | cold | 1 | 0.4533 | 0.1287 | 118 | 3.52 | 0.0006 |
| *NAME*TREAT*TIME* | CLARITI | cold | 2 | 0.7277 | 0.1402 | 118 | 5.19 | <.0001 |
| *NAME*TREAT*TIME* | CLARITI | cold | 3 | 1.0180 | 0.1438 | 118 | 7.08 | <.0001 |
| *NAME*TREAT*TIME* | CLARITI | cold | 4 | 1.3275 | 0.1342 | 118 | 9.89 | <.0001 |
| *NAME*TREAT*TIME* | CLARITI | cold | 5 | 1.4816 | 0.1269 | 118 | 11.68 | <.0001 |
| *NAME*TREAT*TIME* | CLARITI | cold | 6 | 1.6341 | 0.1275 | 118 | 12.82 | <.0001 |
| *NAME*TREAT*TIME* | CLARITI | cold | 7 | 1.7796 | 0.1289 | 118 | 13.80 | <.0001 |
| *NAME*TREAT*TIME* | CLARITI | cold | 8 | 1.9712 | 0.1321 | 118 | 14.92 | <.0001 |
| *NAME*TREAT*TIME* | CLARITI | control | 1 | 0.4907 | 0.1287 | 118 | 3.81 | 0.0002 |
| *NAME*TREAT*TIME* | CLARITI | control | 2 | 1.6199 | 0.1402 | 118 | 11.55 | <.0001 |
| *NAME*TREAT*TIME* | CLARITI | control | 3 | 2.2191 | 0.1438 | 118 | 15.43 | <.0001 |
| *NAME*TREAT*TIME* | CLARITI | control | 4 | 2.6744 | 0.1342 | 118 | 19.92 | <.0001 |
| *NAME*TREAT*TIME* | CLARITI | control | 5 | 2.9219 | 0.1269 | 118 | 23.03 | <.0001 |
| *NAME*TREAT*TIME* | CLARITI | control | 6 | 3.0861 | 0.1275 | 118 | 24.21 | <.0001 |
| *NAME*TREAT*TIME* | CLARITI | control | 7 | 3.2978 | 0.1289 | 118 | 25.58 | <.0001 |
| *NAME*TREAT*TIME* | CLARITI | control | 8 | 3.4500 | 0.1321 | 118 | 26.12 | <.0001 |
| *NAME*TREAT*TIME* | CODIFAR | cold | 1 | 0.7321 | 0.1287 | 118 | 5.69 | <.0001 |
| *NAME*TREAT*TIME* | CODIFAR | cold | 2 | 1.0631 | 0.1402 | 118 | 7.58 | <.0001 |
| *NAME*TREAT*TIME* | CODIFAR | cold | 3 | 1.3733 | 0.1438 | 118 | 9.55 | <.0001 |
| *NAME*TREAT*TIME* | CODIFAR | cold | 4 | 1.7363 | 0.1342 | 118 | 12.93 | <.0001 |
| *NAME*TREAT*TIME* | CODIFAR | cold | 5 | 1.9456 | 0.1269 | 118 | 15.34 | <.0001 |
| *NAME*TREAT*TIME* | CODIFAR | cold | 6 | 2.0760 | 0.1275 | 118 | 16.29 | <.0001 |
| *NAME*TREAT*TIME* | CODIFAR | cold | 7 | 2.1866 | 0.1289 | 118 | 16.96 | <.0001 |
| *NAME*TREAT*TIME* | CODIFAR | cold | 8 | 2.3321 | 0.1321 | 118 | 17.66 | <.0001 |
| *NAME*TREAT*TIME* | CODIFAR | control | 1 | 0.4120 | 0.1287 | 118 | 3.20 | 0.0018 |
| *NAME*TREAT*TIME* | CODIFAR | control | 2 | 1.3767 | 0.1402 | 118 | 9.82 | <.0001 |
| *NAME*TREAT*TIME* | CODIFAR | control | 3 | 1.8418 | 0.1438 | 118 | 12.81 | <.0001 |
| *NAME*TREAT*TIME* | CODIFAR | control | 4 | 2.2680 | 0.1342 | 118 | 16.90 | <.0001 |
| *NAME*TREAT*TIME* | CODIFAR | control | 5 | 2.6503 | 0.1269 | 118 | 20.89 | <.0001 |
| *NAME*TREAT*TIME* | CODIFAR | control | 6 | 2.9869 | 0.1275 | 118 | 23.43 | <.0001 |
| *NAME*TREAT*TIME* | CODIFAR | control | 7 | 3.2492 | 0.1289 | 118 | 25.20 | <.0001 |
| *NAME*TREAT*TIME* | CODIFAR | control | 8 | 3.5455 | 0.1321 | 118 | 26.84 | <.0001 |
| *NAME*TREAT*TIME* | CODISCO | cold | 1 | 0.2530 | 0.1287 | 118 | 1.97 | 0.0517 |
| *NAME*TREAT*TIME* | CODISCO | cold | 2 | 0.4671 | 0.1402 | 118 | 3.33 | 0.0012 |
| *NAME*TREAT*TIME* | CODISCO | cold | 3 | 0.7099 | 0.1438 | 118 | 4.94 | <.0001 |
| *NAME*TREAT*TIME* | CODISCO | cold | 4 | 0.8611 | 0.1342 | 118 | 6.41 | <.0001 |
| *NAME*TREAT*TIME* | CODISCO | cold | 5 | 1.0899 | 0.1269 | 118 | 8.59 | <.0001 |
| *NAME*TREAT*TIME* | CODISCO | cold | 6 | 1.2326 | 0.1275 | 118 | 9.67 | <.0001 |
| *NAME*TREAT*TIME* | CODISCO | cold | 7 | 1.3356 | 0.1289 | 118 | 10.36 | <.0001 |
| *NAME*TREAT*TIME* | CODISCO | cold | 8 | 1.5214 | 0.1321 | 118 | 11.52 | <.0001 |
| *NAME*TREAT*TIME* | CODISCO | control | 1 | 0.8125 | 0.1287 | 118 | 6.31 | <.0001 |
| *NAME*TREAT*TIME* | CODISCO | control | 2 | 1.6792 | 0.1402 | 118 | 11.97 | <.0001 |
| *NAME*TREAT*TIME* | CODISCO | control | 3 | 2.1812 | 0.1438 | 118 | 15.17 | <.0001 |
| *NAME*TREAT*TIME* | CODISCO | control | 4 | 2.5228 | 0.1342 | 118 | 18.79 | <.0001 |
| *NAME*TREAT*TIME* | CODISCO | control | 5 | 2.7216 | 0.1269 | 118 | 21.45 | <.0001 |
| *NAME*TREAT*TIME* | CODISCO | control | 6 | 2.8647 | 0.1275 | 118 | 22.48 | <.0001 |
| *NAME*TREAT*TIME* | CODISCO | control | 7 | 3.0367 | 0.1289 | 118 | 23.55 | <.0001 |
| *NAME*TREAT*TIME* | CODISCO | control | 8 | 3.2578 | 0.1321 | 118 | 24.66 | <.0001 |
| *NAME*TREAT*TIME* | CRAZI | cold | 1 | 0.5437 | 0.1287 | 118 | 4.22 | <.0001 |
| *NAME*TREAT*TIME* | CRAZI | cold | 2 | 0.8369 | 0.1402 | 118 | 5.97 | <.0001 |
| *NAME*TREAT*TIME* | CRAZI | cold | 3 | 1.2737 | 0.1438 | 118 | 8.86 | <.0001 |
| *NAME*TREAT*TIME* | CRAZI | cold | 4 | 1.5142 | 0.1342 | 118 | 11.28 | <.0001 |
| *NAME*TREAT*TIME* | CRAZI | cold | 5 | 1.7039 | 0.1269 | 118 | 13.43 | <.0001 |
| *NAME*TREAT*TIME* | CRAZI | cold | 6 | 1.8177 | 0.1275 | 118 | 14.26 | <.0001 |
| *NAME*TREAT*TIME* | CRAZI | cold | 7 | 1.9415 | 0.1289 | 118 | 15.06 | <.0001 |
| *NAME*TREAT*TIME* | CRAZI | cold | 8 | 2.0924 | 0.1321 | 118 | 15.84 | <.0001 |
| *NAME*TREAT*TIME* | CRAZI | control | 1 | 0.4147 | 0.1287 | 118 | 3.22 | 0.0016 |
| *NAME*TREAT*TIME* | CRAZI | control | 2 | 1.3243 | 0.1402 | 118 | 9.44 | <.0001 |
| *NAME*TREAT*TIME* | CRAZI | control | 3 | 1.9110 | 0.1438 | 118 | 13.29 | <.0001 |
| *NAME*TREAT*TIME* | CRAZI | control | 4 | 2.4125 | 0.1342 | 118 | 17.97 | <.0001 |
| *NAME*TREAT*TIME* | CRAZI | control | 5 | 2.7739 | 0.1269 | 118 | 21.87 | <.0001 |
| *NAME*TREAT*TIME* | CRAZI | control | 6 | 3.0105 | 0.1275 | 118 | 23.62 | <.0001 |
| *NAME*TREAT*TIME* | CRAZI | control | 7 | 3.2177 | 0.1289 | 118 | 24.96 | <.0001 |
| *NAME*TREAT*TIME* | CRAZI | control | 8 | 3.3890 | 0.1321 | 118 | 25.66 | <.0001 |
| *NAME*TREAT*TIME* | FERGUS | cold | 1 | 0.2080 | 0.1287 | 118 | 1.62 | 0.1088 |
| *NAME*TREAT*TIME* | FERGUS | cold | 2 | 0.4484 | 0.1402 | 118 | 3.20 | 0.0018 |
| *NAME*TREAT*TIME* | FERGUS | cold | 3 | 0.6312 | 0.1438 | 118 | 4.39 | <.0001 |
| *NAME*TREAT*TIME* | FERGUS | cold | 4 | 0.8492 | 0.1342 | 118 | 6.33 | <.0001 |
| *NAME*TREAT*TIME* | FERGUS | cold | 5 | 1.0805 | 0.1269 | 118 | 8.52 | <.0001 |
| *NAME*TREAT*TIME* | FERGUS | cold | 6 | 1.2228 | 0.1275 | 118 | 9.59 | <.0001 |
| *NAME*TREAT*TIME* | FERGUS | cold | 7 | 1.3716 | 0.1289 | 118 | 10.64 | <.0001 |
| *NAME*TREAT*TIME* | FERGUS | cold | 8 | 1.5438 | 0.1321 | 118 | 11.69 | <.0001 |
| *NAME*TREAT*TIME* | FERGUS | control | 1 | 0.6368 | 0.1287 | 118 | 4.95 | <.0001 |
| *NAME*TREAT*TIME* | FERGUS | control | 2 | 1.6536 | 0.1402 | 118 | 11.79 | <.0001 |
| *NAME*TREAT*TIME* | FERGUS | control | 3 | 2.1229 | 0.1438 | 118 | 14.76 | <.0001 |
| *NAME*TREAT*TIME* | FERGUS | control | 4 | 2.7415 | 0.1342 | 118 | 20.42 | <.0001 |
| *NAME*TREAT*TIME* | FERGUS | control | 5 | 3.1257 | 0.1269 | 118 | 24.64 | <.0001 |
| *NAME*TREAT*TIME* | FERGUS | control | 6 | 3.2844 | 0.1275 | 118 | 25.77 | <.0001 |
| *NAME*TREAT*TIME* | FERGUS | control | 7 | 3.4146 | 0.1289 | 118 | 26.49 | <.0001 |
| *NAME*TREAT*TIME* | FERGUS | control | 8 | 3.6240 | 0.1321 | 118 | 27.44 | <.0001 |
| *NAME*TREAT*TIME* | HUSKI | cold | 1 | 0.2737 | 0.1287 | 118 | 2.13 | 0.0355 |
| *NAME*TREAT*TIME* | HUSKI | cold | 2 | 0.5403 | 0.1402 | 118 | 3.85 | 0.0002 |
| *NAME*TREAT*TIME* | HUSKI | cold | 3 | 1.1530 | 0.1438 | 118 | 8.02 | <.0001 |
| *NAME*TREAT*TIME* | HUSKI | cold | 4 | 1.3682 | 0.1342 | 118 | 10.19 | <.0001 |
| *NAME*TREAT*TIME* | HUSKI | cold | 5 | 1.6154 | 0.1269 | 118 | 12.73 | <.0001 |
| *NAME*TREAT*TIME* | HUSKI | cold | 6 | 1.7204 | 0.1275 | 118 | 13.50 | <.0001 |
| *NAME*TREAT*TIME* | HUSKI | cold | 7 | 1.8223 | 0.1289 | 118 | 14.13 | <.0001 |
| *NAME*TREAT*TIME* | HUSKI | cold | 8 | 1.9609 | 0.1321 | 118 | 14.85 | <.0001 |
| *NAME*TREAT*TIME* | HUSKI | control | 1 | 0.4937 | 0.1287 | 118 | 3.84 | 0.0002 |
| *NAME*TREAT*TIME* | HUSKI | control | 2 | 1.2574 | 0.1402 | 118 | 8.97 | <.0001 |
| *NAME*TREAT*TIME* | HUSKI | control | 3 | 1.7401 | 0.1438 | 118 | 12.10 | <.0001 |
| *NAME*TREAT*TIME* | HUSKI | control | 4 | 2.3784 | 0.1342 | 118 | 17.72 | <.0001 |
| *NAME*TREAT*TIME* | HUSKI | control | 5 | 2.7803 | 0.1269 | 118 | 21.92 | <.0001 |
| *NAME*TREAT*TIME* | HUSKI | control | 6 | 2.9168 | 0.1275 | 118 | 22.88 | <.0001 |
| *NAME*TREAT*TIME* | HUSKI | control | 7 | 3.0467 | 0.1289 | 118 | 23.63 | <.0001 |
| *NAME*TREAT*TIME* | HUSKI | control | 8 | 3.2850 | 0.1321 | 118 | 24.87 | <.0001 |
| *NAME*TREAT*TIME* | JUSTINA | cold | 1 | 0.1679 | 0.1287 | 118 | 1.30 | 0.1945 |
| *NAME*TREAT*TIME* | JUSTINA | cold | 2 | 0.1823 | 0.1402 | 118 | 1.30 | 0.1961 |
| *NAME*TREAT*TIME* | JUSTINA | cold | 3 | 0.4410 | 0.1438 | 118 | 3.07 | 0.0027 |
| *NAME*TREAT*TIME* | JUSTINA | cold | 4 | 0.5133 | 0.1342 | 118 | 3.82 | 0.0002 |
| *NAME*TREAT*TIME* | JUSTINA | cold | 5 | 0.6430 | 0.1269 | 118 | 5.07 | <.0001 |
| *NAME*TREAT*TIME* | JUSTINA | cold | 6 | 0.8271 | 0.1275 | 118 | 6.49 | <.0001 |
| *NAME*TREAT*TIME* | JUSTINA | cold | 7 | 0.9489 | 0.1289 | 118 | 7.36 | <.0001 |
| *NAME*TREAT*TIME* | JUSTINA | cold | 8 | 1.0421 | 0.1321 | 118 | 7.89 | <.0001 |
| *NAME*TREAT*TIME* | JUSTINA | control | 1 | 0.3866 | 0.1287 | 118 | 3.00 | 0.0033 |
| *NAME*TREAT*TIME* | JUSTINA | control | 2 | 1.1742 | 0.1402 | 118 | 8.37 | <.0001 |
| *NAME*TREAT*TIME* | JUSTINA | control | 3 | 1.6330 | 0.1438 | 118 | 11.35 | <.0001 |
| *NAME*TREAT*TIME* | JUSTINA | control | 4 | 2.0752 | 0.1342 | 118 | 15.46 | <.0001 |
| *NAME*TREAT*TIME* | JUSTINA | control | 5 | 2.3495 | 0.1269 | 118 | 18.52 | <.0001 |
| *NAME*TREAT*TIME* | JUSTINA | control | 6 | 2.5696 | 0.1275 | 118 | 20.16 | <.0001 |
| *NAME*TREAT*TIME* | JUSTINA | control | 7 | 2.8570 | 0.1289 | 118 | 22.16 | <.0001 |
| *NAME*TREAT*TIME* | JUSTINA | control | 8 | 3.1683 | 0.1321 | 118 | 23.99 | <.0001 |
| *NAME*TREAT*TIME* | LAKTI | cold | 1 | 0.2953 | 0.1287 | 118 | 2.29 | 0.0235 |
| *NAME*TREAT*TIME* | LAKTI | cold | 2 | 0.6252 | 0.1402 | 118 | 4.46 | <.0001 |
| *NAME*TREAT*TIME* | LAKTI | cold | 3 | 0.9145 | 0.1438 | 118 | 6.36 | <.0001 |
| *NAME*TREAT*TIME* | LAKTI | cold | 4 | 1.2404 | 0.1342 | 118 | 9.24 | <.0001 |
| *NAME*TREAT*TIME* | LAKTI | cold | 5 | 1.4362 | 0.1269 | 118 | 11.32 | <.0001 |
| *NAME*TREAT*TIME* | LAKTI | cold | 6 | 1.6558 | 0.1275 | 118 | 12.99 | <.0001 |
| *NAME*TREAT*TIME* | LAKTI | cold | 7 | 1.8340 | 0.1289 | 118 | 14.23 | <.0001 |
| *NAME*TREAT*TIME* | LAKTI | cold | 8 | 2.0364 | 0.1321 | 118 | 15.42 | <.0001 |
| *NAME*TREAT*TIME* | LAKTI | control | 1 | 0.2435 | 0.1287 | 118 | 1.89 | 0.0610 |
| *NAME*TREAT*TIME* | LAKTI | control | 2 | 1.2316 | 0.1402 | 118 | 8.78 | <.0001 |
| *NAME*TREAT*TIME* | LAKTI | control | 3 | 1.5866 | 0.1438 | 118 | 11.03 | <.0001 |
| *NAME*TREAT*TIME* | LAKTI | control | 4 | 2.3057 | 0.1342 | 118 | 17.18 | <.0001 |
| *NAME*TREAT*TIME* | LAKTI | control | 5 | 2.7502 | 0.1269 | 118 | 21.68 | <.0001 |
| *NAME*TREAT*TIME* | LAKTI | control | 6 | 2.9586 | 0.1275 | 118 | 23.21 | <.0001 |
| *NAME*TREAT*TIME* | LAKTI | control | 7 | 3.1231 | 0.1289 | 118 | 24.22 | <.0001 |
| *NAME*TREAT*TIME* | LAKTI | control | 8 | 3.3210 | 0.1321 | 118 | 25.14 | <.0001 |
| *NAME*TREAT*TIME* | P329D60 | cold | 1 | 0.4757 | 0.1287 | 118 | 3.70 | 0.0003 |
| *NAME*TREAT*TIME* | P329D60 | cold | 2 | 0.9349 | 0.1402 | 118 | 6.67 | <.0001 |
| *NAME*TREAT*TIME* | P329D60 | cold | 3 | 1.3118 | 0.1438 | 118 | 9.12 | <.0001 |
| *NAME*TREAT*TIME* | P329D60 | cold | 4 | 1.4864 | 0.1342 | 118 | 11.07 | <.0001 |
| *NAME*TREAT*TIME* | P329D60 | cold | 5 | 1.6572 | 0.1269 | 118 | 13.06 | <.0001 |
| *NAME*TREAT*TIME* | P329D60 | cold | 6 | 1.8819 | 0.1275 | 118 | 14.77 | <.0001 |
| *NAME*TREAT*TIME* | P329D60 | cold | 7 | 2.0043 | 0.1289 | 118 | 15.55 | <.0001 |
| *NAME*TREAT*TIME* | P329D60 | cold | 8 | 2.1018 | 0.1321 | 118 | 15.91 | <.0001 |
| *NAME*TREAT*TIME* | P329D60 | control | 1 | 0.6933 | 0.1287 | 118 | 5.39 | <.0001 |
| *NAME*TREAT*TIME* | P329D60 | control | 2 | 1.6568 | 0.1402 | 118 | 11.81 | <.0001 |
| *NAME*TREAT*TIME* | P329D60 | control | 3 | 2.1233 | 0.1438 | 118 | 14.76 | <.0001 |
| *NAME*TREAT*TIME* | P329D60 | control | 4 | 2.6182 | 0.1342 | 118 | 19.50 | <.0001 |
| *NAME*TREAT*TIME* | P329D60 | control | 5 | 2.9431 | 0.1269 | 118 | 23.20 | <.0001 |
| *NAME*TREAT*TIME* | P329D60 | control | 6 | 3.1907 | 0.1275 | 118 | 25.03 | <.0001 |
| *NAME*TREAT*TIME* | P329D60 | control | 7 | 3.4322 | 0.1289 | 118 | 26.62 | <.0001 |
| *NAME*TREAT*TIME* | P329D60 | control | 8 | 3.6289 | 0.1321 | 118 | 27.47 | <.0001 |
| *NAME*TREAT*TIME* | PICKER | cold | 1 | 0.7025 | 0.1287 | 118 | 5.46 | <.0001 |
| *NAME*TREAT*TIME* | PICKER | cold | 2 | 1.1937 | 0.1402 | 118 | 8.51 | <.0001 |
| *NAME*TREAT*TIME* | PICKER | cold | 3 | 1.6087 | 0.1438 | 118 | 11.18 | <.0001 |
| *NAME*TREAT*TIME* | PICKER | cold | 4 | 1.8203 | 0.1342 | 118 | 13.56 | <.0001 |
| *NAME*TREAT*TIME* | PICKER | cold | 5 | 1.9623 | 0.1269 | 118 | 15.47 | <.0001 |
| *NAME*TREAT*TIME* | PICKER | cold | 6 | 2.0768 | 0.1275 | 118 | 16.29 | <.0001 |
| *NAME*TREAT*TIME* | PICKER | cold | 7 | 2.2144 | 0.1289 | 118 | 17.18 | <.0001 |
| *NAME*TREAT*TIME* | PICKER | cold | 8 | 2.3602 | 0.1321 | 118 | 17.87 | <.0001 |
| *NAME*TREAT*TIME* | PICKER | control | 1 | 0.6984 | 0.1287 | 118 | 5.43 | <.0001 |
| *NAME*TREAT*TIME* | PICKER | control | 2 | 1.8342 | 0.1402 | 118 | 13.08 | <.0001 |
| *NAME*TREAT*TIME* | PICKER | control | 3 | 2.4435 | 0.1438 | 118 | 16.99 | <.0001 |
| *NAME*TREAT*TIME* | PICKER | control | 4 | 2.8917 | 0.1342 | 118 | 21.54 | <.0001 |
| *NAME*TREAT*TIME* | PICKER | control | 5 | 3.1335 | 0.1269 | 118 | 24.70 | <.0001 |
| *NAME*TREAT*TIME* | PICKER | control | 6 | 3.2885 | 0.1275 | 118 | 25.80 | <.0001 |
| *NAME*TREAT*TIME* | PICKER | control | 7 | 3.4417 | 0.1289 | 118 | 26.70 | <.0001 |
| *NAME*TREAT*TIME* | PICKER | control | 8 | 3.5807 | 0.1321 | 118 | 27.11 | <.0001 |
| *NAME*TREAT*TIME* | PR29B29 | cold | 1 | 0.2748 | 0.1287 | 118 | 2.14 | 0.0348 |
| *NAME*TREAT*TIME* | PR29B29 | cold | 2 | 0.5620 | 0.1402 | 118 | 4.01 | 0.0001 |
| *NAME*TREAT*TIME* | PR29B29 | cold | 3 | 0.7747 | 0.1438 | 118 | 5.39 | <.0001 |
| *NAME*TREAT*TIME* | PR29B29 | cold | 4 | 1.0578 | 0.1342 | 118 | 7.88 | <.0001 |
| *NAME*TREAT*TIME* | PR29B29 | cold | 5 | 1.3945 | 0.1269 | 118 | 10.99 | <.0001 |
| *NAME*TREAT*TIME* | PR29B29 | cold | 6 | 1.6997 | 0.1275 | 118 | 13.34 | <.0001 |
| *NAME*TREAT*TIME* | PR29B29 | cold | 7 | 1.8805 | 0.1289 | 118 | 14.59 | <.0001 |
| *NAME*TREAT*TIME* | PR29B29 | cold | 8 | 2.0460 | 0.1321 | 118 | 15.49 | <.0001 |
| *NAME*TREAT*TIME* | PR29B29 | control | 1 | 0.2404 | 0.1287 | 118 | 1.87 | 0.0642 |
| *NAME*TREAT*TIME* | PR29B29 | control | 2 | 0.6904 | 0.1402 | 118 | 4.92 | <.0001 |
| *NAME*TREAT*TIME* | PR29B29 | control | 3 | 1.4166 | 0.1438 | 118 | 9.85 | <.0001 |
| *NAME*TREAT*TIME* | PR29B29 | control | 4 | 1.9642 | 0.1342 | 118 | 14.63 | <.0001 |
| *NAME*TREAT*TIME* | PR29B29 | control | 5 | 2.3261 | 0.1269 | 118 | 18.34 | <.0001 |
| *NAME*TREAT*TIME* | PR29B29 | control | 6 | 2.6830 | 0.1275 | 118 | 21.05 | <.0001 |
| *NAME*TREAT*TIME* | PR29B29 | control | 7 | 3.0069 | 0.1289 | 118 | 23.32 | <.0001 |
| *NAME*TREAT*TIME* | PR29B29 | control | 8 | 3.2662 | 0.1321 | 118 | 24.73 | <.0001 |

| *Tests of Effect Slices* | | | | | | | |
| --- | --- | --- | --- | --- | --- | --- | --- |
| *Effect* | *NAME* | *TREAT* | *TIME* | *Num DF* | *Den DF* | *F Value* | *Pr > F* |
| *NAME*TREAT*TIME* | ALGANS |  |  | 15 | 118 | 27.47 | <.0001 |
| *NAME*TREAT*TIME* | CLARITI |  |  | 15 | 118 | 35.40 | <.0001 |
| *NAME*TREAT*TIME* | CODIFAR |  |  | 15 | 118 | 38.48 | <.0001 |
| *NAME*TREAT*TIME* | CODISCO |  |  | 15 | 118 | 30.51 | <.0001 |
| *NAME*TREAT*TIME* | CRAZI |  |  | 15 | 118 | 33.14 | <.0001 |
| *NAME*TREAT*TIME* | FERGUS |  |  | 15 | 118 | 42.18 | <.0001 |
| *NAME*TREAT*TIME* | HUSKI |  |  | 15 | 118 | 39.51 | <.0001 |
| *NAME*TREAT*TIME* | JUSTINA |  |  | 15 | 118 | 33.86 | <.0001 |
| *NAME*TREAT*TIME* | LAKTI |  |  | 15 | 118 | 42.39 | <.0001 |
| *NAME*TREAT*TIME* | P329D60 |  |  | 15 | 118 | 34.64 | <.0001 |
| *NAME*TREAT*TIME* | PICKER |  |  | 15 | 118 | 33.52 | <.0001 |
| *NAME*TREAT*TIME* | PR29B29 |  |  | 15 | 118 | 36.21 | <.0001 |
| *NAME*TREAT*TIME* |  | cold |  | 95 | 118 | 15.25 | <.0001 |
| *NAME*TREAT*TIME* |  | control |  | 95 | 118 | 48.53 | <.0001 |
| *NAME*TREAT*TIME* |  |  | 1 | 23 | 118 | 2.45 | 0.0009 |
| *NAME*TREAT*TIME* |  |  | 2 | 23 | 118 | 11.99 | <.0001 |
| *NAME*TREAT*TIME* |  |  | 3 | 23 | 118 | 17.21 | <.0001 |
| *NAME*TREAT*TIME* |  |  | 4 | 23 | 118 | 28.55 | <.0001 |
| *NAME*TREAT*TIME* |  |  | 5 | 23 | 118 | 37.42 | <.0001 |
| *NAME*TREAT*TIME* |  |  | 6 | 23 | 118 | 38.29 | <.0001 |
| *NAME*TREAT*TIME* |  |  | 7 | 23 | 118 | 40.65 | <.0001 |
| *NAME*TREAT*TIME* |  |  | 8 | 23 | 118 | 41.57 | <.0001 |
| *NAME*TREAT*TIME* | ALGANS |  | 1 | 1 | 118 | 4.79 | 0.0307 |
| *NAME*TREAT*TIME* | ALGANS |  | 2 | 1 | 118 | 20.30 | <.0001 |
| *NAME*TREAT*TIME* | ALGANS |  | 3 | 1 | 118 | 52.47 | <.0001 |
| *NAME*TREAT*TIME* | ALGANS |  | 4 | 1 | 118 | 72.30 | <.0001 |
| *NAME*TREAT*TIME* | ALGANS |  | 5 | 1 | 118 | 94.80 | <.0001 |
| *NAME*TREAT*TIME* | ALGANS |  | 6 | 1 | 118 | 99.44 | <.0001 |
| *NAME*TREAT*TIME* | ALGANS |  | 7 | 1 | 118 | 108.54 | <.0001 |
| *NAME*TREAT*TIME* | ALGANS |  | 8 | 1 | 118 | 110.89 | <.0001 |
| *NAME*TREAT*TIME* | CLARITI |  | 1 | 1 | 118 | 0.04 | 0.8376 |
| *NAME*TREAT*TIME* | CLARITI |  | 2 | 1 | 118 | 20.24 | <.0001 |
| *NAME*TREAT*TIME* | CLARITI |  | 3 | 1 | 118 | 34.87 | <.0001 |
| *NAME*TREAT*TIME* | CLARITI |  | 4 | 1 | 118 | 50.33 | <.0001 |
| *NAME*TREAT*TIME* | CLARITI |  | 5 | 1 | 118 | 64.45 | <.0001 |
| *NAME*TREAT*TIME* | CLARITI |  | 6 | 1 | 118 | 64.90 | <.0001 |
| *NAME*TREAT*TIME* | CLARITI |  | 7 | 1 | 118 | 69.34 | <.0001 |
| *NAME*TREAT*TIME* | CLARITI |  | 8 | 1 | 118 | 62.68 | <.0001 |
| *NAME*TREAT*TIME* | CODIFAR |  | 1 | 1 | 118 | 3.09 | 0.0813 |
| *NAME*TREAT*TIME* | CODIFAR |  | 2 | 1 | 118 | 2.50 | 0.1166 |
| *NAME*TREAT*TIME* | CODIFAR |  | 3 | 1 | 118 | 5.31 | 0.0230 |
| *NAME*TREAT*TIME* | CODIFAR |  | 4 | 1 | 118 | 7.84 | 0.0060 |
| *NAME*TREAT*TIME* | CODIFAR |  | 5 | 1 | 118 | 15.43 | 0.0001 |
| *NAME*TREAT*TIME* | CODIFAR |  | 6 | 1 | 118 | 25.54 | <.0001 |
| *NAME*TREAT*TIME* | CODIFAR |  | 7 | 1 | 118 | 33.97 | <.0001 |
| *NAME*TREAT*TIME* | CODIFAR |  | 8 | 1 | 118 | 42.20 | <.0001 |
| *NAME*TREAT*TIME* | CODISCO |  | 1 | 1 | 118 | 9.44 | 0.0026 |
| *NAME*TREAT*TIME* | CODISCO |  | 2 | 1 | 118 | 37.36 | <.0001 |
| *NAME*TREAT*TIME* | CODISCO |  | 3 | 1 | 118 | 52.32 | <.0001 |
| *NAME*TREAT*TIME* | CODISCO |  | 4 | 1 | 118 | 76.61 | <.0001 |
| *NAME*TREAT*TIME* | CODISCO |  | 5 | 1 | 118 | 82.71 | <.0001 |
| *NAME*TREAT*TIME* | CODISCO |  | 6 | 1 | 118 | 81.98 | <.0001 |
| *NAME*TREAT*TIME* | CODISCO |  | 7 | 1 | 118 | 87.05 | <.0001 |
| *NAME*TREAT*TIME* | CODISCO |  | 8 | 1 | 118 | 86.41 | <.0001 |
| *NAME*TREAT*TIME* | CRAZI |  | 1 | 1 | 118 | 0.50 | 0.4800 |
| *NAME*TREAT*TIME* | CRAZI |  | 2 | 1 | 118 | 6.04 | 0.0154 |
| *NAME*TREAT*TIME* | CRAZI |  | 3 | 1 | 118 | 9.82 | 0.0022 |
| *NAME*TREAT*TIME* | CRAZI |  | 4 | 1 | 118 | 22.39 | <.0001 |
| *NAME*TREAT*TIME* | CRAZI |  | 5 | 1 | 118 | 35.57 | <.0001 |
| *NAME*TREAT*TIME* | CRAZI |  | 6 | 1 | 118 | 43.79 | <.0001 |
| *NAME*TREAT*TIME* | CRAZI |  | 7 | 1 | 118 | 49.00 | <.0001 |
| *NAME*TREAT*TIME* | CRAZI |  | 8 | 1 | 118 | 48.18 | <.0001 |
| *NAME*TREAT*TIME* | FERGUS |  | 1 | 1 | 118 | 5.55 | 0.0202 |
| *NAME*TREAT*TIME* | FERGUS |  | 2 | 1 | 118 | 36.93 | <.0001 |
| *NAME*TREAT*TIME* | FERGUS |  | 3 | 1 | 118 | 53.79 | <.0001 |
| *NAME*TREAT*TIME* | FERGUS |  | 4 | 1 | 118 | 99.35 | <.0001 |
| *NAME*TREAT*TIME* | FERGUS |  | 5 | 1 | 118 | 129.95 | <.0001 |
| *NAME*TREAT*TIME* | FERGUS |  | 6 | 1 | 118 | 130.82 | <.0001 |
| *NAME*TREAT*TIME* | FERGUS |  | 7 | 1 | 118 | 125.56 | <.0001 |
| *NAME*TREAT*TIME* | FERGUS |  | 8 | 1 | 118 | 124.01 | <.0001 |
| *NAME*TREAT*TIME* | HUSKI |  | 1 | 1 | 118 | 1.46 | 0.2293 |
| *NAME*TREAT*TIME* | HUSKI |  | 2 | 1 | 118 | 13.08 | 0.0004 |
| *NAME*TREAT*TIME* | HUSKI |  | 3 | 1 | 118 | 8.33 | 0.0046 |
| *NAME*TREAT*TIME* | HUSKI |  | 4 | 1 | 118 | 28.31 | <.0001 |
| *NAME*TREAT*TIME* | HUSKI |  | 5 | 1 | 118 | 42.15 | <.0001 |
| *NAME*TREAT*TIME* | HUSKI |  | 6 | 1 | 118 | 44.06 | <.0001 |
| *NAME*TREAT*TIME* | HUSKI |  | 7 | 1 | 118 | 45.10 | <.0001 |
| *NAME*TREAT*TIME* | HUSKI |  | 8 | 1 | 118 | 50.25 | <.0001 |
| *NAME*TREAT*TIME* | JUSTINA |  | 1 | 1 | 118 | 1.44 | 0.2320 |
| *NAME*TREAT*TIME* | JUSTINA |  | 2 | 1 | 118 | 25.02 | <.0001 |
| *NAME*TREAT*TIME* | JUSTINA |  | 3 | 1 | 118 | 34.34 | <.0001 |
| *NAME*TREAT*TIME* | JUSTINA |  | 4 | 1 | 118 | 67.69 | <.0001 |
| *NAME*TREAT*TIME* | JUSTINA |  | 5 | 1 | 118 | 90.47 | <.0001 |
| *NAME*TREAT*TIME* | JUSTINA |  | 6 | 1 | 118 | 93.45 | <.0001 |
| *NAME*TREAT*TIME* | JUSTINA |  | 7 | 1 | 118 | 109.52 | <.0001 |
| *NAME*TREAT*TIME* | JUSTINA |  | 8 | 1 | 118 | 129.56 | <.0001 |
| *NAME*TREAT*TIME* | LAKTI |  | 1 | 1 | 118 | 0.08 | 0.7763 |
| *NAME*TREAT*TIME* | LAKTI |  | 2 | 1 | 118 | 9.35 | 0.0028 |
| *NAME*TREAT*TIME* | LAKTI |  | 3 | 1 | 118 | 10.92 | 0.0013 |
| *NAME*TREAT*TIME* | LAKTI |  | 4 | 1 | 118 | 31.49 | <.0001 |
| *NAME*TREAT*TIME* | LAKTI |  | 5 | 1 | 118 | 53.64 | <.0001 |
| *NAME*TREAT*TIME* | LAKTI |  | 6 | 1 | 118 | 52.24 | <.0001 |
| *NAME*TREAT*TIME* | LAKTI |  | 7 | 1 | 118 | 50.00 | <.0001 |
| *NAME*TREAT*TIME* | LAKTI |  | 8 | 1 | 118 | 47.29 | <.0001 |
| *NAME*TREAT*TIME* | P329D60 |  | 1 | 1 | 118 | 1.43 | 0.2344 |
| *NAME*TREAT*TIME* | P329D60 |  | 2 | 1 | 118 | 13.25 | 0.0004 |
| *NAME*TREAT*TIME* | P329D60 |  | 3 | 1 | 118 | 15.92 | 0.0001 |
| *NAME*TREAT*TIME* | P329D60 |  | 4 | 1 | 118 | 35.55 | <.0001 |
| *NAME*TREAT*TIME* | P329D60 |  | 5 | 1 | 118 | 51.37 | <.0001 |
| *NAME*TREAT*TIME* | P329D60 |  | 6 | 1 | 118 | 52.72 | <.0001 |
| *NAME*TREAT*TIME* | P329D60 |  | 7 | 1 | 118 | 61.34 | <.0001 |
| *NAME*TREAT*TIME* | P329D60 |  | 8 | 1 | 118 | 66.83 | <.0001 |
| *NAME*TREAT*TIME* | PICKER |  | 1 | 1 | 118 | 0.00 | 0.9819 |
| *NAME*TREAT*TIME* | PICKER |  | 2 | 1 | 118 | 10.43 | 0.0016 |
| *NAME*TREAT*TIME* | PICKER |  | 3 | 1 | 118 | 16.84 | <.0001 |
| *NAME*TREAT*TIME* | PICKER |  | 4 | 1 | 118 | 31.85 | <.0001 |
| *NAME*TREAT*TIME* | PICKER |  | 5 | 1 | 118 | 42.62 | <.0001 |
| *NAME*TREAT*TIME* | PICKER |  | 6 | 1 | 118 | 45.19 | <.0001 |
| *NAME*TREAT*TIME* | PICKER |  | 7 | 1 | 118 | 45.31 | <.0001 |
| *NAME*TREAT*TIME* | PICKER |  | 8 | 1 | 118 | 42.69 | <.0001 |
| *NAME*TREAT*TIME* | PR29B29 |  | 1 | 1 | 118 | 0.04 | 0.8505 |
| *NAME*TREAT*TIME* | PR29B29 |  | 2 | 1 | 118 | 0.42 | 0.5188 |
| *NAME*TREAT*TIME* | PR29B29 |  | 3 | 1 | 118 | 9.96 | 0.0020 |
| *NAME*TREAT*TIME* | PR29B29 |  | 4 | 1 | 118 | 22.80 | <.0001 |
| *NAME*TREAT*TIME* | PR29B29 |  | 5 | 1 | 118 | 26.96 | <.0001 |
| *NAME*TREAT*TIME* | PR29B29 |  | 6 | 1 | 118 | 29.76 | <.0001 |
| *NAME*TREAT*TIME* | PR29B29 |  | 7 | 1 | 118 | 38.17 | <.0001 |
| *NAME*TREAT*TIME* | PR29B29 |  | 8 | 1 | 118 | 42.67 | <.0001 |
| *NAME*TREAT*TIME* | ALGANS | cold |  | 7 | 118 | 8.68 | <.0001 |
| *NAME*TREAT*TIME* | ALGANS | control |  | 7 | 118 | 38.91 | <.0001 |
| *NAME*TREAT*TIME* | CLARITI | cold |  | 7 | 118 | 15.84 | <.0001 |
| *NAME*TREAT*TIME* | CLARITI | control |  | 7 | 118 | 55.21 | <.0001 |
| *NAME*TREAT*TIME* | CODIFAR | cold |  | 7 | 118 | 16.10 | <.0001 |
| *NAME*TREAT*TIME* | CODIFAR | control |  | 7 | 118 | 64.75 | <.0001 |
| *NAME*TREAT*TIME* | CODISCO | cold |  | 7 | 118 | 15.50 | <.0001 |
| *NAME*TREAT*TIME* | CODISCO | control |  | 7 | 118 | 40.43 | <.0001 |
| *NAME*TREAT*TIME* | CRAZI | cold |  | 7 | 118 | 16.17 | <.0001 |
| *NAME*TREAT*TIME* | CRAZI | control |  | 7 | 118 | 52.29 | <.0001 |
| *NAME*TREAT*TIME* | FERGUS | cold |  | 7 | 118 | 14.88 | <.0001 |
| *NAME*TREAT*TIME* | FERGUS | control |  | 7 | 118 | 63.92 | <.0001 |
| *NAME*TREAT*TIME* | HUSKI | cold |  | 7 | 118 | 21.70 | <.0001 |
| *NAME*TREAT*TIME* | HUSKI | control |  | 7 | 118 | 58.73 | <.0001 |
| *NAME*TREAT*TIME* | JUSTINA | cold |  | 7 | 118 | 7.00 | <.0001 |
| *NAME*TREAT*TIME* | JUSTINA | control |  | 7 | 118 | 54.80 | <.0001 |
| *NAME*TREAT*TIME* | LAKTI | cold |  | 7 | 118 | 19.49 | <.0001 |
| *NAME*TREAT*TIME* | LAKTI | control |  | 7 | 118 | 68.05 | <.0001 |
| *NAME*TREAT*TIME* | P329D60 | cold |  | 7 | 118 | 15.41 | <.0001 |
| *NAME*TREAT*TIME* | P329D60 | control |  | 7 | 118 | 52.73 | <.0001 |
| *NAME*TREAT*TIME* | PICKER | cold |  | 7 | 118 | 17.47 | <.0001 |
| *NAME*TREAT*TIME* | PICKER | control |  | 7 | 118 | 51.60 | <.0001 |
| *NAME*TREAT*TIME* | PR29B29 | cold |  | 7 | 118 | 22.00 | <.0001 |
| *NAME*TREAT*TIME* | PR29B29 | control |  | 7 | 118 | 53.11 | <.0001 |
| *NAME*TREAT*TIME* |  | cold | 1 | 11 | 118 | 2.64 | 0.0047 |
| *NAME*TREAT*TIME* |  | cold | 2 | 11 | 118 | 4.94 | <.0001 |
| *NAME*TREAT*TIME* |  | cold | 3 | 11 | 118 | 7.41 | <.0001 |
| *NAME*TREAT*TIME* |  | cold | 4 | 11 | 118 | 10.05 | <.0001 |
| *NAME*TREAT*TIME* |  | cold | 5 | 11 | 118 | 11.85 | <.0001 |
| *NAME*TREAT*TIME* |  | cold | 6 | 11 | 118 | 10.97 | <.0001 |
| *NAME*TREAT*TIME* |  | cold | 7 | 11 | 118 | 11.04 | <.0001 |
| *NAME*TREAT*TIME* |  | cold | 8 | 11 | 118 | 10.92 | <.0001 |
| *NAME*TREAT*TIME* |  | control | 1 | 11 | 118 | 1.95 | 0.0399 |
| *NAME*TREAT*TIME* |  | control | 2 | 11 | 118 | 5.19 | <.0001 |
| *NAME*TREAT*TIME* |  | control | 3 | 11 | 118 | 4.42 | <.0001 |
| *NAME*TREAT*TIME* |  | control | 4 | 11 | 118 | 4.28 | <.0001 |
| *NAME*TREAT*TIME* |  | control | 5 | 11 | 118 | 4.54 | <.0001 |
| *NAME*TREAT*TIME* |  | control | 6 | 11 | 118 | 3.34 | 0.0005 |
| *NAME*TREAT*TIME* |  | control | 7 | 11 | 118 | 2.52 | 0.0069 |
| *NAME*TREAT*TIME* |  | control | 8 | 11 | 118 | 1.89 | 0.0476 |

| *Model Information* | |
| --- | --- |
| *Data Set* | WORK.COLD |
| *Dependent Variable* | logshoot |
| *Covariance Structure* | Unstructured |
| *Subject Effect* | REP*NAME*TREAT*EXP |
| *Estimation Method* | REML |
| *Residual Variance Method* | None |
| *Fixed Effects SE Method* | Model-Based |
| *Degrees of Freedom Method* | Between-Within |

| *Class Level Information* | | |
| --- | --- | --- |
| *Class* | *Levels* | *Values* |
| *REP* | 2 | 1 2 |
| *NAME* | 12 | ALGANS CLARITI CODIFAR CODISCO CRAZI FERGUS HUSKI JUSTINA LAKTI P329D60 PICKER PR29B29 |
| *TREAT* | 2 | cold control |
| *EXP* | 3 | 1 2 3 |
| *TIME* | 8 | 1 2 3 4 5 6 7 8 |

| *Dimensions* | |
| --- | --- |
| *Covariance Parameters* | 36 |
| *Columns in X* | 354 |
| *Columns in Z* | 0 |
| *Subjects* | 144 |
| *Max Obs Per Subject* | 8 |

| *Number of Observations* | |
| --- | --- |
| *Number of Observations Read* | 1152 |
| *Number of Observations Used* | 1152 |
| *Number of Observations Not Used* | 0 |

| *Iteration History* | | | |
| --- | --- | --- | --- |
| *Iteration* | *Evaluations* | *-2 Res Log Like* | *Criterion* |
| *0* | 1 | 590.09044887 |  |
| *1* | 2 | -661.25426832 | 0.00026940 |
| *2* | 1 | -661.60673864 | 0.00000510 |
| *3* | 1 | -661.61300458 | 0.00000000 |

| Convergence criteria met. |
| --- |

| *Estimated R Correlation Matrix for REP*NAME*TREAT*EXP 1 ALGANS cold 1* | | | | | | | | |
| --- | --- | --- | --- | --- | --- | --- | --- | --- |
| *Row* | *Col1* | *Col2* | *Col3* | *Col4* | *Col5* | *Col6* | *Col7* | *Col8* |
| *1* | 1.0000 | 0.3693 | 0.3308 | 0.1593 | 0.1027 | 0.09491 | 0.1390 | 0.05840 |
| *2* | 0.3693 | 1.0000 | 0.7658 | 0.6194 | 0.4914 | 0.4406 | 0.3790 | 0.3628 |
| *3* | 0.3308 | 0.7658 | 1.0000 | 0.8095 | 0.6710 | 0.6023 | 0.5836 | 0.5011 |
| *4* | 0.1593 | 0.6194 | 0.8095 | 1.0000 | 0.8538 | 0.7898 | 0.7102 | 0.6393 |
| *5* | 0.1027 | 0.4914 | 0.6710 | 0.8538 | 1.0000 | 0.9283 | 0.8616 | 0.7533 |
| *6* | 0.09491 | 0.4406 | 0.6023 | 0.7898 | 0.9283 | 1.0000 | 0.9537 | 0.8722 |
| *7* | 0.1390 | 0.3790 | 0.5836 | 0.7102 | 0.8616 | 0.9537 | 1.0000 | 0.9126 |
| *8* | 0.05840 | 0.3628 | 0.5011 | 0.6393 | 0.7533 | 0.8722 | 0.9126 | 1.0000 |

| *Covariance Parameter Estimates* | | |
| --- | --- | --- |
| *Cov Parm* | *Subject* | *Estimate* |
| *UN(1,1)* | REP*NAME*TREAT*EXP | 0.02368 |
| *UN(2,1)* | REP*NAME*TREAT*EXP | 0.01373 |
| *UN(2,2)* | REP*NAME*TREAT*EXP | 0.05840 |
| *UN(3,1)* | REP*NAME*TREAT*EXP | 0.01562 |
| *UN(3,2)* | REP*NAME*TREAT*EXP | 0.05678 |
| *UN(3,3)* | REP*NAME*TREAT*EXP | 0.09412 |
| *UN(4,1)* | REP*NAME*TREAT*EXP | 0.007566 |
| *UN(4,2)* | REP*NAME*TREAT*EXP | 0.04620 |
| *UN(4,3)* | REP*NAME*TREAT*EXP | 0.07665 |
| *UN(4,4)* | REP*NAME*TREAT*EXP | 0.09526 |
| *UN(5,1)* | REP*NAME*TREAT*EXP | 0.004374 |
| *UN(5,2)* | REP*NAME*TREAT*EXP | 0.03286 |
| *UN(5,3)* | REP*NAME*TREAT*EXP | 0.05697 |
| *UN(5,4)* | REP*NAME*TREAT*EXP | 0.07292 |
| *UN(5,5)* | REP*NAME*TREAT*EXP | 0.07657 |
| *UN(6,1)* | REP*NAME*TREAT*EXP | 0.004164 |
| *UN(6,2)* | REP*NAME*TREAT*EXP | 0.03036 |
| *UN(6,3)* | REP*NAME*TREAT*EXP | 0.05268 |
| *UN(6,4)* | REP*NAME*TREAT*EXP | 0.06950 |
| *UN(6,5)* | REP*NAME*TREAT*EXP | 0.07324 |
| *UN(6,6)* | REP*NAME*TREAT*EXP | 0.08129 |
| *UN(7,1)* | REP*NAME*TREAT*EXP | 0.006400 |
| *UN(7,2)* | REP*NAME*TREAT*EXP | 0.02741 |
| *UN(7,3)* | REP*NAME*TREAT*EXP | 0.05359 |
| *UN(7,4)* | REP*NAME*TREAT*EXP | 0.06561 |
| *UN(7,5)* | REP*NAME*TREAT*EXP | 0.07136 |
| *UN(7,6)* | REP*NAME*TREAT*EXP | 0.08138 |
| *UN(7,7)* | REP*NAME*TREAT*EXP | 0.08958 |
| *UN(8,1)* | REP*NAME*TREAT*EXP | 0.002781 |
| *UN(8,2)* | REP*NAME*TREAT*EXP | 0.02713 |
| *UN(8,3)* | REP*NAME*TREAT*EXP | 0.04758 |
| *UN(8,4)* | REP*NAME*TREAT*EXP | 0.06107 |
| *UN(8,5)* | REP*NAME*TREAT*EXP | 0.06451 |
| *UN(8,6)* | REP*NAME*TREAT*EXP | 0.07697 |
| *UN(8,7)* | REP*NAME*TREAT*EXP | 0.08454 |
| *UN(8,8)* | REP*NAME*TREAT*EXP | 0.09579 |

| *Fit Statistics* | |
| --- | --- |
| *-2 Res Log Likelihood* | -661.6 |
| *AIC (smaller is better)* | -589.6 |
| *AICC (smaller is better)* | -586.7 |
| *BIC (smaller is better)* | -482.7 |

| *Null Model Likelihood Ratio Test* | | |
| --- | --- | --- |
| *DF* | *Chi-Square* | *Pr > ChiSq* |
| 35 | 1251.70 | <.0001 |

| *Type 3 Tests of Fixed Effects* | | | | |
| --- | --- | --- | --- | --- |
| *Effect* | *Num DF* | *Den DF* | *F Value* | *Pr > F* |
| *EXP* | 2 | 118 | 3.24 | 0.0426 |
| *NAME* | 11 | 118 | 5.52 | <.0001 |
| *TREAT* | 1 | 118 | 446.64 | <.0001 |
| *NAME*TREAT* | 11 | 118 | 4.76 | <.0001 |
| *TIME* | 7 | 118 | 491.43 | <.0001 |
| *NAME*TIME* | 77 | 118 | 3.07 | <.0001 |
| *TREAT*TIME* | 7 | 118 | 88.93 | <.0001 |
| *NAME*TREAT*TIME* | 77 | 118 | 2.83 | <.0001 |

| *Least Squares Means* | | | | | | | | |
| --- | --- | --- | --- | --- | --- | --- | --- | --- |
| *Effect* | *NAME* | *TREAT* | *TIME* | *Estimate* | *Standard Error* | *DF* | *t Value* | *Pr > \|t\|* |
| *NAME*TREAT*TIME* | ALGANS | cold | 1 | 0.04458 | 0.06282 | 118 | 0.71 | 0.4793 |
| *NAME*TREAT*TIME* | ALGANS | cold | 2 | 0.1008 | 0.09865 | 118 | 1.02 | 0.3092 |
| *NAME*TREAT*TIME* | ALGANS | cold | 3 | 0.3119 | 0.1252 | 118 | 2.49 | 0.0142 |
| *NAME*TREAT*TIME* | ALGANS | cold | 4 | 0.4014 | 0.1260 | 118 | 3.19 | 0.0018 |
| *NAME*TREAT*TIME* | ALGANS | cold | 5 | 0.4498 | 0.1130 | 118 | 3.98 | 0.0001 |
| *NAME*TREAT*TIME* | ALGANS | cold | 6 | 0.6539 | 0.1164 | 118 | 5.62 | <.0001 |
| *NAME*TREAT*TIME* | ALGANS | cold | 7 | 0.7340 | 0.1222 | 118 | 6.01 | <.0001 |
| *NAME*TREAT*TIME* | ALGANS | cold | 8 | 0.8910 | 0.1263 | 118 | 7.05 | <.0001 |
| *NAME*TREAT*TIME* | ALGANS | control | 1 | 0.2339 | 0.06282 | 118 | 3.72 | 0.0003 |
| *NAME*TREAT*TIME* | ALGANS | control | 2 | 0.4909 | 0.09865 | 118 | 4.98 | <.0001 |
| *NAME*TREAT*TIME* | ALGANS | control | 3 | 1.1960 | 0.1252 | 118 | 9.55 | <.0001 |
| *NAME*TREAT*TIME* | ALGANS | control | 4 | 1.4857 | 0.1260 | 118 | 11.79 | <.0001 |
| *NAME*TREAT*TIME* | ALGANS | control | 5 | 1.7017 | 0.1130 | 118 | 15.06 | <.0001 |
| *NAME*TREAT*TIME* | ALGANS | control | 6 | 1.9286 | 0.1164 | 118 | 16.57 | <.0001 |
| *NAME*TREAT*TIME* | ALGANS | control | 7 | 2.1247 | 0.1222 | 118 | 17.39 | <.0001 |
| *NAME*TREAT*TIME* | ALGANS | control | 8 | 2.3334 | 0.1263 | 118 | 18.47 | <.0001 |
| *NAME*TREAT*TIME* | CLARITI | cold | 1 | 0.1259 | 0.06282 | 118 | 2.00 | 0.0474 |
| *NAME*TREAT*TIME* | CLARITI | cold | 2 | 0.2035 | 0.09865 | 118 | 2.06 | 0.0413 |
| *NAME*TREAT*TIME* | CLARITI | cold | 3 | 0.3397 | 0.1252 | 118 | 2.71 | 0.0077 |
| *NAME*TREAT*TIME* | CLARITI | cold | 4 | 0.6565 | 0.1260 | 118 | 5.21 | <.0001 |
| *NAME*TREAT*TIME* | CLARITI | cold | 5 | 0.7935 | 0.1130 | 118 | 7.02 | <.0001 |
| *NAME*TREAT*TIME* | CLARITI | cold | 6 | 0.9363 | 0.1164 | 118 | 8.04 | <.0001 |
| *NAME*TREAT*TIME* | CLARITI | cold | 7 | 1.0336 | 0.1222 | 118 | 8.46 | <.0001 |
| *NAME*TREAT*TIME* | CLARITI | cold | 8 | 1.2066 | 0.1263 | 118 | 9.55 | <.0001 |
| *NAME*TREAT*TIME* | CLARITI | control | 1 | 0.06683 | 0.06282 | 118 | 1.06 | 0.2896 |
| *NAME*TREAT*TIME* | CLARITI | control | 2 | 0.7893 | 0.09865 | 118 | 8.00 | <.0001 |
| *NAME*TREAT*TIME* | CLARITI | control | 3 | 1.0675 | 0.1252 | 118 | 8.52 | <.0001 |
| *NAME*TREAT*TIME* | CLARITI | control | 4 | 1.4646 | 0.1260 | 118 | 11.62 | <.0001 |
| *NAME*TREAT*TIME* | CLARITI | control | 5 | 1.7040 | 0.1130 | 118 | 15.08 | <.0001 |
| *NAME*TREAT*TIME* | CLARITI | control | 6 | 1.8675 | 0.1164 | 118 | 16.04 | <.0001 |
| *NAME*TREAT*TIME* | CLARITI | control | 7 | 2.1038 | 0.1222 | 118 | 17.22 | <.0001 |
| *NAME*TREAT*TIME* | CLARITI | control | 8 | 2.2773 | 0.1263 | 118 | 18.02 | <.0001 |
| *NAME*TREAT*TIME* | CODIFAR | cold | 1 | 0.03466 | 0.06282 | 118 | 0.55 | 0.5821 |
| *NAME*TREAT*TIME* | CODIFAR | cold | 2 | 0.2034 | 0.09865 | 118 | 2.06 | 0.0414 |
| *NAME*TREAT*TIME* | CODIFAR | cold | 3 | 0.3743 | 0.1252 | 118 | 2.99 | 0.0034 |
| *NAME*TREAT*TIME* | CODIFAR | cold | 4 | 0.7452 | 0.1260 | 118 | 5.91 | <.0001 |
| *NAME*TREAT*TIME* | CODIFAR | cold | 5 | 0.9873 | 0.1130 | 118 | 8.74 | <.0001 |
| *NAME*TREAT*TIME* | CODIFAR | cold | 6 | 1.0657 | 0.1164 | 118 | 9.16 | <.0001 |
| *NAME*TREAT*TIME* | CODIFAR | cold | 7 | 1.1236 | 0.1222 | 118 | 9.20 | <.0001 |
| *NAME*TREAT*TIME* | CODIFAR | cold | 8 | 1.2352 | 0.1263 | 118 | 9.78 | <.0001 |
| *NAME*TREAT*TIME* | CODIFAR | control | 1 | -174E-17 | 0.06282 | 118 | -0.00 | 1.0000 |
| *NAME*TREAT*TIME* | CODIFAR | control | 2 | 0.3242 | 0.09865 | 118 | 3.29 | 0.0013 |
| *NAME*TREAT*TIME* | CODIFAR | control | 3 | 0.3641 | 0.1252 | 118 | 2.91 | 0.0044 |
| *NAME*TREAT*TIME* | CODIFAR | control | 4 | 0.7754 | 0.1260 | 118 | 6.15 | <.0001 |
| *NAME*TREAT*TIME* | CODIFAR | control | 5 | 1.2717 | 0.1130 | 118 | 11.26 | <.0001 |
| *NAME*TREAT*TIME* | CODIFAR | control | 6 | 1.7003 | 0.1164 | 118 | 14.61 | <.0001 |
| *NAME*TREAT*TIME* | CODIFAR | control | 7 | 2.0872 | 0.1222 | 118 | 17.08 | <.0001 |
| *NAME*TREAT*TIME* | CODIFAR | control | 8 | 2.3549 | 0.1263 | 118 | 18.64 | <.0001 |
| *NAME*TREAT*TIME* | CODISCO | cold | 1 | 0.06617 | 0.06282 | 118 | 1.05 | 0.2944 |
| *NAME*TREAT*TIME* | CODISCO | cold | 2 | 0.1709 | 0.09865 | 118 | 1.73 | 0.0858 |
| *NAME*TREAT*TIME* | CODISCO | cold | 3 | 0.2179 | 0.1252 | 118 | 1.74 | 0.0846 |
| *NAME*TREAT*TIME* | CODISCO | cold | 4 | 0.3079 | 0.1260 | 118 | 2.44 | 0.0160 |
| *NAME*TREAT*TIME* | CODISCO | cold | 5 | 0.4430 | 0.1130 | 118 | 3.92 | 0.0001 |
| *NAME*TREAT*TIME* | CODISCO | cold | 6 | 0.5447 | 0.1164 | 118 | 4.68 | <.0001 |
| *NAME*TREAT*TIME* | CODISCO | cold | 7 | 0.6006 | 0.1222 | 118 | 4.92 | <.0001 |
| *NAME*TREAT*TIME* | CODISCO | cold | 8 | 0.7814 | 0.1263 | 118 | 6.18 | <.0001 |
| *NAME*TREAT*TIME* | CODISCO | control | 1 | 0.2525 | 0.06282 | 118 | 4.02 | 0.0001 |
| *NAME*TREAT*TIME* | CODISCO | control | 2 | 0.7927 | 0.09865 | 118 | 8.04 | <.0001 |
| *NAME*TREAT*TIME* | CODISCO | control | 3 | 1.1029 | 0.1252 | 118 | 8.81 | <.0001 |
| *NAME*TREAT*TIME* | CODISCO | control | 4 | 1.3937 | 0.1260 | 118 | 11.06 | <.0001 |
| *NAME*TREAT*TIME* | CODISCO | control | 5 | 1.5894 | 0.1130 | 118 | 14.07 | <.0001 |
| *NAME*TREAT*TIME* | CODISCO | control | 6 | 1.7238 | 0.1164 | 118 | 14.81 | <.0001 |
| *NAME*TREAT*TIME* | CODISCO | control | 7 | 1.8750 | 0.1222 | 118 | 15.35 | <.0001 |
| *NAME*TREAT*TIME* | CODISCO | control | 8 | 2.0834 | 0.1263 | 118 | 16.49 | <.0001 |
| *NAME*TREAT*TIME* | CRAZI | cold | 1 | 0.4406 | 0.06282 | 118 | 7.01 | <.0001 |
| *NAME*TREAT*TIME* | CRAZI | cold | 2 | 0.7552 | 0.09865 | 118 | 7.66 | <.0001 |
| *NAME*TREAT*TIME* | CRAZI | cold | 3 | 1.0172 | 0.1252 | 118 | 8.12 | <.0001 |
| *NAME*TREAT*TIME* | CRAZI | cold | 4 | 1.1450 | 0.1260 | 118 | 9.09 | <.0001 |
| *NAME*TREAT*TIME* | CRAZI | cold | 5 | 1.1988 | 0.1130 | 118 | 10.61 | <.0001 |
| *NAME*TREAT*TIME* | CRAZI | cold | 6 | 1.2572 | 0.1164 | 118 | 10.80 | <.0001 |
| *NAME*TREAT*TIME* | CRAZI | cold | 7 | 1.3305 | 0.1222 | 118 | 10.89 | <.0001 |
| *NAME*TREAT*TIME* | CRAZI | cold | 8 | 1.4315 | 0.1263 | 118 | 11.33 | <.0001 |
| *NAME*TREAT*TIME* | CRAZI | control | 1 | 0.4011 | 0.06282 | 118 | 6.39 | <.0001 |
| *NAME*TREAT*TIME* | CRAZI | control | 2 | 0.7821 | 0.09865 | 118 | 7.93 | <.0001 |
| *NAME*TREAT*TIME* | CRAZI | control | 3 | 0.9831 | 0.1252 | 118 | 7.85 | <.0001 |
| *NAME*TREAT*TIME* | CRAZI | control | 4 | 1.4125 | 0.1260 | 118 | 11.21 | <.0001 |
| *NAME*TREAT*TIME* | CRAZI | control | 5 | 1.7670 | 0.1130 | 118 | 15.64 | <.0001 |
| *NAME*TREAT*TIME* | CRAZI | control | 6 | 1.9609 | 0.1164 | 118 | 16.85 | <.0001 |
| *NAME*TREAT*TIME* | CRAZI | control | 7 | 2.1594 | 0.1222 | 118 | 17.67 | <.0001 |
| *NAME*TREAT*TIME* | CRAZI | control | 8 | 2.3631 | 0.1263 | 118 | 18.70 | <.0001 |
| *NAME*TREAT*TIME* | FERGUS | cold | 1 | 0.03450 | 0.06282 | 118 | 0.55 | 0.5839 |
| *NAME*TREAT*TIME* | FERGUS | cold | 2 | 0.07377 | 0.09865 | 118 | 0.75 | 0.4561 |
| *NAME*TREAT*TIME* | FERGUS | cold | 3 | 0.05608 | 0.1252 | 118 | 0.45 | 0.6552 |
| *NAME*TREAT*TIME* | FERGUS | cold | 4 | 0.1796 | 0.1260 | 118 | 1.43 | 0.1567 |
| *NAME*TREAT*TIME* | FERGUS | cold | 5 | 0.3625 | 0.1130 | 118 | 3.21 | 0.0017 |
| *NAME*TREAT*TIME* | FERGUS | cold | 6 | 0.4740 | 0.1164 | 118 | 4.07 | <.0001 |
| *NAME*TREAT*TIME* | FERGUS | cold | 7 | 0.5315 | 0.1222 | 118 | 4.35 | <.0001 |
| *NAME*TREAT*TIME* | FERGUS | cold | 8 | 0.7962 | 0.1263 | 118 | 6.30 | <.0001 |
| *NAME*TREAT*TIME* | FERGUS | control | 1 | 0.03155 | 0.06282 | 118 | 0.50 | 0.6165 |
| *NAME*TREAT*TIME* | FERGUS | control | 2 | 0.6925 | 0.09865 | 118 | 7.02 | <.0001 |
| *NAME*TREAT*TIME* | FERGUS | control | 3 | 1.0035 | 0.1252 | 118 | 8.01 | <.0001 |
| *NAME*TREAT*TIME* | FERGUS | control | 4 | 1.7515 | 0.1260 | 118 | 13.90 | <.0001 |
| *NAME*TREAT*TIME* | FERGUS | control | 5 | 2.1201 | 0.1130 | 118 | 18.77 | <.0001 |
| *NAME*TREAT*TIME* | FERGUS | control | 6 | 2.3738 | 0.1164 | 118 | 20.39 | <.0001 |
| *NAME*TREAT*TIME* | FERGUS | control | 7 | 2.5901 | 0.1222 | 118 | 21.20 | <.0001 |
| *NAME*TREAT*TIME* | FERGUS | control | 8 | 2.7998 | 0.1263 | 118 | 22.16 | <.0001 |
| *NAME*TREAT*TIME* | HUSKI | cold | 1 | 0.03165 | 0.06282 | 118 | 0.50 | 0.6154 |
| *NAME*TREAT*TIME* | HUSKI | cold | 2 | 0.1033 | 0.09865 | 118 | 1.05 | 0.2973 |
| *NAME*TREAT*TIME* | HUSKI | cold | 3 | 0.3933 | 0.1252 | 118 | 3.14 | 0.0021 |
| *NAME*TREAT*TIME* | HUSKI | cold | 4 | 0.4738 | 0.1260 | 118 | 3.76 | 0.0003 |
| *NAME*TREAT*TIME* | HUSKI | cold | 5 | 0.6197 | 0.1130 | 118 | 5.49 | <.0001 |
| *NAME*TREAT*TIME* | HUSKI | cold | 6 | 0.6983 | 0.1164 | 118 | 6.00 | <.0001 |
| *NAME*TREAT*TIME* | HUSKI | cold | 7 | 0.7651 | 0.1222 | 118 | 6.26 | <.0001 |
| *NAME*TREAT*TIME* | HUSKI | cold | 8 | 1.1360 | 0.1263 | 118 | 8.99 | <.0001 |
| *NAME*TREAT*TIME* | HUSKI | control | 1 | 0.09491 | 0.06282 | 118 | 1.51 | 0.1335 |
| *NAME*TREAT*TIME* | HUSKI | control | 2 | 0.6101 | 0.09865 | 118 | 6.18 | <.0001 |
| *NAME*TREAT*TIME* | HUSKI | control | 3 | 0.8781 | 0.1252 | 118 | 7.01 | <.0001 |
| *NAME*TREAT*TIME* | HUSKI | control | 4 | 1.3141 | 0.1260 | 118 | 10.43 | <.0001 |
| *NAME*TREAT*TIME* | HUSKI | control | 5 | 1.6582 | 0.1130 | 118 | 14.68 | <.0001 |
| *NAME*TREAT*TIME* | HUSKI | control | 6 | 1.8935 | 0.1164 | 118 | 16.27 | <.0001 |
| *NAME*TREAT*TIME* | HUSKI | control | 7 | 2.0973 | 0.1222 | 118 | 17.16 | <.0001 |
| *NAME*TREAT*TIME* | HUSKI | control | 8 | 2.3709 | 0.1263 | 118 | 18.76 | <.0001 |
| *NAME*TREAT*TIME* | JUSTINA | cold | 1 | 0.05002 | 0.06282 | 118 | 0.80 | 0.4275 |
| *NAME*TREAT*TIME* | JUSTINA | cold | 2 | 0.01889 | 0.09865 | 118 | 0.19 | 0.8485 |
| *NAME*TREAT*TIME* | JUSTINA | cold | 3 | 0.2114 | 0.1252 | 118 | 1.69 | 0.0940 |
| *NAME*TREAT*TIME* | JUSTINA | cold | 4 | 0.2190 | 0.1260 | 118 | 1.74 | 0.0848 |
| *NAME*TREAT*TIME* | JUSTINA | cold | 5 | 0.2820 | 0.1130 | 118 | 2.50 | 0.0139 |
| *NAME*TREAT*TIME* | JUSTINA | cold | 6 | 0.2990 | 0.1164 | 118 | 2.57 | 0.0114 |
| *NAME*TREAT*TIME* | JUSTINA | cold | 7 | 0.4171 | 0.1222 | 118 | 3.41 | 0.0009 |
| *NAME*TREAT*TIME* | JUSTINA | cold | 8 | 0.4723 | 0.1263 | 118 | 3.74 | 0.0003 |
| *NAME*TREAT*TIME* | JUSTINA | control | 1 | 0.1919 | 0.06282 | 118 | 3.06 | 0.0028 |
| *NAME*TREAT*TIME* | JUSTINA | control | 2 | 0.1328 | 0.09865 | 118 | 1.35 | 0.1808 |
| *NAME*TREAT*TIME* | JUSTINA | control | 3 | 0.6762 | 0.1252 | 118 | 5.40 | <.0001 |
| *NAME*TREAT*TIME* | JUSTINA | control | 4 | 0.9988 | 0.1260 | 118 | 7.93 | <.0001 |
| *NAME*TREAT*TIME* | JUSTINA | control | 5 | 1.2325 | 0.1130 | 118 | 10.91 | <.0001 |
| *NAME*TREAT*TIME* | JUSTINA | control | 6 | 1.4625 | 0.1164 | 118 | 12.56 | <.0001 |
| *NAME*TREAT*TIME* | JUSTINA | control | 7 | 1.7048 | 0.1222 | 118 | 13.95 | <.0001 |
| *NAME*TREAT*TIME* | JUSTINA | control | 8 | 2.0504 | 0.1263 | 118 | 16.23 | <.0001 |
| *NAME*TREAT*TIME* | LAKTI | cold | 1 | 0.01889 | 0.06282 | 118 | 0.30 | 0.7642 |
| *NAME*TREAT*TIME* | LAKTI | cold | 2 | 0.1519 | 0.09865 | 118 | 1.54 | 0.1262 |
| *NAME*TREAT*TIME* | LAKTI | cold | 3 | 0.2057 | 0.1252 | 118 | 1.64 | 0.1032 |
| *NAME*TREAT*TIME* | LAKTI | cold | 4 | 0.3319 | 0.1260 | 118 | 2.63 | 0.0096 |
| *NAME*TREAT*TIME* | LAKTI | cold | 5 | 0.3947 | 0.1130 | 118 | 3.49 | 0.0007 |
| *NAME*TREAT*TIME* | LAKTI | cold | 6 | 0.5702 | 0.1164 | 118 | 4.90 | <.0001 |
| *NAME*TREAT*TIME* | LAKTI | cold | 7 | 0.6547 | 0.1222 | 118 | 5.36 | <.0001 |
| *NAME*TREAT*TIME* | LAKTI | cold | 8 | 0.9752 | 0.1263 | 118 | 7.72 | <.0001 |
| *NAME*TREAT*TIME* | LAKTI | control | 1 | 0.04511 | 0.06282 | 118 | 0.72 | 0.4742 |
| *NAME*TREAT*TIME* | LAKTI | control | 2 | 0.3124 | 0.09865 | 118 | 3.17 | 0.0020 |
| *NAME*TREAT*TIME* | LAKTI | control | 3 | 0.5986 | 0.1252 | 118 | 4.78 | <.0001 |
| *NAME*TREAT*TIME* | LAKTI | control | 4 | 1.2252 | 0.1260 | 118 | 9.72 | <.0001 |
| *NAME*TREAT*TIME* | LAKTI | control | 5 | 1.7668 | 0.1130 | 118 | 15.64 | <.0001 |
| *NAME*TREAT*TIME* | LAKTI | control | 6 | 2.1450 | 0.1164 | 118 | 18.43 | <.0001 |
| *NAME*TREAT*TIME* | LAKTI | control | 7 | 2.4523 | 0.1222 | 118 | 20.07 | <.0001 |
| *NAME*TREAT*TIME* | LAKTI | control | 8 | 2.6631 | 0.1263 | 118 | 21.08 | <.0001 |
| *NAME*TREAT*TIME* | P329D60 | cold | 1 | -333E-18 | 0.06282 | 118 | -0.00 | 1.0000 |
| *NAME*TREAT*TIME* | P329D60 | cold | 2 | -333E-18 | 0.09865 | 118 | -0.00 | 1.0000 |
| *NAME*TREAT*TIME* | P329D60 | cold | 3 | 0.3346 | 0.1252 | 118 | 2.67 | 0.0086 |
| *NAME*TREAT*TIME* | P329D60 | cold | 4 | 0.3788 | 0.1260 | 118 | 3.01 | 0.0032 |
| *NAME*TREAT*TIME* | P329D60 | cold | 5 | 0.4581 | 0.1130 | 118 | 4.06 | <.0001 |
| *NAME*TREAT*TIME* | P329D60 | cold | 6 | 0.5626 | 0.1164 | 118 | 4.83 | <.0001 |
| *NAME*TREAT*TIME* | P329D60 | cold | 7 | 0.7095 | 0.1222 | 118 | 5.81 | <.0001 |
| *NAME*TREAT*TIME* | P329D60 | cold | 8 | 0.7615 | 0.1263 | 118 | 6.03 | <.0001 |
| *NAME*TREAT*TIME* | P329D60 | control | 1 | -666E-18 | 0.06282 | 118 | -0.00 | 1.0000 |
| *NAME*TREAT*TIME* | P329D60 | control | 2 | 0.1910 | 0.09865 | 118 | 1.94 | 0.0553 |
| *NAME*TREAT*TIME* | P329D60 | control | 3 | 0.6381 | 0.1252 | 118 | 5.09 | <.0001 |
| *NAME*TREAT*TIME* | P329D60 | control | 4 | 1.0864 | 0.1260 | 118 | 8.62 | <.0001 |
| *NAME*TREAT*TIME* | P329D60 | control | 5 | 1.5640 | 0.1130 | 118 | 13.84 | <.0001 |
| *NAME*TREAT*TIME* | P329D60 | control | 6 | 1.8184 | 0.1164 | 118 | 15.62 | <.0001 |
| *NAME*TREAT*TIME* | P329D60 | control | 7 | 2.1497 | 0.1222 | 118 | 17.59 | <.0001 |
| *NAME*TREAT*TIME* | P329D60 | control | 8 | 2.3900 | 0.1263 | 118 | 18.92 | <.0001 |
| *NAME*TREAT*TIME* | PICKER | cold | 1 | 0.09411 | 0.06282 | 118 | 1.50 | 0.1368 |
| *NAME*TREAT*TIME* | PICKER | cold | 2 | 0.09783 | 0.09865 | 118 | 0.99 | 0.3234 |
| *NAME*TREAT*TIME* | PICKER | cold | 3 | 0.6649 | 0.1252 | 118 | 5.31 | <.0001 |
| *NAME*TREAT*TIME* | PICKER | cold | 4 | 0.7623 | 0.1260 | 118 | 6.05 | <.0001 |
| *NAME*TREAT*TIME* | PICKER | cold | 5 | 0.9946 | 0.1130 | 118 | 8.80 | <.0001 |
| *NAME*TREAT*TIME* | PICKER | cold | 6 | 1.0472 | 0.1164 | 118 | 9.00 | <.0001 |
| *NAME*TREAT*TIME* | PICKER | cold | 7 | 1.1851 | 0.1222 | 118 | 9.70 | <.0001 |
| *NAME*TREAT*TIME* | PICKER | cold | 8 | 1.3197 | 0.1263 | 118 | 10.45 | <.0001 |
| *NAME*TREAT*TIME* | PICKER | control | 1 | 0.1380 | 0.06282 | 118 | 2.20 | 0.0300 |
| *NAME*TREAT*TIME* | PICKER | control | 2 | 0.6543 | 0.09865 | 118 | 6.63 | <.0001 |
| *NAME*TREAT*TIME* | PICKER | control | 3 | 0.9742 | 0.1252 | 118 | 7.78 | <.0001 |
| *NAME*TREAT*TIME* | PICKER | control | 4 | 1.4928 | 0.1260 | 118 | 11.85 | <.0001 |
| *NAME*TREAT*TIME* | PICKER | control | 5 | 1.7535 | 0.1130 | 118 | 15.52 | <.0001 |
| *NAME*TREAT*TIME* | PICKER | control | 6 | 1.9066 | 0.1164 | 118 | 16.38 | <.0001 |
| *NAME*TREAT*TIME* | PICKER | control | 7 | 2.0862 | 0.1222 | 118 | 17.07 | <.0001 |
| *NAME*TREAT*TIME* | PICKER | control | 8 | 2.2469 | 0.1263 | 118 | 17.78 | <.0001 |
| *NAME*TREAT*TIME* | PR29B29 | cold | 1 | 0.1145 | 0.06282 | 118 | 1.82 | 0.0709 |
| *NAME*TREAT*TIME* | PR29B29 | cold | 2 | 0.2940 | 0.09865 | 118 | 2.98 | 0.0035 |
| *NAME*TREAT*TIME* | PR29B29 | cold | 3 | 0.3855 | 0.1252 | 118 | 3.08 | 0.0026 |
| *NAME*TREAT*TIME* | PR29B29 | cold | 4 | 0.5975 | 0.1260 | 118 | 4.74 | <.0001 |
| *NAME*TREAT*TIME* | PR29B29 | cold | 5 | 0.7791 | 0.1130 | 118 | 6.90 | <.0001 |
| *NAME*TREAT*TIME* | PR29B29 | cold | 6 | 1.0847 | 0.1164 | 118 | 9.32 | <.0001 |
| *NAME*TREAT*TIME* | PR29B29 | cold | 7 | 1.1538 | 0.1222 | 118 | 9.44 | <.0001 |
| *NAME*TREAT*TIME* | PR29B29 | cold | 8 | 1.3108 | 0.1263 | 118 | 10.37 | <.0001 |
| *NAME*TREAT*TIME* | PR29B29 | control | 1 | 0.01128 | 0.06282 | 118 | 0.18 | 0.8579 |
| *NAME*TREAT*TIME* | PR29B29 | control | 2 | 0.3566 | 0.09865 | 118 | 3.61 | 0.0004 |
| *NAME*TREAT*TIME* | PR29B29 | control | 3 | 0.8103 | 0.1252 | 118 | 6.47 | <.0001 |
| *NAME*TREAT*TIME* | PR29B29 | control | 4 | 1.1885 | 0.1260 | 118 | 9.43 | <.0001 |
| *NAME*TREAT*TIME* | PR29B29 | control | 5 | 1.3806 | 0.1130 | 118 | 12.22 | <.0001 |
| *NAME*TREAT*TIME* | PR29B29 | control | 6 | 1.6616 | 0.1164 | 118 | 14.27 | <.0001 |
| *NAME*TREAT*TIME* | PR29B29 | control | 7 | 1.9037 | 0.1222 | 118 | 15.58 | <.0001 |
| *NAME*TREAT*TIME* | PR29B29 | control | 8 | 2.1619 | 0.1263 | 118 | 17.11 | <.0001 |

| *Tests of Effect Slices* | | | | | | | |
| --- | --- | --- | --- | --- | --- | --- | --- |
| *Effect* | *NAME* | *TREAT* | *TIME* | *Num DF* | *Den DF* | *F Value* | *Pr > F* |
| *NAME*TREAT*TIME* | ALGANS |  |  | 15 | 118 | 23.03 | <.0001 |
| *NAME*TREAT*TIME* | CLARITI |  |  | 15 | 118 | 24.47 | <.0001 |
| *NAME*TREAT*TIME* | CODIFAR |  |  | 15 | 118 | 33.03 | <.0001 |
| *NAME*TREAT*TIME* | CODISCO |  |  | 15 | 118 | 16.32 | <.0001 |
| *NAME*TREAT*TIME* | CRAZI |  |  | 15 | 118 | 19.25 | <.0001 |
| *NAME*TREAT*TIME* | FERGUS |  |  | 15 | 118 | 34.74 | <.0001 |
| *NAME*TREAT*TIME* | HUSKI |  |  | 15 | 118 | 27.64 | <.0001 |
| *NAME*TREAT*TIME* | JUSTINA |  |  | 15 | 118 | 19.44 | <.0001 |
| *NAME*TREAT*TIME* | LAKTI |  |  | 15 | 118 | 35.12 | <.0001 |
| *NAME*TREAT*TIME* | P329D60 |  |  | 15 | 118 | 28.95 | <.0001 |
| *NAME*TREAT*TIME* | PICKER |  |  | 15 | 118 | 26.27 | <.0001 |
| *NAME*TREAT*TIME* | PR29B29 |  |  | 15 | 118 | 24.11 | <.0001 |
| *NAME*TREAT*TIME* |  | cold |  | 95 | 118 | 9.43 | <.0001 |
| *NAME*TREAT*TIME* |  | control |  | 95 | 118 | 39.42 | <.0001 |
| *NAME*TREAT*TIME* |  |  | 1 | 23 | 118 | 3.65 | <.0001 |
| *NAME*TREAT*TIME* |  |  | 2 | 23 | 118 | 7.70 | <.0001 |
| *NAME*TREAT*TIME* |  |  | 3 | 23 | 118 | 7.60 | <.0001 |
| *NAME*TREAT*TIME* |  |  | 4 | 23 | 118 | 14.45 | <.0001 |
| *NAME*TREAT*TIME* |  |  | 5 | 23 | 118 | 25.15 | <.0001 |
| *NAME*TREAT*TIME* |  |  | 6 | 23 | 118 | 28.59 | <.0001 |
| *NAME*TREAT*TIME* |  |  | 7 | 23 | 118 | 32.28 | <.0001 |
| *NAME*TREAT*TIME* |  |  | 8 | 23 | 118 | 32.18 | <.0001 |
| *NAME*TREAT*TIME* | ALGANS |  | 1 | 1 | 118 | 4.54 | 0.0351 |
| *NAME*TREAT*TIME* | ALGANS |  | 2 | 1 | 118 | 7.82 | 0.0060 |
| *NAME*TREAT*TIME* | ALGANS |  | 3 | 1 | 118 | 24.91 | <.0001 |
| *NAME*TREAT*TIME* | ALGANS |  | 4 | 1 | 118 | 37.02 | <.0001 |
| *NAME*TREAT*TIME* | ALGANS |  | 5 | 1 | 118 | 61.40 | <.0001 |
| *NAME*TREAT*TIME* | ALGANS |  | 6 | 1 | 118 | 59.96 | <.0001 |
| *NAME*TREAT*TIME* | ALGANS |  | 7 | 1 | 118 | 64.77 | <.0001 |
| *NAME*TREAT*TIME* | ALGANS |  | 8 | 1 | 118 | 65.16 | <.0001 |
| *NAME*TREAT*TIME* | CLARITI |  | 1 | 1 | 118 | 0.44 | 0.5076 |
| *NAME*TREAT*TIME* | CLARITI |  | 2 | 1 | 118 | 17.63 | <.0001 |
| *NAME*TREAT*TIME* | CLARITI |  | 3 | 1 | 118 | 16.88 | <.0001 |
| *NAME*TREAT*TIME* | CLARITI |  | 4 | 1 | 118 | 20.57 | <.0001 |
| *NAME*TREAT*TIME* | CLARITI |  | 5 | 1 | 118 | 32.48 | <.0001 |
| *NAME*TREAT*TIME* | CLARITI |  | 6 | 1 | 118 | 32.01 | <.0001 |
| *NAME*TREAT*TIME* | CLARITI |  | 7 | 1 | 118 | 38.36 | <.0001 |
| *NAME*TREAT*TIME* | CLARITI |  | 8 | 1 | 118 | 35.90 | <.0001 |
| *NAME*TREAT*TIME* | CODIFAR |  | 1 | 1 | 118 | 0.15 | 0.6971 |
| *NAME*TREAT*TIME* | CODIFAR |  | 2 | 1 | 118 | 0.75 | 0.3886 |
| *NAME*TREAT*TIME* | CODIFAR |  | 3 | 1 | 118 | 0.00 | 0.9541 |
| *NAME*TREAT*TIME* | CODIFAR |  | 4 | 1 | 118 | 0.03 | 0.8655 |
| *NAME*TREAT*TIME* | CODIFAR |  | 5 | 1 | 118 | 3.17 | 0.0776 |
| *NAME*TREAT*TIME* | CODIFAR |  | 6 | 1 | 118 | 14.86 | 0.0002 |
| *NAME*TREAT*TIME* | CODIFAR |  | 7 | 1 | 118 | 31.09 | <.0001 |
| *NAME*TREAT*TIME* | CODIFAR |  | 8 | 1 | 118 | 39.27 | <.0001 |
| *NAME*TREAT*TIME* | CODISCO |  | 1 | 1 | 118 | 4.40 | 0.0382 |
| *NAME*TREAT*TIME* | CODISCO |  | 2 | 1 | 118 | 19.86 | <.0001 |
| *NAME*TREAT*TIME* | CODISCO |  | 3 | 1 | 118 | 24.97 | <.0001 |
| *NAME*TREAT*TIME* | CODISCO |  | 4 | 1 | 118 | 37.13 | <.0001 |
| *NAME*TREAT*TIME* | CODISCO |  | 5 | 1 | 118 | 51.49 | <.0001 |
| *NAME*TREAT*TIME* | CODISCO |  | 6 | 1 | 118 | 51.31 | <.0001 |
| *NAME*TREAT*TIME* | CODISCO |  | 7 | 1 | 118 | 54.39 | <.0001 |
| *NAME*TREAT*TIME* | CODISCO |  | 8 | 1 | 118 | 53.09 | <.0001 |
| *NAME*TREAT*TIME* | CRAZI |  | 1 | 1 | 118 | 0.20 | 0.6577 |
| *NAME*TREAT*TIME* | CRAZI |  | 2 | 1 | 118 | 0.04 | 0.8477 |
| *NAME*TREAT*TIME* | CRAZI |  | 3 | 1 | 118 | 0.04 | 0.8475 |
| *NAME*TREAT*TIME* | CRAZI |  | 4 | 1 | 118 | 2.25 | 0.1361 |
| *NAME*TREAT*TIME* | CRAZI |  | 5 | 1 | 118 | 12.65 | 0.0005 |
| *NAME*TREAT*TIME* | CRAZI |  | 6 | 1 | 118 | 18.28 | <.0001 |
| *NAME*TREAT*TIME* | CRAZI |  | 7 | 1 | 118 | 23.01 | <.0001 |
| *NAME*TREAT*TIME* | CRAZI |  | 8 | 1 | 118 | 27.19 | <.0001 |
| *NAME*TREAT*TIME* | FERGUS |  | 1 | 1 | 118 | 0.00 | 0.9735 |
| *NAME*TREAT*TIME* | FERGUS |  | 2 | 1 | 118 | 19.67 | <.0001 |
| *NAME*TREAT*TIME* | FERGUS |  | 3 | 1 | 118 | 28.61 | <.0001 |
| *NAME*TREAT*TIME* | FERGUS |  | 4 | 1 | 118 | 77.81 | <.0001 |
| *NAME*TREAT*TIME* | FERGUS |  | 5 | 1 | 118 | 121.03 | <.0001 |
| *NAME*TREAT*TIME* | FERGUS |  | 6 | 1 | 118 | 133.19 | <.0001 |
| *NAME*TREAT*TIME* | FERGUS |  | 7 | 1 | 118 | 141.92 | <.0001 |
| *NAME*TREAT*TIME* | FERGUS |  | 8 | 1 | 118 | 125.74 | <.0001 |
| *NAME*TREAT*TIME* | HUSKI |  | 1 | 1 | 118 | 0.51 | 0.4778 |
| *NAME*TREAT*TIME* | HUSKI |  | 2 | 1 | 118 | 13.20 | 0.0004 |
| *NAME*TREAT*TIME* | HUSKI |  | 3 | 1 | 118 | 7.49 | 0.0072 |
| *NAME*TREAT*TIME* | HUSKI |  | 4 | 1 | 118 | 22.24 | <.0001 |
| *NAME*TREAT*TIME* | HUSKI |  | 5 | 1 | 118 | 42.26 | <.0001 |
| *NAME*TREAT*TIME* | HUSKI |  | 6 | 1 | 118 | 52.72 | <.0001 |
| *NAME*TREAT*TIME* | HUSKI |  | 7 | 1 | 118 | 59.44 | <.0001 |
| *NAME*TREAT*TIME* | HUSKI |  | 8 | 1 | 118 | 47.76 | <.0001 |
| *NAME*TREAT*TIME* | JUSTINA |  | 1 | 1 | 118 | 2.55 | 0.1129 |
| *NAME*TREAT*TIME* | JUSTINA |  | 2 | 1 | 118 | 0.67 | 0.4158 |
| *NAME*TREAT*TIME* | JUSTINA |  | 3 | 1 | 118 | 6.89 | 0.0098 |
| *NAME*TREAT*TIME* | JUSTINA |  | 4 | 1 | 118 | 19.15 | <.0001 |
| *NAME*TREAT*TIME* | JUSTINA |  | 5 | 1 | 118 | 35.40 | <.0001 |
| *NAME*TREAT*TIME* | JUSTINA |  | 6 | 1 | 118 | 49.96 | <.0001 |
| *NAME*TREAT*TIME* | JUSTINA |  | 7 | 1 | 118 | 55.53 | <.0001 |
| *NAME*TREAT*TIME* | JUSTINA |  | 8 | 1 | 118 | 78.00 | <.0001 |
| *NAME*TREAT*TIME* | LAKTI |  | 1 | 1 | 118 | 0.09 | 0.7684 |
| *NAME*TREAT*TIME* | LAKTI |  | 2 | 1 | 118 | 1.32 | 0.2524 |
| *NAME*TREAT*TIME* | LAKTI |  | 3 | 1 | 118 | 4.92 | 0.0284 |
| *NAME*TREAT*TIME* | LAKTI |  | 4 | 1 | 118 | 25.13 | <.0001 |
| *NAME*TREAT*TIME* | LAKTI |  | 5 | 1 | 118 | 73.76 | <.0001 |
| *NAME*TREAT*TIME* | LAKTI |  | 6 | 1 | 118 | 91.52 | <.0001 |
| *NAME*TREAT*TIME* | LAKTI |  | 7 | 1 | 118 | 108.22 | <.0001 |
| *NAME*TREAT*TIME* | LAKTI |  | 8 | 1 | 118 | 89.23 | <.0001 |
| *NAME*TREAT*TIME* | P329D60 |  | 1 | 1 | 118 | 0.00 | 1.0000 |
| *NAME*TREAT*TIME* | P329D60 |  | 2 | 1 | 118 | 1.87 | 0.1737 |
| *NAME*TREAT*TIME* | P329D60 |  | 3 | 1 | 118 | 2.94 | 0.0892 |
| *NAME*TREAT*TIME* | P329D60 |  | 4 | 1 | 118 | 15.77 | 0.0001 |
| *NAME*TREAT*TIME* | P329D60 |  | 5 | 1 | 118 | 47.91 | <.0001 |
| *NAME*TREAT*TIME* | P329D60 |  | 6 | 1 | 118 | 58.20 | <.0001 |
| *NAME*TREAT*TIME* | P329D60 |  | 7 | 1 | 118 | 69.46 | <.0001 |
| *NAME*TREAT*TIME* | P329D60 |  | 8 | 1 | 118 | 83.05 | <.0001 |
| *NAME*TREAT*TIME* | PICKER |  | 1 | 1 | 118 | 0.24 | 0.6220 |
| *NAME*TREAT*TIME* | PICKER |  | 2 | 1 | 118 | 15.91 | 0.0001 |
| *NAME*TREAT*TIME* | PICKER |  | 3 | 1 | 118 | 3.05 | 0.0834 |
| *NAME*TREAT*TIME* | PICKER |  | 4 | 1 | 118 | 16.80 | <.0001 |
| *NAME*TREAT*TIME* | PICKER |  | 5 | 1 | 118 | 22.57 | <.0001 |
| *NAME*TREAT*TIME* | PICKER |  | 6 | 1 | 118 | 27.26 | <.0001 |
| *NAME*TREAT*TIME* | PICKER |  | 7 | 1 | 118 | 27.19 | <.0001 |
| *NAME*TREAT*TIME* | PICKER |  | 8 | 1 | 118 | 26.93 | <.0001 |
| *NAME*TREAT*TIME* | PR29B29 |  | 1 | 1 | 118 | 1.35 | 0.2477 |
| *NAME*TREAT*TIME* | PR29B29 |  | 2 | 1 | 118 | 0.20 | 0.6546 |
| *NAME*TREAT*TIME* | PR29B29 |  | 3 | 1 | 118 | 5.75 | 0.0180 |
| *NAME*TREAT*TIME* | PR29B29 |  | 4 | 1 | 118 | 11.00 | 0.0012 |
| *NAME*TREAT*TIME* | PR29B29 |  | 5 | 1 | 118 | 14.17 | 0.0003 |
| *NAME*TREAT*TIME* | PR29B29 |  | 6 | 1 | 118 | 12.28 | 0.0006 |
| *NAME*TREAT*TIME* | PR29B29 |  | 7 | 1 | 118 | 18.83 | <.0001 |
| *NAME*TREAT*TIME* | PR29B29 |  | 8 | 1 | 118 | 22.69 | <.0001 |
| *NAME*TREAT*TIME* | ALGANS | cold |  | 7 | 118 | 7.22 | <.0001 |
| *NAME*TREAT*TIME* | ALGANS | control |  | 7 | 118 | 38.32 | <.0001 |
| *NAME*TREAT*TIME* | CLARITI | cold |  | 7 | 118 | 10.34 | <.0001 |
| *NAME*TREAT*TIME* | CLARITI | control |  | 7 | 118 | 41.57 | <.0001 |
| *NAME*TREAT*TIME* | CODIFAR | cold |  | 7 | 118 | 13.00 | <.0001 |
| *NAME*TREAT*TIME* | CODIFAR | control |  | 7 | 118 | 57.58 | <.0001 |
| *NAME*TREAT*TIME* | CODISCO | cold |  | 7 | 118 | 4.85 | <.0001 |
| *NAME*TREAT*TIME* | CODISCO | control |  | 7 | 118 | 26.46 | <.0001 |
| *NAME*TREAT*TIME* | CRAZI | cold |  | 7 | 118 | 7.89 | <.0001 |
| *NAME*TREAT*TIME* | CRAZI | control |  | 7 | 118 | 32.79 | <.0001 |
| *NAME*TREAT*TIME* | FERGUS | cold |  | 7 | 118 | 7.26 | <.0001 |
| *NAME*TREAT*TIME* | FERGUS | control |  | 7 | 118 | 63.72 | <.0001 |
| *NAME*TREAT*TIME* | HUSKI | cold |  | 7 | 118 | 15.48 | <.0001 |
| *NAME*TREAT*TIME* | HUSKI | control |  | 7 | 118 | 41.72 | <.0001 |
| *NAME*TREAT*TIME* | JUSTINA | cold |  | 7 | 118 | 2.93 | 0.0073 |
| *NAME*TREAT*TIME* | JUSTINA | control |  | 7 | 118 | 35.45 | <.0001 |
| *NAME*TREAT*TIME* | LAKTI | cold |  | 7 | 118 | 9.86 | <.0001 |
| *NAME*TREAT*TIME* | LAKTI | control |  | 7 | 118 | 62.93 | <.0001 |
| *NAME*TREAT*TIME* | P329D60 | cold |  | 7 | 118 | 6.67 | <.0001 |
| *NAME*TREAT*TIME* | P329D60 | control |  | 7 | 118 | 53.01 | <.0001 |
| *NAME*TREAT*TIME* | PICKER | cold |  | 7 | 118 | 17.84 | <.0001 |
| *NAME*TREAT*TIME* | PICKER | control |  | 7 | 118 | 36.79 | <.0001 |
| *NAME*TREAT*TIME* | PR29B29 | cold |  | 7 | 118 | 13.90 | <.0001 |
| *NAME*TREAT*TIME* | PR29B29 | control |  | 7 | 118 | 37.73 | <.0001 |
| *NAME*TREAT*TIME* |  | cold | 1 | 11 | 118 | 3.49 | 0.0003 |
| *NAME*TREAT*TIME* |  | cold | 2 | 11 | 118 | 4.06 | <.0001 |
| *NAME*TREAT*TIME* |  | cold | 3 | 11 | 118 | 3.97 | <.0001 |
| *NAME*TREAT*TIME* |  | cold | 4 | 11 | 118 | 4.85 | <.0001 |
| *NAME*TREAT*TIME* |  | cold | 5 | 11 | 118 | 6.94 | <.0001 |
| *NAME*TREAT*TIME* |  | cold | 6 | 11 | 118 | 6.64 | <.0001 |
| *NAME*TREAT*TIME* |  | cold | 7 | 11 | 118 | 5.93 | <.0001 |
| *NAME*TREAT*TIME* |  | cold | 8 | 11 | 118 | 5.30 | <.0001 |
| *NAME*TREAT*TIME* |  | control | 1 | 11 | 118 | 3.97 | <.0001 |
| *NAME*TREAT*TIME* |  | control | 2 | 11 | 118 | 5.96 | <.0001 |
| *NAME*TREAT*TIME* |  | control | 3 | 11 | 118 | 3.85 | <.0001 |
| *NAME*TREAT*TIME* |  | control | 4 | 11 | 118 | 4.32 | <.0001 |
| *NAME*TREAT*TIME* |  | control | 5 | 11 | 118 | 4.69 | <.0001 |
| *NAME*TREAT*TIME* |  | control | 6 | 11 | 118 | 4.06 | <.0001 |
| *NAME*TREAT*TIME* |  | control | 7 | 11 | 118 | 3.75 | 0.0001 |
| *NAME*TREAT*TIME* |  | control | 8 | 11 | 118 | 2.93 | 0.0019 |

| *Model Information* | |
| --- | --- |
| *Data Set* | WORK.COMBINE |
| *Dependent Variable* | ratio |
| *Covariance Structure* | Diagonal |
| *Estimation Method* | REML |
| *Residual Variance Method* | Profile |
| *Fixed Effects SE Method* | Model-Based |
| *Degrees of Freedom Method* | Residual |

| *Class Level Information* | | |
| --- | --- | --- |
| *Class* | *Levels* | *Values* |
| *EXP* | 3 | 1 2 3 |
| *NAME* | 12 | ALGANS CLARITI CODIFAR CODISCO CRAZI FERGUS HUSKI JUSTINA LAKTI P329D60 PICKER PR29B29 |

| *Dimensions* | |
| --- | --- |
| *Covariance Parameters* | 1 |
| *Columns in X* | 16 |
| *Columns in Z* | 0 |
| *Subjects* | 1 |
| *Max Obs Per Subject* | 36 |

| *Number of Observations* | |
| --- | --- |
| *Number of Observations Read* | 36 |
| *Number of Observations Used* | 36 |
| *Number of Observations Not Used* | 0 |

| *Covariance Parameter Estimates* | |
| --- | --- |
| *Cov Parm* | *Estimate* |
| *Residual* | 0.003105 |

| *Fit Statistics* | |
| --- | --- |
| *-2 Res Log Likelihood* | -47.6 |
| *AIC (smaller is better)* | -45.6 |
| *AICC (smaller is better)* | -45.4 |
| *BIC (smaller is better)* | -44.5 |

| *Type 3 Tests of Fixed Effects* | | | | |
| --- | --- | --- | --- | --- |
| *Effect* | *Num DF* | *Den DF* | *F Value* | *Pr > F* |
| *EXP* | 2 | 22 | 61.61 | <.0001 |
| *NAME* | 11 | 22 | 6.33 | 0.0001 |

| *Least Squares Means* | | | | | | |
| --- | --- | --- | --- | --- | --- | --- |
| *Effect* | *NAME* | *Estimate* | *Standard Error* | *DF* | *t Value* | *Pr > \|t\|* |
| *NAME* | ALGANS | 0.1079 | 0.03217 | 22 | 3.35 | 0.0029 |
| *NAME* | CLARITI | 0.2112 | 0.03217 | 22 | 6.56 | <.0001 |
| *NAME* | CODIFAR | 0.3189 | 0.03217 | 22 | 9.91 | <.0001 |
| *NAME* | CODISCO | 0.1533 | 0.03217 | 22 | 4.77 | <.0001 |
| *NAME* | CRAZI | 0.2717 | 0.03217 | 22 | 8.44 | <.0001 |
| *NAME* | FERGUS | 0.1322 | 0.03217 | 22 | 4.11 | 0.0005 |
| *NAME* | HUSKI | 0.2425 | 0.03217 | 22 | 7.54 | <.0001 |
| *NAME* | JUSTINA | 0.09462 | 0.03217 | 22 | 2.94 | 0.0076 |
| *NAME* | LAKTI | 0.3050 | 0.03217 | 22 | 9.48 | <.0001 |
| *NAME* | P329D60 | 0.2119 | 0.03217 | 22 | 6.59 | <.0001 |
| *NAME* | PICKER | 0.2975 | 0.03217 | 22 | 9.25 | <.0001 |
| *NAME* | PR29B29 | 0.2932 | 0.03217 | 22 | 9.11 | <.0001 |

| *Differences of Least Squares Means* | | | | | | | | | |
| --- | --- | --- | --- | --- | --- | --- | --- | --- | --- |
| *Effect* | *NAME* | *_NAME* | *Estimate* | *Standard Error* | *DF* | *t Value* | *Pr > \|t\|* | *Adjustment* | *Adj P* |
| *NAME* | ALGANS | CLARITI | -0.1033 | 0.04550 | 22 | -2.27 | 0.0334 | Tukey | 0.5226 |
| *NAME* | ALGANS | CODIFAR | -0.2110 | 0.04550 | 22 | -4.64 | 0.0001 | Tukey | 0.0055 |
| *NAME* | ALGANS | CODISCO | -0.04541 | 0.04550 | 22 | -1.00 | 0.3291 | Tukey | 0.9961 |
| *NAME* | ALGANS | CRAZI | -0.1638 | 0.04550 | 22 | -3.60 | 0.0016 | Tukey | 0.0542 |
| *NAME* | ALGANS | FERGUS | -0.02430 | 0.04550 | 22 | -0.53 | 0.5987 | Tukey | 1.0000 |
| *NAME* | ALGANS | HUSKI | -0.1346 | 0.04550 | 22 | -2.96 | 0.0073 | Tukey | 0.1873 |
| *NAME* | ALGANS | JUSTINA | 0.01331 | 0.04550 | 22 | 0.29 | 0.7726 | Tukey | 1.0000 |
| *NAME* | ALGANS | LAKTI | -0.1970 | 0.04550 | 22 | -4.33 | 0.0003 | Tukey | 0.0110 |
| *NAME* | ALGANS | P329D60 | -0.1039 | 0.04550 | 22 | -2.28 | 0.0323 | Tukey | 0.5134 |
| *NAME* | ALGANS | PICKER | -0.1896 | 0.04550 | 22 | -4.17 | 0.0004 | Tukey | 0.0159 |
| *NAME* | ALGANS | PR29B29 | -0.1852 | 0.04550 | 22 | -4.07 | 0.0005 | Tukey | 0.0197 |
| *NAME* | CLARITI | CODIFAR | -0.1078 | 0.04550 | 22 | -2.37 | 0.0271 | Tukey | 0.4632 |
| *NAME* | CLARITI | CODISCO | 0.05786 | 0.04550 | 22 | 1.27 | 0.2168 | Tukey | 0.9747 |
| *NAME* | CLARITI | CRAZI | -0.06049 | 0.04550 | 22 | -1.33 | 0.1973 | Tukey | 0.9655 |
| *NAME* | CLARITI | FERGUS | 0.07897 | 0.04550 | 22 | 1.74 | 0.0966 | Tukey | 0.8337 |
| *NAME* | CLARITI | HUSKI | -0.03132 | 0.04550 | 22 | -0.69 | 0.4985 | Tukey | 0.9999 |
| *NAME* | CLARITI | JUSTINA | 0.1166 | 0.04550 | 22 | 2.56 | 0.0178 | Tukey | 0.3552 |
| *NAME* | CLARITI | LAKTI | -0.09377 | 0.04550 | 22 | -2.06 | 0.0513 | Tukey | 0.6520 |
| *NAME* | CLARITI | P329D60 | -0.00068 | 0.04550 | 22 | -0.01 | 0.9882 | Tukey | 1.0000 |
| *NAME* | CLARITI | PICKER | -0.08630 | 0.04550 | 22 | -1.90 | 0.0711 | Tukey | 0.7495 |
| *NAME* | CLARITI | PR29B29 | -0.08197 | 0.04550 | 22 | -1.80 | 0.0853 | Tukey | 0.8011 |
| *NAME* | CODIFAR | CODISCO | 0.1656 | 0.04550 | 22 | 3.64 | 0.0014 | Tukey | 0.0498 |
| *NAME* | CODIFAR | CRAZI | 0.04726 | 0.04550 | 22 | 1.04 | 0.3102 | Tukey | 0.9946 |
| *NAME* | CODIFAR | FERGUS | 0.1867 | 0.04550 | 22 | 4.10 | 0.0005 | Tukey | 0.0183 |
| *NAME* | CODIFAR | HUSKI | 0.07644 | 0.04550 | 22 | 1.68 | 0.1071 | Tukey | 0.8591 |
| *NAME* | CODIFAR | JUSTINA | 0.2243 | 0.04550 | 22 | 4.93 | <.0001 | Tukey | 0.0028 |
| *NAME* | CODIFAR | LAKTI | 0.01398 | 0.04550 | 22 | 0.31 | 0.7615 | Tukey | 1.0000 |
| *NAME* | CODIFAR | P329D60 | 0.1071 | 0.04550 | 22 | 2.35 | 0.0280 | Tukey | 0.4720 |
| *NAME* | CODIFAR | PICKER | 0.02145 | 0.04550 | 22 | 0.47 | 0.6419 | Tukey | 1.0000 |
| *NAME* | CODIFAR | PR29B29 | 0.02578 | 0.04550 | 22 | 0.57 | 0.5766 | Tukey | 1.0000 |
| *NAME* | CODISCO | CRAZI | -0.1183 | 0.04550 | 22 | -2.60 | 0.0163 | Tukey | 0.3354 |
| *NAME* | CODISCO | FERGUS | 0.02111 | 0.04550 | 22 | 0.46 | 0.6472 | Tukey | 1.0000 |
| *NAME* | CODISCO | HUSKI | -0.08917 | 0.04550 | 22 | -1.96 | 0.0628 | Tukey | 0.7130 |
| *NAME* | CODISCO | JUSTINA | 0.05872 | 0.04550 | 22 | 1.29 | 0.2102 | Tukey | 0.9719 |
| *NAME* | CODISCO | LAKTI | -0.1516 | 0.04550 | 22 | -3.33 | 0.0030 | Tukey | 0.0929 |
| *NAME* | CODISCO | P329D60 | -0.05854 | 0.04550 | 22 | -1.29 | 0.2116 | Tukey | 0.9725 |
| *NAME* | CODISCO | PICKER | -0.1442 | 0.04550 | 22 | -3.17 | 0.0045 | Tukey | 0.1276 |
| *NAME* | CODISCO | PR29B29 | -0.1398 | 0.04550 | 22 | -3.07 | 0.0056 | Tukey | 0.1523 |
| *NAME* | CRAZI | FERGUS | 0.1395 | 0.04550 | 22 | 3.07 | 0.0057 | Tukey | 0.1545 |
| *NAME* | CRAZI | HUSKI | 0.02917 | 0.04550 | 22 | 0.64 | 0.5280 | Tukey | 0.9999 |
| *NAME* | CRAZI | JUSTINA | 0.1771 | 0.04550 | 22 | 3.89 | 0.0008 | Tukey | 0.0291 |
| *NAME* | CRAZI | LAKTI | -0.03328 | 0.04550 | 22 | -0.73 | 0.4722 | Tukey | 0.9998 |
| *NAME* | CRAZI | P329D60 | 0.05981 | 0.04550 | 22 | 1.31 | 0.2022 | Tukey | 0.9681 |
| *NAME* | CRAZI | PICKER | -0.02581 | 0.04550 | 22 | -0.57 | 0.5763 | Tukey | 1.0000 |
| *NAME* | CRAZI | PR29B29 | -0.02148 | 0.04550 | 22 | -0.47 | 0.6416 | Tukey | 1.0000 |
| *NAME* | FERGUS | HUSKI | -0.1103 | 0.04550 | 22 | -2.42 | 0.0240 | Tukey | 0.4308 |
| *NAME* | FERGUS | JUSTINA | 0.03761 | 0.04550 | 22 | 0.83 | 0.4174 | Tukey | 0.9992 |
| *NAME* | FERGUS | LAKTI | -0.1727 | 0.04550 | 22 | -3.80 | 0.0010 | Tukey | 0.0357 |
| *NAME* | FERGUS | P329D60 | -0.07965 | 0.04550 | 22 | -1.75 | 0.0939 | Tukey | 0.8266 |
| *NAME* | FERGUS | PICKER | -0.1653 | 0.04550 | 22 | -3.63 | 0.0015 | Tukey | 0.0505 |
| *NAME* | FERGUS | PR29B29 | -0.1609 | 0.04550 | 22 | -3.54 | 0.0019 | Tukey | 0.0616 |
| *NAME* | HUSKI | JUSTINA | 0.1479 | 0.04550 | 22 | 3.25 | 0.0037 | Tukey | 0.1091 |
| *NAME* | HUSKI | LAKTI | -0.06245 | 0.04550 | 22 | -1.37 | 0.1837 | Tukey | 0.9573 |
| *NAME* | HUSKI | P329D60 | 0.03064 | 0.04550 | 22 | 0.67 | 0.5077 | Tukey | 0.9999 |
| *NAME* | HUSKI | PICKER | -0.05498 | 0.04550 | 22 | -1.21 | 0.2397 | Tukey | 0.9825 |
| *NAME* | HUSKI | PR29B29 | -0.05065 | 0.04550 | 22 | -1.11 | 0.2776 | Tukey | 0.9906 |
| *NAME* | JUSTINA | LAKTI | -0.2103 | 0.04550 | 22 | -4.62 | 0.0001 | Tukey | 0.0057 |
| *NAME* | JUSTINA | P329D60 | -0.1173 | 0.04550 | 22 | -2.58 | 0.0172 | Tukey | 0.3475 |
| *NAME* | JUSTINA | PICKER | -0.2029 | 0.04550 | 22 | -4.46 | 0.0002 | Tukey | 0.0083 |
| *NAME* | JUSTINA | PR29B29 | -0.1985 | 0.04550 | 22 | -4.36 | 0.0002 | Tukey | 0.0102 |
| *NAME* | LAKTI | P329D60 | 0.09309 | 0.04550 | 22 | 2.05 | 0.0529 | Tukey | 0.6611 |
| *NAME* | LAKTI | PICKER | 0.007471 | 0.04550 | 22 | 0.16 | 0.8711 | Tukey | 1.0000 |
| *NAME* | LAKTI | PR29B29 | 0.01180 | 0.04550 | 22 | 0.26 | 0.7977 | Tukey | 1.0000 |
| *NAME* | P329D60 | PICKER | -0.08562 | 0.04550 | 22 | -1.88 | 0.0732 | Tukey | 0.7579 |
| *NAME* | P329D60 | PR29B29 | -0.08129 | 0.04550 | 22 | -1.79 | 0.0878 | Tukey | 0.8087 |
| *NAME* | PICKER | PR29B29 | 0.004332 | 0.04550 | 22 | 0.10 | 0.9250 | Tukey | 1.0000 |


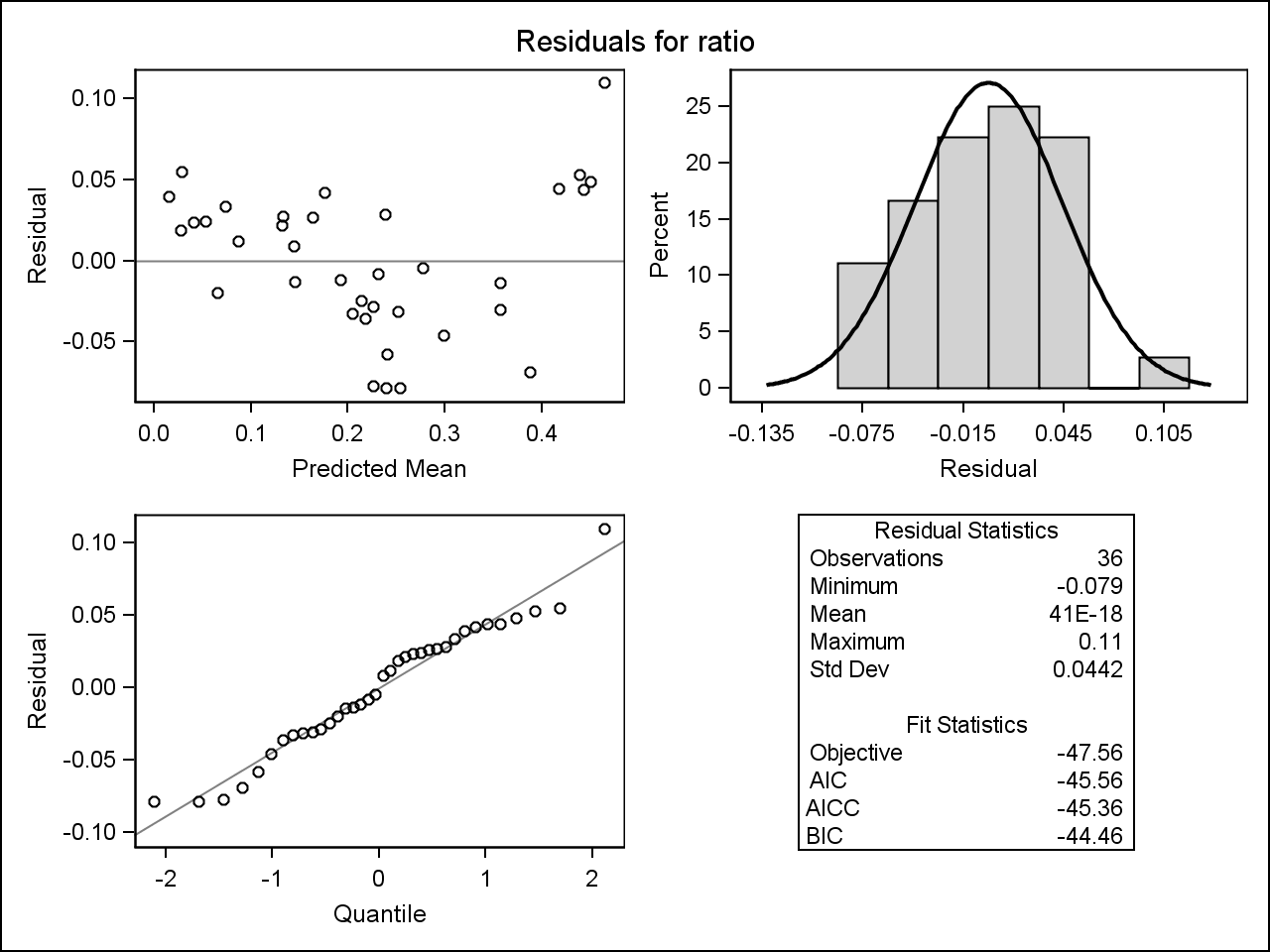


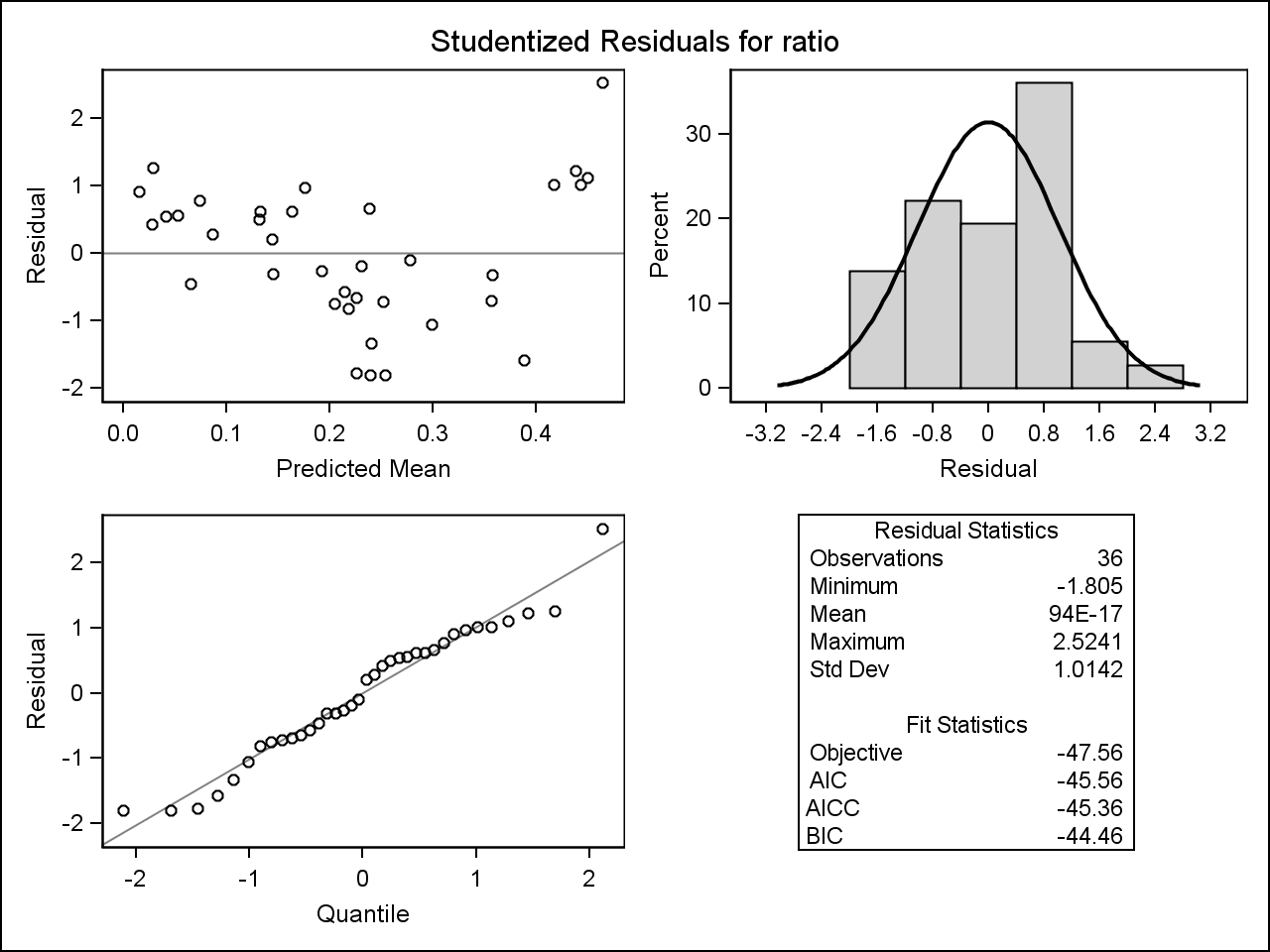


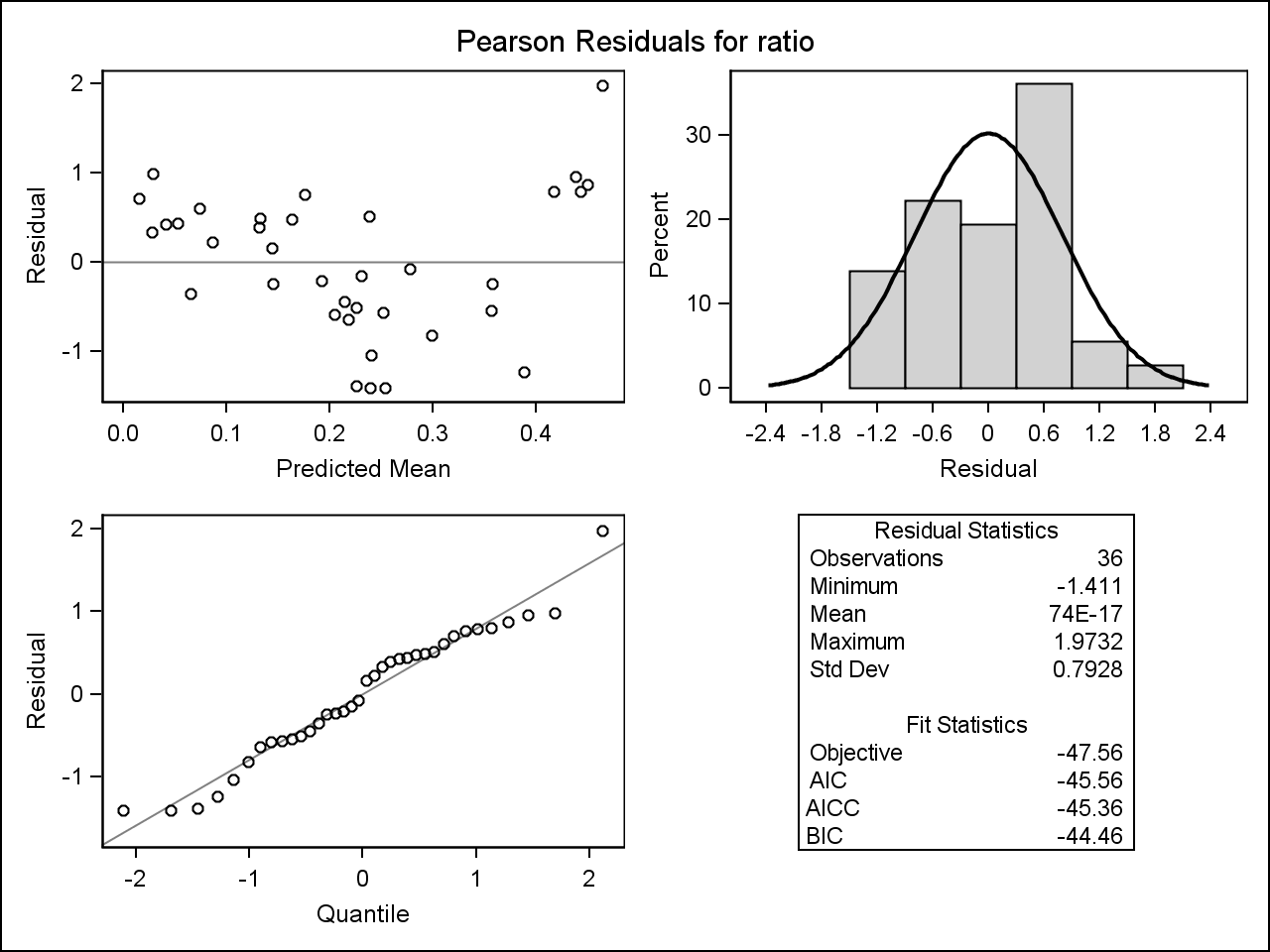

Supplement: Table S2 [file peerj-05-2839-s002.docx]
