# Supplementary material for: Transcriptomic response of maize primary roots to low temperatures at seedling emergence
Source: PeerJ. 2017 Jan 5;5:e2839. doi: 10.7717/peerj.2839 (PMC5289442; doi:10.7717/peerj.2839)
Supplement: Table S3 [file peerj-05-2839-s003.docx]

**Supplemental Materials Table 3.** Housekeeping gene information: Name of gene, accession number and bibliographic reference.

| **Name** | **Accession No.** | **References** |
| --- | --- | --- |
| Actin | AY104722 | Nguyen, H. T., Leipner, J., Stamp, P. & Guerra-Peraza, O. Low temperature stress in maize (*Zea Mays* L.) induces genes involved in photosynthesis and signal transduction as studied by suppression subtractive hybridization. *Plant Physiol. Biochem.* **47,** 116-122 (2009) |
| Adh1 | X04050 | Hernandez, M., Duplan, M. N., Berthier, G., Vaitilingom, M., Hauser, W., Freyer, R., Pla, M. & Bertheau, Y. Development and comparison of four Real-Time Polymerase Chain Reaction Systems for specific detection and quantification of *Zea mays* L. *J. Agric. Food Chem.* **52,** 4632-4637 (2004) |
| Ef1-α | CV071927 | Brunner, A. M., Yakovlev, I. A. & Strauss, S. H. Validating internal controls for quantitative plant gene expression studies. *BMC Plant Biology* **8,** 4:14 (2004) |
| Tua5 | EB822881 | Santi, S., Locci, G., Monte, R., Pinton, R. & Varanini, Z. Induction of nitrate uptake in maize roots: expression of a putative high-affinity nitrate transporter and plasma membrane H^+^-ATPase isoforms. *J. Exp. Bot.* **54,** 1851-1864 (2003) |
