# Supplementary material for: Transcriptomic response of maize primary roots to low temperatures at seedling emergence
Source: PeerJ. 2017 Jan 5;5:e2839. doi: 10.7717/peerj.2839 (PMC5289442; doi:10.7717/peerj.2839)
Supplement: Table S4 — Sequences of housekeeping genes and differential expressed candidate genes. [file peerj-05-2839-s004.docx]

Supplemental Materials Table 4: Primers used for qRT-PCR. Sequences of housekeeping genes and differential expressed candidate genes.

| **Name** | **Forward sequence** | **Reverse sequence** |
| --- | --- | --- |
| Actin | TCTGCTGAACGCGAAATTGT | ACAGATGAGCTGCTCTTGGCA |
| GAPDH | ACTGTTCATGCCATCACTGC | GAGGACAGGAAGCACTTTGC |
| ZmUBI | GGTGGTATGCAGATCTTT | GTAGTCTGCTAGGGTGCG |
| Adh1 | CGTCGTTTCCCATCTCTTCCTCC | CCACTCCGAGACCCTCAGTC |
| Hmgp | TTGGACTAGAAATCTCGTGCTGA | GCTACATAGGGAGCCTTGTCCT |
| Invr1 | CGCTCTGTACAAGCGTGC | GCAAAGTGTTGTGCTTGGACC |
| Zein | GCCATTGGGTACCATGAACC | AGGCCAACAGTTGCTGCAG |
| Cyclophilin | TGTGAACCGATTTAGGCACA | CGCTGTCGTCAACTTATCCA |
| Ef1-α | GGCTGGCACTATCAGAGAGG | CACGACGTACTTTGCTCTCG |
| γ-tubulin | ACATCATTCAAGGGGAGGTG | CTTGCAGGTGCCCAGTCTAT |
| Thioredoxin | ACCCCGACATTCTTCTTCCT | CGGCTGCTAGCACTTTCTTC |
| Tua5 | GTCGACCTTGAACCCACTGT | GTTAGCTGCGTCTTCCTTGC |
| MZ00003507 | TTCGGATCCTGTCTTCCATC | ACTCCACGAAAGCATCAAGG |
| MZ00004486 | GAGCAACGTGATTGGACAGA | GCATATGCGGGGAGAACATA |
| MZ00022876 | GGAAGGTGTGTTCTGCGTTT | TATGAAGCTGACTGGCGTTG |
| MZ00041708 | TTGCTGCTCTTCACCTTCCT | ACAACAATGGCTTCCCTGAA |
| MZ00023411 | GCACCACGAGAAGAAGAAGG | CACAAGCAAGCAGCTACCAA |
| MZ00026737 | AGAAGGCCATGGTCAAGATG | GCTTGCAGGTCATGAACAAA |
| MZ00029223 | CGAAGATCAAGGACGAGGAG | ACGACTGCTCTGCAACAGAA |
| MZ00026029 | GCTACATCAACAGCCCCAAC | TACACGACAACACGACACGA |
| MZ00037140 | AAGTAAGGCCACGCGTTTTA | CCTGGACTACGAGACGGTGT |
