# Supplementary material for: Transcriptomic response of maize primary roots to low temperatures at seedling emergence
Source: PeerJ. 2017 Jan 5;5:e2839. doi: 10.7717/peerj.2839 (PMC5289442; doi:10.7717/peerj.2839)
Supplement: Table S5 [file peerj-05-2839-s005.docx]

**Supplemental Materials Table 5. Differential expressed genes in cultivar PR39B29**

| \| **ID** \| **Name** \| **BH** \| **meanM** \| **meanA** \| **Gene product** \| \| --- \| --- \| --- \| --- \| --- \| --- \| \| MZ00001648 \| AW566370 \| 0.016133335 \| 2.75183151 \| 7.988158308 \| NA \| \| MZ00003507 \| BE130044 \| 0.013891921 \| -2.785514086 \| 10.80585389 \| NA \| \| MZ00004486 \| TC253575 \| 0.049682283 \| -2.422917241 \| 9.353119581 \| pathogenesis related protein-1 - maize {*Zea mays*;} ^\|^GB\|AAC25629.1\|3290004\|ZMU82200 pathogenesis related protein-1 {*Zea mays*;} \| \| MZ00004711 \| BM080031 \| 0.019278631 \| -2.713831389 \| 9.7088997 \| putative peroxidase {*Oryza sativa* (japonica cultivar-group);} ^\|^GB\|BAA96643.1\|8468043\|AP002482 unnamed protein product; Similar to Arabidopsis thaliana peroxidase ATP19a (X98805) {*Oryza sativa* (japonica cul-TRUNCATED- \| \| MZ00013139 \| AZM4_69070 \| 0.022134955 \| -2.682380809 \| 8.609035993 \| contains ESTs AU064445(E30981),AU033124(S3631) similar to *Arabidopsis thaliana* chromosome 5, At5g44680 unknown protein {*Oryza sativa* (*japonica* cultivar-group);} \| \| MZ00013532 \| TC255153 \| 0.002543002 \| 3.170330016 \| 11.7699957 \| Phosphoenolpyruvate carboxykinase [ATP] (EC 4.1.1.49) (PEPcarboxykinase) (Phosphoenolpyruvate carboxylase) (PEPCK). {*Zea mays*;} ^\|^GB\|BAA36483.1\|5672673\|AB018744 phosphoenolpyruvate carboxykinase {*Zea mays*;}-TRUNCATED- \| \| MZ00014008 \| TC260104 \| 0.012623595 \| -2.811178625 \| 10.00082487 \| Farnesyl pyrophosphate synthetase (FPP synthetase) (FPS) (Farnesyldiphosphate synthetase) [Includes: Dimethylallyltransferase(EC 2.5.1.1); Geranyltranstransferase (EC 2.5.1.10)]. {*Zea mays*;} ^\|^PIR\|T03291\|T0-TRUNCATED- \| \| MZ00014434 \| TC260325 \| 0.000129937 \| 3.751106577 \| 10.05396971 \| hypothetical protein At2g38110 [imported] - *Arabidopsis thaliana* {*Arabidopsis thaliana*;} ^\|^GB\|AAM47976.1\|21387145\|AY114657 unknown protein {*Arabidopsis thaliana*;} ^\|^GB\|AAL32799.1\|17065290\|AY062721 Unknown -TRUNCATED- \| \| MZ00015899 \| TC259585 \| 0.006358762 \| 2.965902453 \| 12.76585935 \| cinnamoyl CoA reductase - maize {*Zea mays*;} ^\|^GB\|CAA74071.1\|2239260\|ZMCCRGENE cinnamoyl CoA reductase {*Zea mays*;} \| \| MZ00016581 \| AZM4_21980 \| 0.049385887 \| 2.472624651 \| 11.35623272 \| NOD26-like membrane integral protein ZmNIP2-1 {*Zea mays*;} \| \| MZ00017520 \| TC259837 \| 0.010923079 \| 2.844940859 \| 12.08245555 \| unknown protein {*Oryza sativa* (japonica cultivar-group);} \| \| MZ00017851 \| TC263371 \| 0.007998279 \| 2.917566485 \| 13.39381452 \| O-methyltransferase ZRP4 (EC 2.1.1.-) (OMT). {*Zea mays*;} ^\|^PIR\|JQ2268\|JQ2268 O-methyltransferase (EC 2.1.1.-) - maize {*Zea mays*;} ^\|^GB\|AAA18532.1\|404070\|MZEOMT O-methyltransferase {*Zea mays*;} \| \| MZ00019060 \| TC251990 \| 0.049385887 \| 2.467309502 \| 8.997125503 \| putative syntaxin SYP111 {*Oryza sativa* (japonica cultivar-group);} \| \| MZ00022590 \| TC256960 \| 0.049682283 \| 2.418472846 \| 9.702906078 \| putative NAC domain protein NAC1 {*Oryza sativa* (japonica cultivar-group);} ^\|^GB\|BAD03222.1\|38636961\|AP004589 putative NAC domain protein NAC1 {*Oryza sativa* (japonica cultivar-group);} \| \| MZ00022876 \| TC254729 \| 0.032123812 \| 2.580961628 \| 9.597265501 \| hypothetical protein {*Oryza sativa* (japonica cultivar-group);} \| \| MZ00023132 \| TC267941 \| 0.049614723 \| 2.449509053 \| 9.122439177 \| DUR3 {*Oryza sativa* (japonica cultivar-group);} ^\|^GB\|AAP55189.1\|31433710\|AE017123 putative urea active transport protein {*Oryza sativa* (japonica cultivar-group);} ^\|^GB\|AAG46170.1\|12039384\|AC018727 putative -TRUNCATED- \| \| MZ00023411 \| TC258611 \| 0.049385887 \| 2.458064533 \| 11.87385832 \| 22 kDa drought-inducible protein {*Saccharum* hybrid cultivar;} ^\|^GB\|BAB68268.1\|15667623\|AB071694 drought inducible 22 kD protein {*Saccharum officinarum*;} \| \| MZ00026598 \| TC272701 \| 0.002733806 \| -3.138709306 \| 8.385784504 \| NA \| \| MZ00026737 \| TC272703 \| 0.034951693 \| 2.552428577 \| 11.62432469 \| peroxidase {*Zea mays*;} \| \| MZ00029223 \| TC253998 \| 0.049385887 \| -2.469323736 \| 11.38138719 \| putative heat shock protein hsp22 precursor {*Oryza sativa* (japonica cultivar-group);} \| \| MZ00029584 \| TC213104 \| 0.049682283 \| 2.415941463 \| 8.882360354 \| arogenate dehydrogenase {*Arabidopsis thaliana*;} \| \| MZ00030597 \| TC254547 \| 0.045608153 \| 2.500276217 \| 10.72619731 \| hypothetical protein {*Oryza sativa* (japonica cultivar-group);} ^\|^GB\|BAD09400.1\|42408243\|AP004462 hypothetical protein {*Oryza sativa* (japonica cultivar-group);} \| \| MZ00031971 \| TC249091 \| 0.049682283 \| -2.414466039 \| 8.30601041 \| chloroplast RelA homologue 1 {*Oryza sativa* (japonica cultivar-group);} \| \| MZ00032181 \| TC256231 \| 0.027204005 \| 2.623466647 \| 9.141974431 \| unknown protein {*Oryza sativa* (japonica cultivar-group);} ^\|^GB\|BAD33551.1\|50726026\|AP005551 unknown protein {*Oryza sativa* (japonica cultivar-group);} \| \| MZ00032300 \| TC267215 \| 0.032123812 \| -2.578920008 \| 8.938059247 \| NA \| \| MZ00032919 \| TC275843 \| 0.002260487 \| 3.24228398 \| 11.79219444 \| putative metacaspase, having alternative splicing products {*Oryza sativa* (japonica cultivar-group);} \| \| MZ00037107 \| BM380133 \| 0.049682283 \| 2.414317539 \| 10.50393443 \| NA \| \| MZ00039384 \| CD999219 \| 0.003868766 \| 3.053696635 \| 12.08876037 \| NA \| \| MZ00041297 \| TC258767 \| 0.027204005 \| 2.626008085 \| 12.58611071 \| NA \| \| MZ00041306 \| TC258637 \| 0.049682283 \| 2.425199198 \| 12.43862438 \| putative ASR2 {*Oryza sativa* (japonica cultivar-group);} \| \| MZ00041333 \| TC249511 \| 0.008256155 \| 2.899706289 \| 13.36644993 \| legumain-like protease {*Zea mays*;} \| \| MZ00041334 \| TC192088 \| 0.032123812 \| 2.573538496 \| 13.11130824 \| C13 endopeptidase NP1 precursor {*Zea mays*;} \| \| MZ00041500 \| AZM4_134720 \| 0.002160929 \| 3.287279627 \| 11.28065105 \| ESTs AU078183(C62904),C73912(E21020) correspond to a region of the predicted gene. Similar to water stress inducible protein (U74296) {*Oryza sativa* (japonica cultivar-group);} \| \| MZ00041708 \| TC193433 \| 0.049385887 \| 2.455886831 \| 10.296384 \| contains EST C99107(E4452) unknown protein {*Oryza sativa* (japonica cultivar-group);} ^\|^GB\|BAB90465.1\|20161543\|AP003725 contains EST C99107(E4452) unknown protein {*Oryza sativa* (japonica cultivar-group);} \| \| MZ00042906 \| TC269711 \| 0.003868766 \| 3.070249006 \| 11.7173933 \| r40g3 protein - rice {*Oryza sativa*;} ^\|^GB\|BAC83806.1\|34394519\|AP005167 r40g3 protein {*Oryza sativa* (japonica cultivar-group);} ^\|^GB\|CAA70175.1\|1658315\|OSR40G3 osr40g3 {*Oryza sativa* (indica cultivar-group);-TRUNCATED- \| \| MZ00042908 \| TC269712 \| 0.000129937 \| 3.69588019 \| 11.73932564 \| r40g3 protein - rice {*Oryza sativa*;} ^\|^GB\|BAC83806.1\|34394519\|AP005167 r40g3 protein {*Oryza sativa* (japonica cultivar-group);} ^\|^GB\|CAA70175.1\|1658315\|OSR40G3 osr40g3 {*Oryza sativa* (indica cultivar-group);-TRUNCATED- \| \| MZ00043603 \| TC258422 \| 0.049682283 \| 2.440130314 \| 9.748619496 \| aspartic proteinase {*Oryza sativa* (japonica cultivar-group);} \| \| MZ00044067 \| TC272779 \| 0.002420877 \| 3.202355349 \| 10.69218418 \| NA \| \| MZ00056611 \| TC254030 \| 0.022909948 \| -2.668023701 \| 12.02370141 \| NA \| |
| --- | --- | --- | --- | --- | --- | --- | --- | --- | --- | --- | --- | --- | --- | --- | --- | --- | --- | --- | --- | --- | --- | --- | --- | --- | --- | --- | --- | --- | --- | --- | --- | --- | --- | --- | --- | --- | --- | --- | --- | --- | --- | --- | --- | --- | --- | --- | --- | --- | --- | --- | --- | --- | --- | --- | --- | --- | --- | --- | --- | --- | --- | --- | --- | --- | --- | --- | --- | --- | --- | --- | --- | --- | --- | --- | --- | --- | --- | --- | --- | --- | --- | --- | --- | --- | --- | --- | --- | --- | --- | --- | --- | --- | --- | --- | --- | --- | --- | --- | --- | --- | --- | --- | --- | --- | --- | --- | --- | --- | --- | --- | --- | --- | --- | --- | --- | --- | --- | --- | --- | --- | --- | --- | --- | --- | --- | --- | --- | --- | --- | --- | --- | --- | --- | --- | --- | --- | --- | --- | --- | --- | --- | --- | --- | --- | --- | --- | --- | --- | --- | --- | --- | --- | --- | --- | --- | --- | --- | --- | --- | --- | --- | --- | --- | --- | --- | --- | --- | --- | --- | --- | --- | --- | --- | --- | --- | --- | --- | --- | --- | --- | --- | --- | --- | --- | --- | --- | --- | --- | --- | --- | --- | --- | --- | --- | --- | --- | --- | --- | --- | --- | --- | --- | --- | --- | --- | --- | --- | --- | --- | --- | --- | --- | --- | --- | --- | --- | --- | --- | --- | --- | --- | --- | --- | --- | --- | --- | --- | --- | --- | --- | --- | --- | --- | --- | --- | --- | --- | --- | --- | --- |
