# Supplementary material for: Transcriptomic response of maize primary roots to low temperatures at seedling emergence
Source: PeerJ. 2017 Jan 5;5:e2839. doi: 10.7717/peerj.2839 (PMC5289442; doi:10.7717/peerj.2839)
Supplement: Table S6 [file peerj-05-2839-s006.docx]

**Supplemental Materials Table 6. Differential expressed genes in cultivar Picker**

| **ID** | **Name** | **BH** | **meanM** | **meanA** | **Gene product** |
| --- | --- | --- | --- | --- | --- |
| MZ00003507 | BE130044 | 0.010536135 | 2.430596314 | 10.59109127 | NA |
| MZ00004486 | TC253575 | 9.70E-07 | -3.662902715 | 9.860354421 | NA |
| MZ00005992 | TC248783 | 0.000639912 | -2.895167859 | 10.97397294 | ribulosebisphosphate carboxylase. {*Zea mays*;} |
| MZ00012575 | TC268661 | 0.010536135 | -2.443757757 | 11.91439996 | NA |
| MZ00012962 | CF637427 | 0.006767432 | -2.516076271 | 9.346205616 | unknown protein {*Oryza sativa* (japonica cultivar-group);} ^\|^GB\|BAD28155.1\|50252159\|AP004858 unknown protein {*Oryza sativa* (japonica cultivar-group);} |
| MZ00014087 | TC249270 | 0.003077634 | -2.692489724 | 11.05200993 | putative small nuclear ribonucleoprotein polypeptide F {*Oryza sativa* (japonica cultivar-group);} |
| MZ00015009 | TC271061 | 0.038825334 | 2.201849278 | 9.26355321 | root-specific protein RCc3 - rice {*Oryza sativa*;} ^\|^GB\|BAD25630.1\|49388506\|AP004883 root-specific protein RCc3 {*Oryza sativa* (japonica cultivar-group);} ^\|^GB\|BAD27673.1\|50251740\|AP004037 root-specific prot-TRUNCATED- |
| MZ00015350 | TC261342 | 2.34E-05 | -3.269660613 | 8.418066204 | NA |
| MZ00017067 | TC272126 | 2.23E-05 | 3.296707092 | 8.870984596 | putative 40S ribosomal protein {*Oryza sativa* (japonica cultivar-group);} ^\|^GB\|BAC84635.1\|34394183\|AP005895 putative 40S ribosomal protein {*Oryza sativa* (japonica cultivar-group);} ^\|^GB\|AAK55780.1\|14192875\|-TRUNCATED- |
| MZ00018470 | TC253537 | 0.003528171 | -2.644930189 | 10.18722448 | unknown {*Arabidopsis thaliana*;} ^\|^GB\|AAL34283.1\|17104789\|AY063109 unknown protein {*Arabidopsis thaliana*;} ^\|^GB\|AAK44136.1\|13878117\|AF370321 unknown protein {*Arabidopsis thaliana*;} ^\|^GB\|BAB09455.1\|9758918\|-TRUNCATED- |
| MZ00022876 | TC254729 | 0.004171029 | 2.611015808 | 8.659284425 | hypothetical protein {*Oryza sativa* (japonica cultivar-group);} |
| MZ00026029 | TC251197 | 0.038825334 | -2.168930948 | 9.817004058 | probable lipid transfer protein - rice {*Oryza sativa*;} ^\|^GB\|AAC50030.1\|1041815\|OSU16721 lipid transfer protein {*Oryza sativa* (indica cultivar-group);} |
| MZ00026299 | TC272358 | 0.038825334 | -2.1716173 | 12.02925934 | putative peroxidase P7X {*Zea mays*;} |
| MZ00027910 | TC252829 | 0.038825334 | -2.186934994 | 10.75159673 | putative chitinase {*Oryza sativa* (japonica cultivar-group);} ^\|^GB\|BAD09686.1\|42408507\|AP004622 putative chitinase {*Oryza sativa* (japonica cultivar-group);} |
| MZ00029892 | TC262888 | 0.022417812 | -2.303817119 | 11.65047663 | putative MtN21 {*Oryza sativa* (japonica cultivar-group);} |
| MZ00031132 | TC256120 | 0.043624559 | 2.146391884 | 9.825596509 | NA |
| MZ00031569 | TC255672 | 0.005683894 | 2.548818433 | 8.375946625 | putative PGPD14 protein (pollen germination related protein) {*Oryza sativa* (japonica cultivar-group);} |
| MZ00032161 | TC268195 | 0.01247776 | -2.399735499 | 11.23807926 | root cap protein 2 {*Zea mays*;} |
| MZ00034859 | rpl16 | 0.03594631 | -2.222307277 | 9.004852233 | Chloroplast 50S ribosomal protein L16. {*Zea mays*;} ^\|^PIR\|S58589\|S58589 ribosomal protein L16 - maize chloroplast {*Zea mays*;} ^\|^GB\|CAA60323.1\|902259\|ZMA86563 ribosomal protein L16 {Zea mays;} |
| MZ00036678 | TC273501 | 0.038825334 | -2.170276261 | 13.35864732 | contains ESTs AU097436(S4951),D41930(S4951),AU101944(S2163),AU095016 (E50186),AU058216(E50186),C72995(E2631),D40291(S2163), D41387(S3866) unknown protein {*Oryza sativa* (japonica cultivar-group);}-TRUNCATED- |
| MZ00037140 | BM381350 | 6.23E-06 | -3.450679372 | 11.23679534 | glucose starvation-induced protein precursor (clone pZSS2) - maize {*Zea mays*;} ^\|^GB\|CAA57674.1\|559534\|ZMDRP defence-related protein {*Zea mays*;} |
| MZ00040527 | rpl2 | 0.035678055 | -2.230441995 | 11.07648127 | ribosomal protein L2 {*Zea mays*;} |
| MZ00040530 | NB_con:1103.m00114\|NB:1100.m01418\|CMSC:1105.m00576 | 0.010536135 | -2.432128714 | 12.63411453 | hypothetical protein {*Zea mays*;} |
| MZ00040560 | NP003805\|X82184.1\|CAA57674.1 | 0.003182323 | -2.672113829 | 9.86437317 | glucose starvation-induced protein precursor (clone pZSS2) - maize {*Zea mays*;} ^\|^GB\|CAA57674.1\|559534\|ZMDRP defence-related protein {*Zea mays*;} |
| MZ00041588 | TC260396 | 0.038825334 | -2.171194017 | 12.38087896 | contains ESTs AU064445(E30981),AU033124(S3631) similar to *Arabidopsis thaliana* chromosome 5, At5g44680 unknown protein {*Oryza sativa* (japonica cultivar-group);} |
| MZ00041708 | TC193433 | 0.038825334 | 2.184045686 | 10.16071255 | contains EST C99107(E4452) unknown protein {*Oryza sativa* (japonica cultivar-group);} ^\|^GB\|BAB90465.1\|20161543\|AP003725 contains EST C99107(E4452) unknown protein {*Oryza sativa* (japonica cultivar-group);} |
| MZ00043117 | TC250981 | 0.004224242 | -2.597475727 | 11.56106454 | OSJNBa0008M17.8 {*Oryza sativa* (japonica cultivar-group);} |
| MZ00045615 | gi\|13377834\|gb\|AF334169.1\|AF334169 | 0.019991549 | -2.32734359 | 10.72253524 | NA |
| MZ00049724 | AZM4_41210 | 5.71E-11 | -4.517533173 | 7.446206544 | BRAP2-like protein {*Oryza sativa* (japonica cultivar-group);} |
