# Supplementary material for: Transcriptomic response of maize primary roots to low temperatures at seedling emergence
Source: PeerJ. 2017 Jan 5;5:e2839. doi: 10.7717/peerj.2839 (PMC5289442; doi:10.7717/peerj.2839)
Supplement: Figure S7 [file peerj-05-2839-s007.pdf]

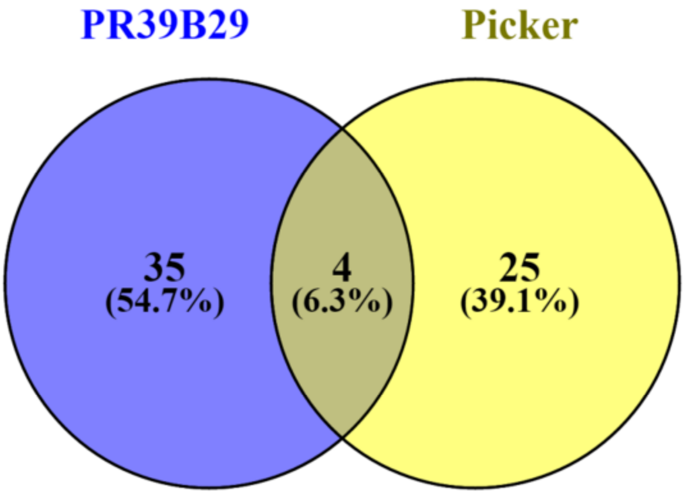

Supplemental Figure 1. Venn diagram of the number of differentially expressed and shared genes amongst maize varieties PR39B29 and Picker.
